# Artificial structures can facilitate rapid coral recovery under climate change

**DOI:** 10.1038/s41598-025-93531-2

**Published:** 2025-03-17

**Authors:** Toko Tanaya, Shunpei Iwamura, Wataru Okada, Tomohiro Kuwae

**Affiliations:** 1 Coastal and Estuarine Environment Research Group, Port and Airport Research Institute, 3-1-1 Nagase, Yokosuka, 239-0826 Japan; 2 Daiei Consultant Co., Ltd, 412-4 Minatogawa, Urasoe, 901-2134 Japan; 3 Incorporated Foundation Okinawa Prefecture Environment Science Center, 720 Kyozuka, Urasoe, 901-2111 Japan

**Keywords:** Ocean sciences, Restoration ecology

## Abstract

Rising seawater temperatures from climate change have caused coral bleaching, risking coral extinction by century’s end. To save corals, reef restoration must occur alongside other climate-change mitigation. Here we show the effectiveness of habitat creation on artificial structures for rapid coral restoration in response to climate change. We use 29 years of field observations for coral distributions on breakwaters and surrounding reefs (around 33,000 measurements in total). Following bleaching in 1998, breakwaters had higher coral cover (mainly *Acropora* spp.) than did surrounding natural reefs. Coral recovery times on breakwaters matched the frequency of recent bleaching events (~ every 6 years) and were accelerated by surface processing of the artificial structures with grooves. Corals on breakwaters were more abundant in shallow waters, under high light, and on moderately sloped substrate. Coral abundance on breakwaters was increased by incorporating shallow areas and surface texture. Our results suggest that habitat creation on artificial structures can increase coral community resilience against climate change by increasing coral recovery potential.

**Supplementary Information:**

The online version contains supplementary material available at 10.1038/s41598-025-93531-2.

## Introduction

Corals are keystone organisms of tropical and subtropical marine ecosystems. The economic value of ecosystem services provided by coral reef ecosystems is estimated to be the highest among all ecosystems^1^. However, coral reef ecosystems are extremely vulnerable to environmental change, especially in recent years when higher water temperatures associated with climate change have led to large-scale coral bleaching and severe ecosystem degradation^2^. Projections using the new generation of climate-change models (CMIP6) from the Intergovernmental Panel on Climate Change show that under a fossil-fuel-dependent scenario (Shared Socio-economic Pathway 5-8.5), all coral reefs will experience annual severe bleaching during this century^3^. To avert potential extirpation of coral reef ecosystems and recover them, climate-change mitigation is the first priority. However, to maintain and regenerate the functions of coral reef ecosystems, proactive measures to enhance their resilience must be taken in parallel with climate-change mitigation^4–6^. Several studies have pointed out that traditional local stress-reduction efforts alone, such as improving water quality, eradicating predators, and establishing marine protected areas, cannot prevent the degradation of coral reef ecosystems due to climate change^7–9^. This highlights the need to protect coral reef ecosystems from climate change by attempting to restore ecosystems through interventions—at the scales of individuals, communities, and ecosystems—that differ from traditional conservation measures^10–14^.

The addition of substrate (or “artificial reefs”)—i.e., deploying or building artificial structures as substrate for coral reef restoration through coral recruitment or coral planting—is one of the current methods for coral reef restoration, in addition to direct transplantation, coral gardening, substrate manipulation, and larval propagation^5,15^. Artificial reefs that function as coral habitat include structures installed on natural coral reefs as a substrate for the purpose of coral colonization^16–19^, as well as existing structures such as breakwaters, seawalls, shipwrecks, and energy platforms^20–27^. Some studies comparing artificial structures with surrounding natural coral communities have shown that the coral cover on mature artificial structures can be as high as, or higher than, that of natural reefs^21,23,24^. However, previous studies have not sufficiently validated the effectiveness of substrate addition by using artificial structures as a method for restoring coral habitat. To validate the effectiveness, we have measured the rate of coral recovery from a disturbance using the long-term monitoring data of coral communities on artificial structures. One reason is that there have been few long-term studies on the development of coral communities on artificial structures (the median length of monitoring periods is 12 months, and less than 10 years at most^15^). Because coral communities have been disturbed by high water temperatures at increasingly short time intervals (median time between severe bleaching events is 6 years since the 2000s^28^), information about the development rate of coral communities and their responses to disturbance is essential for the effective use of artificial structures to promote community recovery from disturbances. Although a few studies have reported the rate of initial community development^18,27^, few have reported the rate of recovery from a disturbance or the long-term behavior of communities beyond the early stages of community development. Another constraint is the limited knowledge of the environmental conditions suitable for coral growth on artificial structures. Previous studies have shown that water depth, substrate slope and orientation, wave exposure, water transparency, substrate surface texture, and substrate material affect the development of coral communities on artificial structures^20,24,26,27,29,30^. However, most studies have examined a limited range of these conditions and have not been able to fully characterize the conditions effective for promoting coral growth. Because coral community development is strongly influenced by environmental conditions that vary greatly in time and space, much empirical data from various locations over a long period of time are needed to compare the development processes of coral communities on artificial structures and natural reefs, and to verify the effectiveness of artificial structures as coral habitats.

**Fig. 1:**
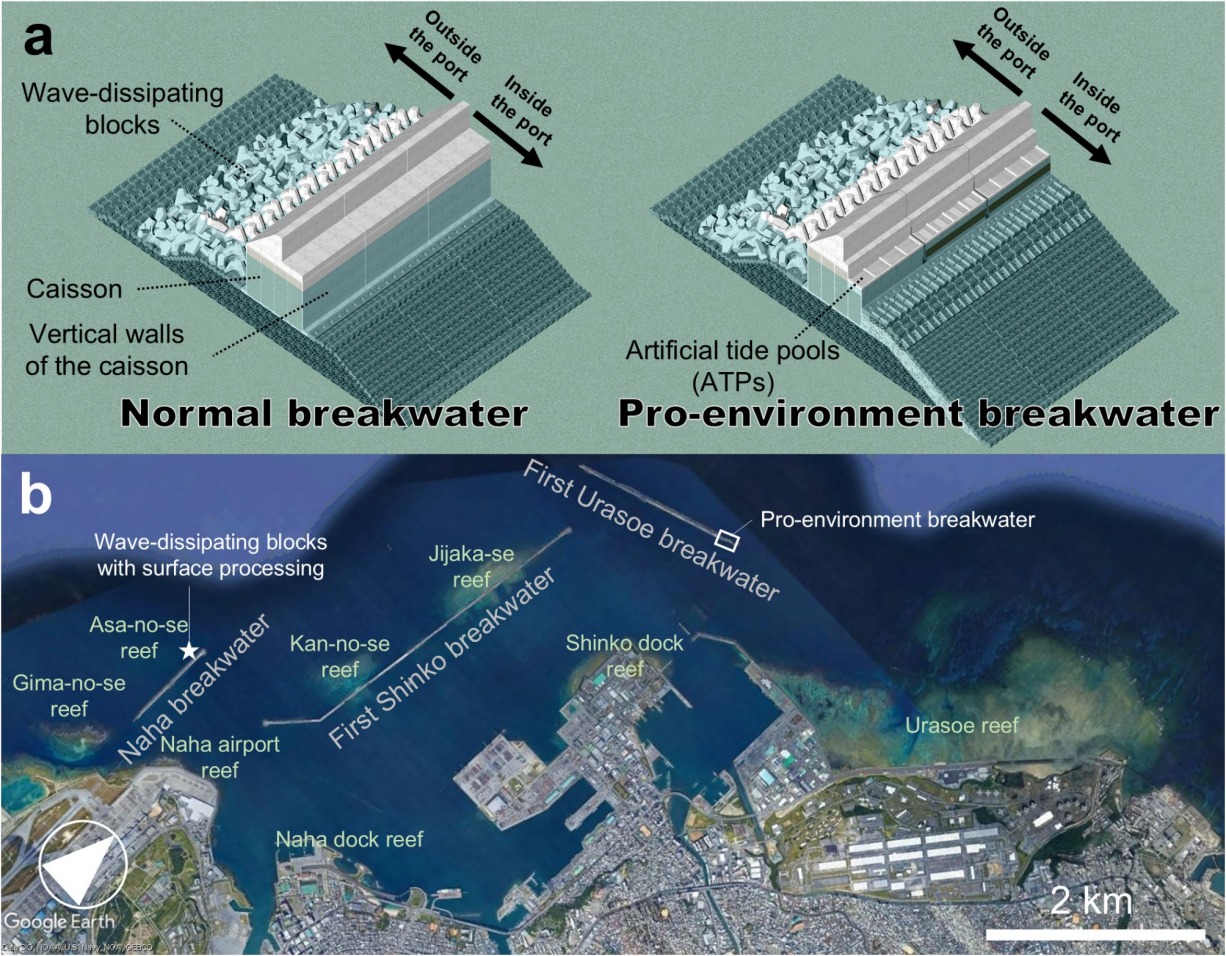
Main structure of the normal and pro-environment breakwaters and locations of the breakwaters and surrounding natural reefs. (**a**) Isometric view of a normal and a pro-environment breakwater, modified from Tanaya et al. (2021). Isometric view was created using the formZ 6.2 (https://www.ultimategraphics.co.jp/formz/products/formzpro.html) and the Autodesk 3ds MAX 2019 (https://www.autodesk.com/jp/products/3ds-max/overview?term=1-YEAR&tab=subscription). (**b**) Locations of artificial structures (breakwaters) and surrounding natural coral reefs in Naha Port, Okinawa, Japan, where the coral distribution surveys were conducted. The map was created using the Google Earth Pro 7.3.6.10201 (https://www.google.co.jp/intl/ja/earth/about/) and annotated using the Microsoft Office PowerPoint 2016 (https://www.microsoft.com).

This study describes coral growth (coral cover) on three artificial structures (breakwaters) and surrounding natural reefs in Naha Port, Okinawa, Japan, over the past 29 years. The approximately 33,000 data points obtained for coral cover and taxa were used to determine the environmental conditions suitable for coral growth on artificial structures. We examined coral and fish growth on a pro-environment breakwater^31^ appended to near the eastern end of the First Urasoe Breakwater and designed to incorporate environments favorable for coral growth, and on wave-dissipating blocks with surface processing installed on the Naha Breakwater (Fig. 1), to determine the effects of interventions on the coral reef ecosystem. In addition, by comparing coral growth on breakwaters and surrounding natural reefs, we verified the effectiveness of artificial structures in creating new habitats as a means of restoring coral reef ecosystems in response to climate change.

## Results

### Long-term coral distribution on breakwaters and surrounding natural reefs before and after a mass coral-bleaching event

**Fig. 2:**
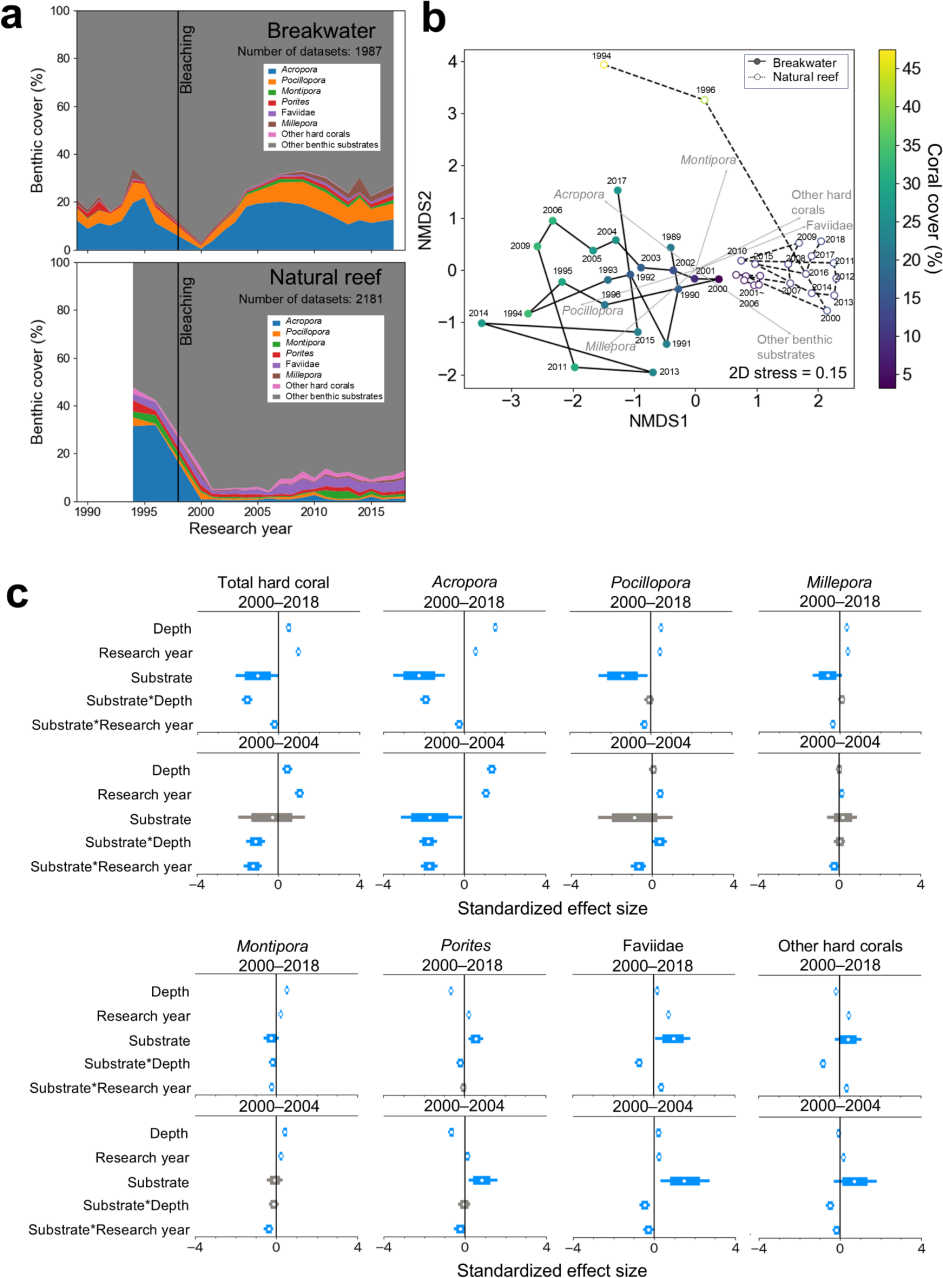
Percent cover of hard corals on breakwaters and surrounding natural reefs, and relationships among substrate type, research year, depth, and coral cover after bleaching. (**a**) Temporal dynamics in percent hard coral cover on breakwaters and surrounding natural reefs in Naha Port from 1989 to 2018. Black lines indicate the 1998 coral bleaching event. (**b**) Non-metric multidimensional scaling (nMDS) plots of coral communities of breakwater and surrounding natural reefs, based on coral cover and other benthic cover from 1989 to 2018. Grey vectors show coral taxa and other benthic substrates that correlate mostly with the change of communities. (**c**) Standardized effect size of explanatory variables of additive log-ratio (alr)-transformed coral cover after the 1998 bleaching event, from a Bayesian hierarchical model. White circles represent the median, and thin and thick horizontal lines represent the 95% and 80% highest density intervals (HDIs), respectively. Blue lines indicate that the 80% HDI does not overlap with 0, suggesting a credible trend, and grey lines indicate that the 80% HDI overlaps with 0, suggesting a less credible trend. For the effect of substrate (i.e., breakwaters or natural reefs), a positive effect size indicates a relatively positive effect on the coral cover on natural reefs and a negative effect size indicates a relatively positive effect on the coral cover on breakwaters. Please refer to Eq. (1) for a detailed explanation.

We compared the coral cover on breakwaters and natural reefs in Naha Port from 1989 to 2018 (Fig. 2). The total areas surveyed for the breakwaters and natural reefs were 1.4 and 14.1 ha, respectively. Coral cover on the outer (offshore) side of the breakwaters declined to a mean of 3% in 2000 after the 1998 bleaching event, but by 2004, 6 years after the bleaching event, coral cover had autonomously recovered to a mean of 25%, equivalent to the pre-bleaching level (mean coral cover of 23% from 1989 to 1996). On the other hand, the average total coral cover on natural reefs around breakwaters was 45% before the 1998 bleaching event, but it dropped to 14% in 2000, after the event. The coral cover on natural reefs barely recovered from bleaching between 2000 and 2004. During the entire post-bleaching period (2000–2018), total hard coral cover was higher on the breakwaters than on the natural reefs (Fig. 2c). Total hard coral cover was higher at shallower depths and increased with years elapsed after bleaching. Total hard coral cover tended to be higher at shallower depths on the breakwaters than on the natural reefs, and the change in coral cover on the breakwaters was faster than on the natural reefs. By coral taxonomic group, the cover of *Acropora* was higher on the breakwaters than on the natural reefs, whereas the cover of Faviidae (although the traditional family Faviidae has now been absorbed into Merulinidae, we use Faviidae because it includes data that were only identified at the family level in surveys conducted prior to the change in taxonomy), *Porites*, and other hard corals was lower on the breakwaters than on the natural reefs. During the period up to the sixth year after bleaching (2000–2004), when coral cover on the breakwaters had recovered to pre-bleaching levels, the rate of change in total hard coral and cover of each taxon (i.e., the rate of recovery from bleaching) was faster on the breakwaters than on the natural reefs, especially for *Acropora*, and cover was higher at shallower depths for *Acropora*, *Montipora*, and Faviidae (Fig. 2). PERMANOVA results showed significant differences in community structure among depth, research year, the interactions between substrate and research year, and substrate and depth, with differences in substrate (i.e., artificial structure or natural reef) having the greatest effect on community structure (Supplementary Table S1). Non-metric multidimensional scaling (nMDS) results showed differences in the community trajectories of before versus after bleaching between artificial structures and natural reefs (Fig. 2b). On the breakwaters, the community was characterized mainly by *Acropora* and *Pocillopora* before bleaching, but in 2000 the community was characterized by non-coral substrates and differed from any of the pre-bleaching communities. After 2001, the community gradually shifted to be characterized by *Acropora* and *Pocillopora*, and in many years the community structure did not differ from that before bleaching. The community structure in 2004–2009 differed significantly from that in 2001 (Supplementary Fig. S1), with *Acropora* and *Pocillopora* becoming more represented in the community (Fig. 2a,b). On the other hand, the community structure on natural reefs differed significantly between pre- and post-bleaching periods in almost all cases (Supplementary Fig. S1), with communities characterized by *Acropora* before bleaching, but mainly by non-coral substrates, Faviidae, and other hard corals after bleaching (Fig. 2a,b). Community structure did not change during the post-bleaching period 2001–2006. After 2007, the community structure in many years differed significantly from that of earlier years, with Faviidae, and other hard corals becoming more abundant.

### Effects of interventions on coral reef ecosystems: surface processing of wave-dissipating blocks and creation of artificial tide pools

**Fig. 3:**
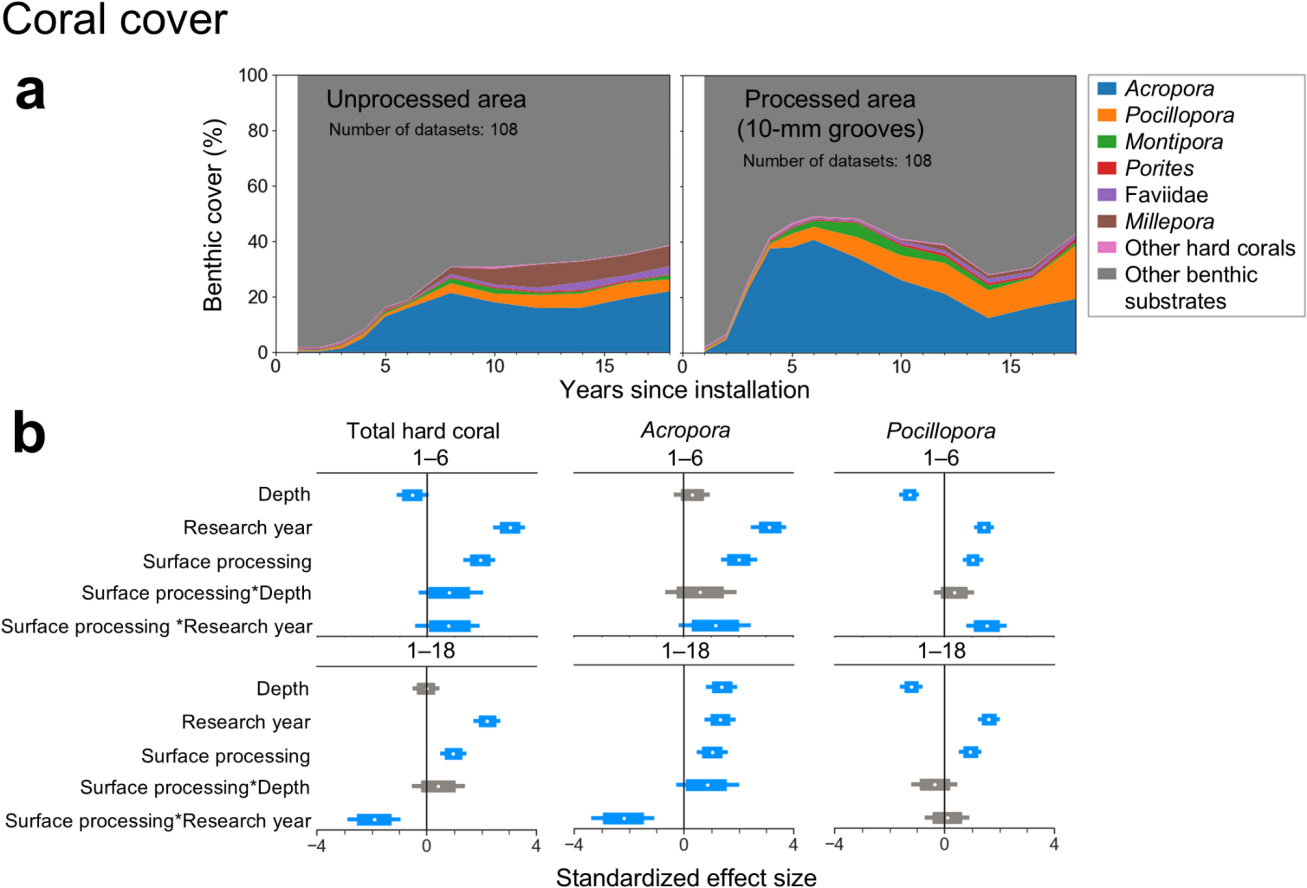
Effect of surface processing of wave-dissipating blocks on coral cover from 2000 to 2017. (**a**) Temporal dynamics in mean percent hard coral cover on unprocessed and processed areas. (**b**) Standardized effect size of explanatory variables of alr-transformed coral cover, from a Bayesian hierarchical model for the early (1–6 years after construction) and the entire (1–18 years after construction) periods. The explanation of the legends is the same as in Fig. 2c. For the effect of surface processing, a positive effect size indicates a relatively positive effect on the coral cover in processed areas and a negative effect size indicates a relatively positive effect on the coral cover in unprocessed areas. Please refer to Eq. (2) for a detailed explanation.

The mean total coral cover in the processed areas of wave-dissipating blocks with 10-mm grooves, installed on the Naha Breakwater in 1999, exceeded 20% three years after installation and reached 49% six years after installation. In contrast, the mean total coral cover in the unprocessed areas was 18% after 6 years, although the mean total coral cover was similar in the two types of area from 10 to 18 years after installation (Fig. 3a). Surface processing had a positive effect on total hard coral cover (Fig. 3b). Total hard coral cover increased at a faster rate in the processed areas than in the unprocessed areas in the early period (1–6 years after construction), although the rate of increase in coral cover during the entire period (1–18 years after construction) was lower in the processed areas than in the unprocessed areas. Surface processing also had a positive effect on the total number of coral colonies, although the rate of increase in the number of coral colonies during the entire period was lower in the processed areas than in the unprocessed areas. The number of coral colonies was similar in the two types of area from 8 to 18 years after installation (Supplementary Fig. S2). In terms of coral taxa, positive effects of surface processing on the number of coral colonies were found only for *Acropora* and *Pocillopora*, and the 80% HDI of the effect sizes of surface processing on coral cover during the early period for these taxa were higher than those for the other taxa (Fig. 3 and Supplementary Figs. S2 and S3). Both cover and abundance of *Acropora* in the processed areas were similar to those in the unprocessed areas from 8 to 18 years after installation.

**Fig. 4:**
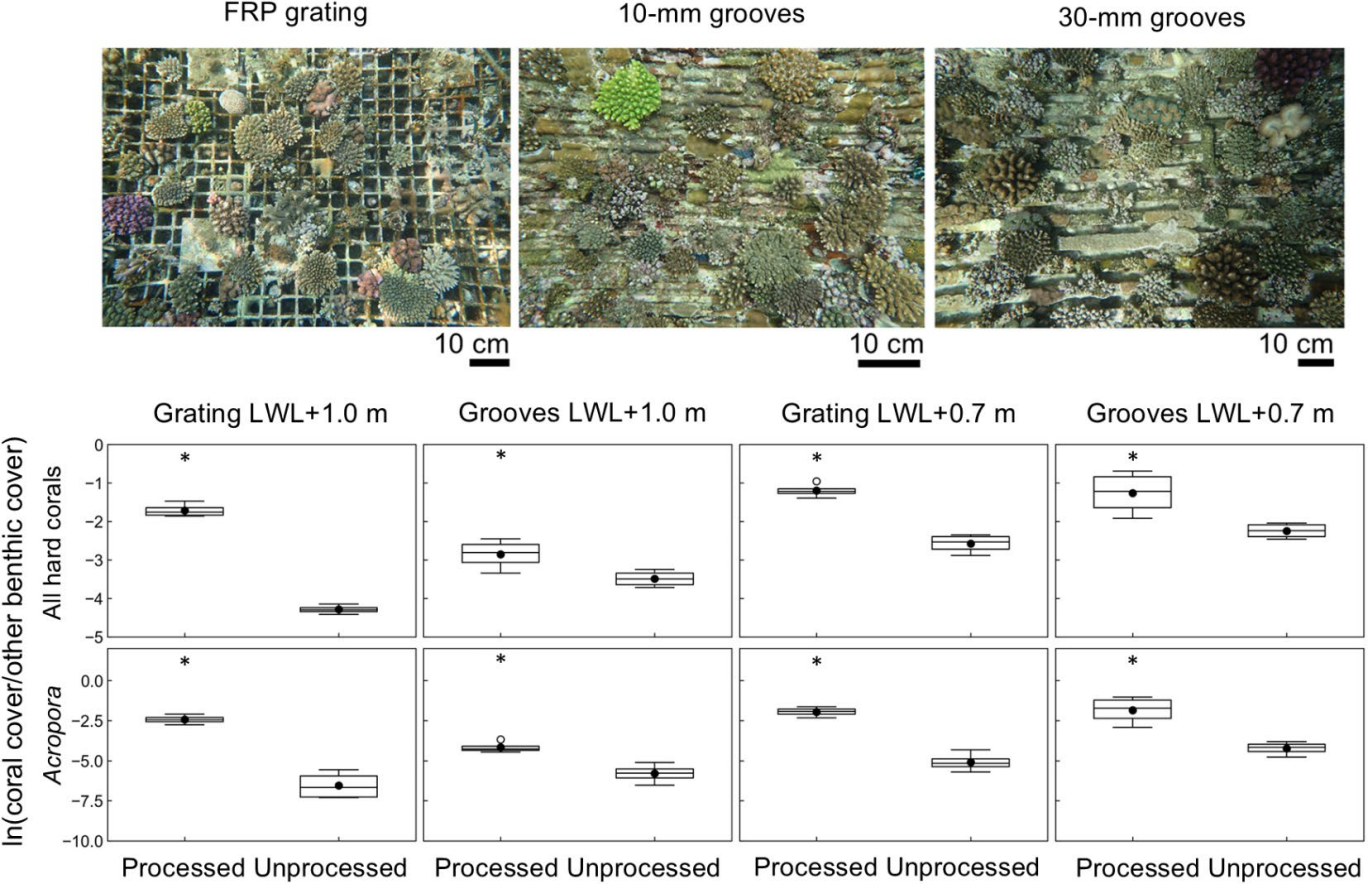
Coral community enhancement by surface processing of artificial tide pools (ATPs). The alr-transformed coral cover on the processed and unprocessed areas of the bottom of ATPs with FRP grating or grooves (10-mm or 30-mm) is shown for two installation depths: low water level (LWL) +1.0 m and +0.7 m. Boxes show the 25% and 75% quantiles; horizontal lines inside the boxes are median values; whiskers show maximum and minimum values; black circles are mean values; white circles are outliers. Where there is a significant difference between the processed and unprocessed areas (paired *t*-test or Wilcoxon signed rank test for alr-transformed coral cover; *P* < 0.05), the higher value is marked above with an asterisk. Photographs of surface-processed areas are taken from Tanaya et al.^31^.

Artificial tide pools (ATPs) are box-shaped structures installed on the inner (port) side of the First Urasoe Breakwater to promote coral colonization (Fig. 1a; Tanaya et al.^31^). The top edge of the walls of each ATP is either 1.0 m above low water level (LWL +1.0 m; stations [St.] 1, 2, and 3) or at LWL +0.7 m (St. 4, 5, and 6). There are three types of surfaces at the center of the inside bottom of each ATP: (1) 10-mm or 30-mm grooves (St. 2 and 5), (2) fiber reinforced plastic (FRP) grating (40-mm grid size, 8 cm thick; St. 1 and 4), or (3) no surface processing (St. 3 and 6) (Supplementary Fig. S4). In the ATPs at LWL +0.7 m, total coral cover averaged about 18% in the grooved areas (St. 5) and FRP-grating areas (St. 4), and *Acropora* cover was also higher in these areas (Supplementary Fig. S4). Surface processing had a positive effect on coral cover by taxon, community size, and total coral cover on the bottom of the ATPs (Fig. 4, Supplementary Fig. S5, Supplementary Tables S2‒S4). Total coral cover and *Acropora* cover were higher in the processed areas than in the unprocessed areas at all sites (Fig. 4, Supplementary Table S2). For *Millepora*, *Montipora*, *Porites*, and *Pocillopora*, coral cover was higher in the processed areas than in the unprocessed areas in some cases (Supplementary Table S2). The total number of coral colonies in ATPs was higher in the processed areas than in the unprocessed areas in ATPs with FRP grating or grooves at LWL +0.7 m (Supplementary Fig. S5, Supplementary Table S3). The number of *Acropora* colonies in the processed areas exceeded that in the unprocessed areas at all sites (Supplementary Fig. S2, Supplementary Table S3). For *Millepora*, *Porites*, and *Pocillopora*, the number of colonies in the processed areas was higher than that in the unprocessed areas in some cases (Supplementary Table S3). The increase in the size of coral colonies was more pronounced in the processed area with FRP grating than in the processed area with grooves (Supplementary Fig. S5, Supplementary Table S4). Although the mean size of all coral colonies together was higher not only in the processed areas with FRP grating but also in the processed areas with grooves, the mean sizes of colonies of *Acropora*, Faviidae, *Porites*, and *Pocillopora* were higher than in the unprocessed areas only in the case of the processed area with FRP grating (Supplementary Table S5).

Surface processing of ATPs also had positive effects on coral growth and fish density (Supplementary Fig. S6). The fish density observed in ATPs was highest in ATPs with FRP grating at LWL +0.7 m (Supplementary Fig. S6). The fish density was higher in ATPs with FRP grating than in unprocessed ATPs at both LWL +0.7 m and LWL +1.0 m (Supplementary Fig. S6). The net ecosystem calcification rate (NEC) during the daytime stagnant period was higher in the ATPs with FRP grating or grooves than in the unprocessed ATPs at both LWL +1.0 m and LWL +0.7 m except for the ATPs with grooves at LWL +1.0 m in August 30 (Supplementary Fig. S6). NEC was particularly high in the ATP with grooves at LWL +0.7 m (St. 5-3 [ATP #3 at St. 5]), where *Acropora* cover was highest (Supplementary Fig. S7). The daily NEC estimated from the regression equation of NEC against light intensity (Supplementary Fig. S8) ranged from 65 ± 6 mmol CaCO_3_ m^−2^ day^−1^ (St. 6-3, September 2019) to 163 ± 12 mmol CaCO_3_ m^−2^ day^− 1^ (St. 5-3, August 2019) (Supplementary Table S5).

### Optimal environmental conditions for coral growth

**Fig. 5:**
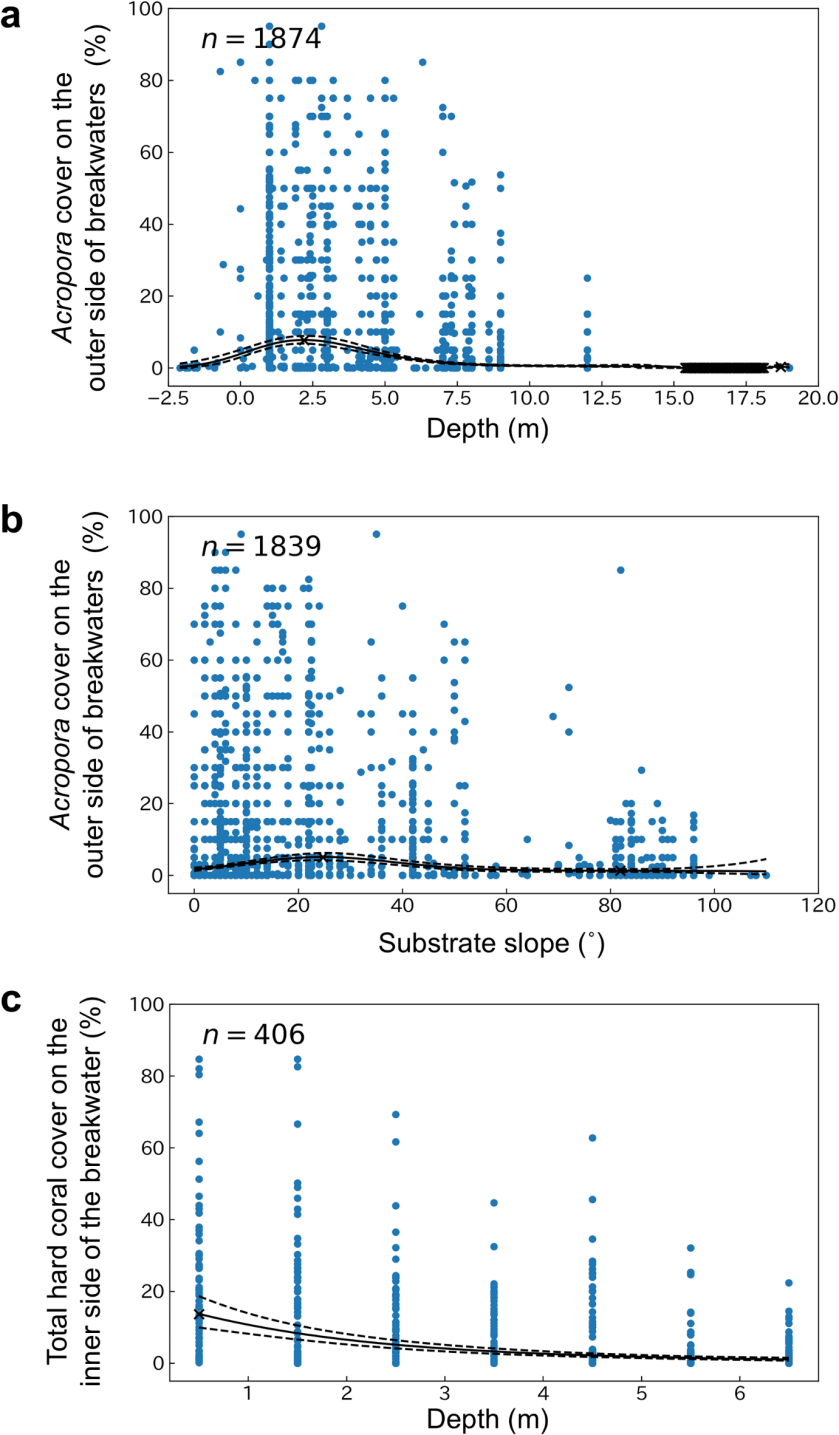
Coral cover of *Acropora* or total hard coral on breakwaters versus water depth or substrate slope. *Acropora* cover on the outer (offshore) side of breakwaters versus (**a**) water depth or (**b**) substrate slope. (**c**) Total coral cover versus water depth on the vertical walls on the inner (port) side of the breakwater. Solid black lines show the values estimated by the generalized additive model, and dashed lines show 95% confidence intervals. The smoothing coefficients and degrees of freedom of the generalized additive model were determined by generalized cross-validation. Each blue point represents a single measurement from a quadrat or a transect. Extreme values are indicated by cross marks.

**Fig. 6:**
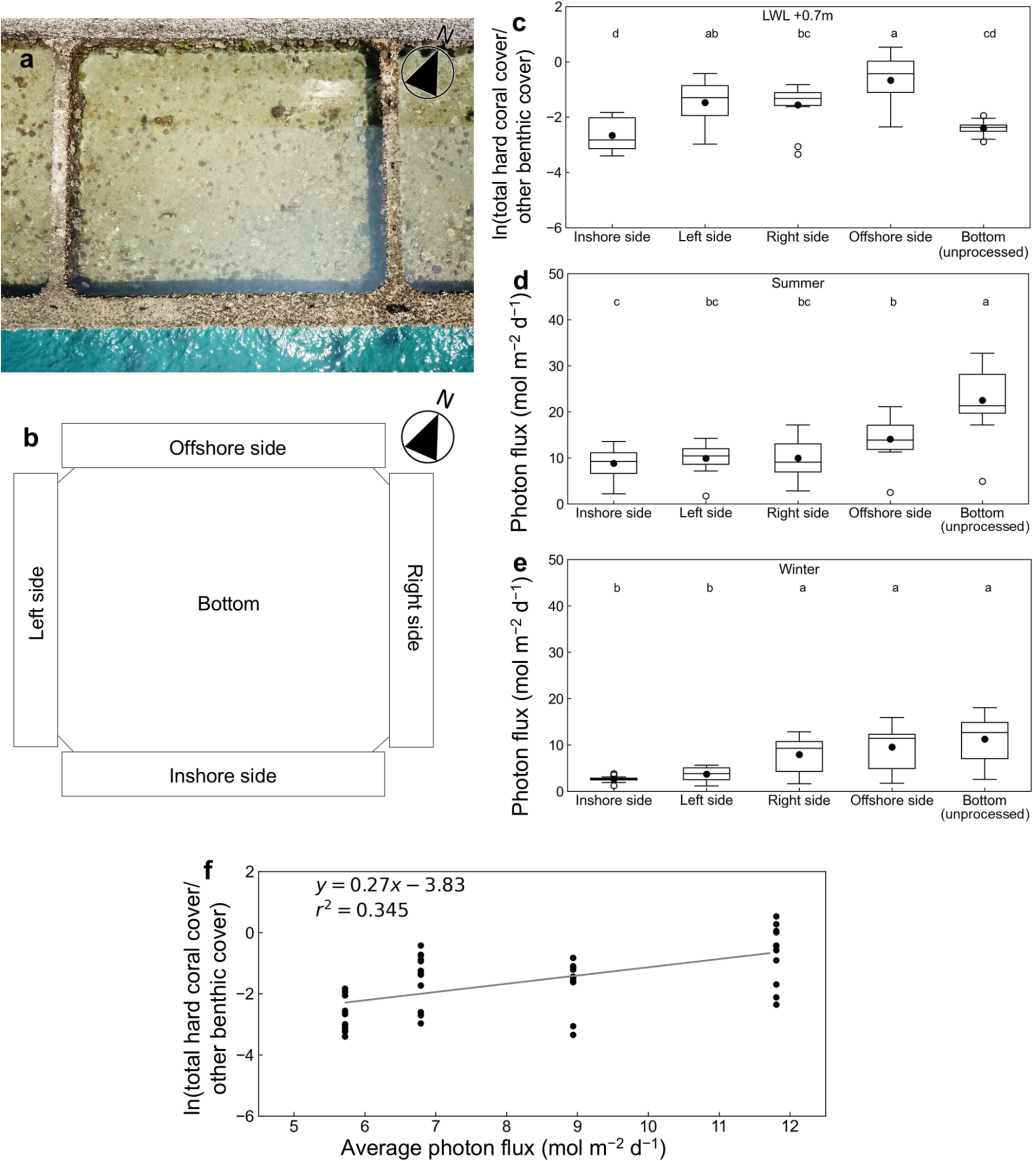
Artificial tide pool (ATP) layout, alr-transformed total coral cover, light flux in summer and winter, and alr-transformed total coral cover versus light flux. (**a**) Photograph of an ATP; (**b**) expanded plan view; (**c**) alr-transformed total hard coral cover on the five inner surfaces of ATPs; daily photon flux in (**d**) summer and (**e**) winter; and (**f**) alr-transformed hard coral cover on ATP side walls versus average daily photon flux. In (**c**–**e**), boxes show the 25% and 75% quantiles, horizontal lines inside the boxes are median values, whiskers show maximum and minimum values, black circles are mean values, and white circles are outliers. Letters (a–d) indicate significant differences among the inner surfaces (ANOVA, *P* < 0.05). The slope of the regression line in (**f**) is significant (*P* < 0.05, *r*^2^ = 0.345).

To determine the optimal environmental conditions for coral growth on artificial structures, we investigated the relationship between coral cover and environmental conditions (water depth, substrate slope, and light intensity) on artificial structures (breakwaters) in Naha Port (Figs. 5 and 6, Supplementary Figs. S9‒S11). The cover of *Acropora—*the most common coral genus on the outer (offshore) side of the breakwaters—was higher at shallow depths, with the highest cover of 7.8% (95% confidence interval, 6.8–8.9%) at LWL−2.2 m, as estimated by a generalized additive model (GAM), and then decreasing with increasing depth (Fig. 5). The substrate slope affected coral cover: *Acropora* cover on the outer side of the breakwater was highest (5.1%; 95% confidence interval, 4.2–6.2%) at a substrate slope of 25°, as estimated by GAM, and then decreased with increasing slope (i.e., as the substrate slope became closer to vertical) (Fig. 5; Supplementary Fig. S10). On the vertical wall of the inner (port) side of the breakwater, with a substrate slope of 90°, the cover of all taxa and the total coral cover were higher at shallower depths (Supplementary Fig. S9).

Total coral cover on the inside wall of the ATP placed at LWL +0.7 m was highest on the south-facing (offshore) side and lowest on the north-facing (inshore) side and untreated bottom (Fig. 6c). Of the coral taxa, only *Porites* had cover higher on the unprocessed bottom than on the inshore side (Supplementary Fig. S11). The light intensity on the ATP wall was higher on the offshore side and the bottom than on the inshore side in both summer and winter (Fig. 6d,e). For the four sides of the ATP, which have the same slope of 90°, the higher the light intensity (averaged over summer and winter) reaching the side, the higher the coral cover (Fig. 6f).

We compared the differences in light intensity, salinity, water temperature, and wave height inside and outside the port and by water depth (LWL −1 m and –3 m) for each month by using physical observations from the First Urasoe Breakwater for July 2018 to June 2019 (Supplementary Tables S6–S15). There were no significant differences in daily mean water temperatures for each month between the inner (inside the port) and outer (outside the port) sides of the breakwater at either depth (Supplementary Tables S12–S14). The daily mean light intensity at LWL −1 m on the inner side each month was higher than, or did not differ substantially from, that at LWL −3 m on the outer side of the breakwater, except in June 2019 (Supplementary Table S8). The mean significant wave height for each month was higher on the outer side of the breakwater than on the inner side (Supplementary Table S11).

Surface-water transparency (mean ± SD) did not differ significantly between the offshore side of the First Shinko Breakwater (10.4 ± 3.9 m, *n* = 190) and the offshore side of the Urasoe reef (11.2 ± 3.2 m, *n* = 91) (Supplementary Table S16). The *Acropora* coral cover was higher in areas with low sedimentation (sedimentation rank I [see Methods]) than in areas with high sedimentation (rank II) both on breakwaters and on natural reefs (Supplementary Table S17).

## Discussion

### Role of artificial structures as coral habitat after mass coral bleaching

On the breakwaters at Naha Port, the *Acropora*-dominated coral community recovered rapidly from mass bleaching in 1998, with recovery complete by about 6 years after the bleaching (Fig. 2). This recovery rate (cover increase of about 6% year^−1^ from 2000 to 2004) is comparable to that of healthy coral reefs: reef slope cover 9% in 1998 to 44% in 2010 (~ 3% year^−1^)^32^; reef flat cover, 3% in 2001 to 47% in 2010 (~ 5% year^−1^)^33^. On the other hand, the *Acropora* community on the natural reefs, which was dominant prior to bleaching, has not recovered since the 1998 bleaching event (Fig. 2). Although some corals, such as Faviidae and other hard corals, increased their coverage after bleaching, the coral community as a whole was different from that before the 1998 bleaching event. On the natural coral reefs along the coast of Okinawa Island, where Naha Port is located, *Acropora* cover has not recovered at many sites since the large-scale bleaching of 1998, suggesting a decline in the resilience of the coral community, probably due to terrestrial loading of sediment^34^. The results suggests that the coral community on the breakwater was highly resilient despite the decrease in cover due to disturbance. In Naha Port, artificial structures provided more favorable conditions for *Acropora* than did the natural reefs.

Although few studies have continuously monitored the development of coral communities on artificial structures beyond the initial stages of colonization^35^, our study shows that coral communities on artificial structures can be established and maintained for several decades, with coverage within a few years after construction comparable to that of surrounding natural reefs. In the case of *Acropora*, coral cover was higher than that of the surrounding natural reefs, whereas Faviidae, *Porites*, and other hard corals had lower cover than on the surrounding natural reefs, resulting in the formation of communities that differed from natural reefs (Fig. 2b,c). *Acropora*, the dominant taxon on artificial structures, is fast-growing and prefers shallow depths. *Acropora* is a highly competitive coral that reproduces by mass spawning and external fertilization; under ideal conditions, it can rapidly cover habitat^32,36–38^. We attribute the rapid recovery of coral cover after bleaching on the breakwaters of Naha Port to an increase in abundance of the highly competitive *Acropora*.

Artificial structures can serve as habitats for *Acropora*, which are important species for ecosystem functioning, and we expect that they will play an important role in maintaining *Acropora* communities. Maintenance of high population densities is important for mass spawning corals such as *Acropora* to increase the probability of successful sexual reproduction and create genetically diverse colonies^36,39^. Although *Acropora* has important ecological functions, such as attracting fish and building reef frameworks^40–42^, it is sensitive to disturbances such as typhoons and high temperatures^7,33,43^. Healthy coral communities, even on a small scale, play important roles in supplying larvae to other locations and as a source of genes for adaptation^44^. The results of this study empirically demonstrate that artificial structures installed in ideal environmental conditions for corals can function effectively as coral habitats, even when disturbed by high water temperatures. It should be noted, however, that artificial structures do not completely replace the functions of natural coral reefs. For example, the relative scarcity of *Porites* and Faviidae on artificial structures, which may be partly influenced by the time period of this study (years recorded since construction of breakwaters: 0–38 years) and by the slow recruitment and growth of these taxa, suggests that they do not provide optimal habitat for species of polychaetes^45^, crustaceans^46^, and fishes^47^ that depend on these corals. Coral communities on artificial structures may be more susceptible to environmental change because of the lack of stress-tolerant corals such as *Porites* and Faviidae^36^. However, if artificial structures maintain a suitable environment for corals, highly competitive *Acropora* will recover after disturbances and help maintain coral communities in these environments.

### Environmental conditions for rapid recovery of coral communities

For artificial structures to function effectively as coral habitats, it is extremely important that they are installed under environmental conditions suitable for coral growth. In the case of the breakwaters, *Acropora* cover was higher in shallower water, at lower substrate slopes, and on the offshore side of the breakwaters with less sedimentation (Fig. 5, Supplementary Table S17). Water depth is a determinant of physical conditions such as light intensity and wave-driven currents, and light intensity and wave-driven flows decrease with increasing water depth. *Acropora* cover on the outer side of the breakwaters peaks at LWL −2.2 m and decreases with depth below this (Fig. 5), suggesting that shallower areas with higher light levels and faster wave-driven currents are suitable for *Acropora* growth. The suitability of areas with high light levels for *Acropora* growth is also suggested by the observation that *Acropora* cover was higher on the outer side of the breakwater, where the slope of the substrate was less (Fig. 5). On this side of the breakwater, as the slope of the substrate increases, the amount of light reaching the substrate and the growth rate of the coral community decrease^27^. (Note, however, that on the inner side of the breakwater, which is more susceptible to sedimentation, coral cover is higher on vertical walls with high slope than on horizontal surfaces [Fig. 6].)

Water flow plays an important role in coral growth and survival by reducing sedimentation, promoting coral metabolism, increasing the uptake of plankton and other food sources, and reducing bleaching^48–52^. Sediment interferes with coral larval recruitment and the growth and survival of juvenile corals^53^. It is particularly detrimental to the growth of less sediment-tolerant corals with small polyps, thin tissue, and flat colonies, such as tabular, corymbose, and encrusting *Acropora*, which are abundant on the outer side of breakwaters^51^. We attribute the lower *Acropora* cover in the shallow area (LWL −1 m) on the inner side of the breakwaters compared with the shallow area (LWL −3 m) on the outer side, even with no difference in light intensity (Supplementary Table S8), to less wave exposure than on the outer side of the breakwater (Supplementary Table S11). Although there was no difference in the transparency of the surface seawater between inside and outside the breakwater (Supplementary Table S16), sedimentation is likely to be greater inside the port, which is sheltered from the waves coming from offshore, than on the wave-exposed areas outside the port. In fact, 30–50 kg m^−3^ of sediment has been observed in the algal mat formed on the wave-dissipating blocks^54^, and this is considered to represent a substantial amount of sediment, which can negatively affect coral growth^55^.

Further investigation is needed to determine the factors behind the difference in coral distribution between natural coral reefs and artificial structures. Water temperature and transparency, which affect coral growth, did not clearly differ between breakwaters and natural reefs. Around the coral distribution survey sites, the difference in mean water temperature of the surface layer (approximately LWL −2 m to LWL −3.5 m) for each month between artificial structures and natural reefs was less than 0.7 °C (Supplementary Fig. S12). There is no significant difference in the transparency of surface seawater between the offshore side of the First Shinko Breakwater and the offshore side of the Urasoe reef (Supplementary Table S16), suggesting that there was no difference between them in light conditions or turbidity. It is possible, however, that there is a difference in the flow structure between natural reefs and artificial structures. Despite there being no difference in water transparency between artificial structures and natural reefs, the higher *Acropora* cover of artificial structures to greater depths than on natural reefs (Supplementary Fig. S9) suggests that wave-driven flows are stronger on artificial structures. The distribution of corals on breakwaters differs depending on the surrounding flow and conditions, such as the offshore wave height and wave exposure^56^. In the case of the breakwaters in Naha Port, the First Urasoe Breakwater is located offshore from the natural coral reefs, and this may result in stronger wave exposure than on the natural reef and provide more suitable conditions for coral growth. In addition, the structure formed by the wave-dissipating blocks on the outer side of the breakwaters differs from that on natural coral reefs in terms of the slope of the stacked blocks from the seabed, the gaps between the blocks that allow water to pass through, and the roughness, at scales from the surface of the blocks to the entire wave-dissipating structure.

Another possible reason for the lower coverage of Faviidae, *Porites*, and other hard corals on the breakwaters than on natural reefs (Fig. 2c) is the younger age of the substrate of the breakwaters compared with the surrounding natural reefs; this may limit the growth of corals with slow growth or low recruitment. The maximum diameters of *Pocillopora*, Faviidae, and *Porites* colonies were significantly greater on natural reefs than on the breakwaters, with no significant differences for the other taxa (Supplementary Table S18). In particular, the mean maximum diameters of the Faviidae and *Porites* colonies were triple and double those on artificial structures, respectively. Because Faviidae and *Porites* are relatively slow-growing corals, the smaller size on the breakwaters may be due to the younger substrate. A previous study reported that the cover of a certain species of *Porites* on artificial structures that were more than 25 years old did not differ from that on the surrounding natural reefs^21^. The distributions of benthic organisms that compete with corals for space, and of coral predators, may differ if the environmental conditions and the age of the substrate are different. However, coral recruitment is not likely to differ between concrete and natural coral reef rock when the environmental conditions are the same^20^. A comparison of data on the maximum coral diameters recorded on breakwaters and natural reefs during 2000–2004 showed no strong effect of substrate type (Supplementary Fig. S13). In contrast, substrate type had a strong effect on the number of *Acropora* colonies during 2000–2004, and the number of *Acropora* colonies tended to be higher on breakwaters than natural reefs. The data set used in this study did not include enough records of community abundance and size, especially on natural reefs, to fully examine the effects of different substrates on coral communities, taking into account differences in location and environmental factors. However, our finding that *Acropora* abundance tended to be higher on breakwaters, together with the small mean maximum colony diameter in 2000 (5.1 cm), two years after bleaching, suggests that post-bleaching *Acropora* recruitment, survival of juvenile colonies, or both may have been higher on the breakwaters than on natural reefs. The physical attributes of the substrate surface, such as difficulty of attachment^57,58^; the distribution of sediments that prevent attachment^59,60^; and the distribution of algae, invertebrates, and other organisms that affect larval attachment, metamorphosis, and survival may differ between natural coral reefs and breakwaters^61^.

### Creation of artificial habitats for mitigating coral losses

The coral cover and the number of colonies in processed areas of the wave-dissipating blocks were higher than those in the unprocessed areas in the early period (1–6 years after construction) (Fig. 3 and Supplementary Fig. S2). Previous studies have reported that the grooved surfaces on blocks have a positive effect on coral settlement, at least up to the third year after construction^29,30^. Our results indicate that adding grooves to the surface of the wave-dissipating blocks installed on the outer side of the breakwaters in the early stages of construction effectively promotes the settlement of coral larvae and/or the survival of juvenile corals, as well as the associated increase in coral cover. Species-specific analysis showed that this method was effective in promoting colonization by the broadcast-spawning *Acropora* and *Pocillopora*. In addition, a rapid increase in the number of colonies in the second and third years after construction (11‒48 colonies m^−2^ y^−1^ on average; Supplementary Fig. S2) was accompanied by a very rapid increase in coral cover in the third and fourth years after construction (16‒20% y^−1^ on average; Fig. 3). This suggests that it is important to promote the settlement of juvenile corals to rapidly increase coral cover.

Although the long-term effect of surface processing of the wave-dissipating blocks on the outer sides of the breakwaters on coral cover and population density was smaller than the effect in the early period, surface processing may be useful for promoting rapid recovery of the coral community, considering the increasing frequency of disturbances of the coral community due to higher water temperatures with climate change^28^. The increase in population density may be useful for promoting early recovery of coral communities. Increasing population densities may increase reproductive success and genetic diversity^36^, and higher quality larvae are produced more abundantly in coral populations with higher cover^62^. The creation of high-cover coral communities, even if localized, can enhance the recovery of coral communities over a broader area^44^.

Our finding that coral cover increased more rapidly in the processed areas than in the unprocessed areas is consistent with the fact that coral communities on natural reefs recover more quickly from disturbance in areas of high structural complexity^63^. The roughness of the substrate surface reduces shear stress near the surface and may physically increase the probability of larval recruitment^57^. The grooves in processed areas may also inhibit feeding on young corals by herbivorous fishes. In natural coral reefs, juvenile corals are often found in small crevices, suggesting that crevices provide an effective refuge from herbivorous fish^64^.

In the ATPs installed at LWL +0.7 m, the average total coral cover in the processed areas with grooves (St. 5) or FRP grating (St. 4) reached about 18% within 5 years after construction (Supplementary Fig. S4). At depths of around LWL +0.1 m to +1.0 m, coral colonization is usually restricted because of exposure to air. However, by creating a tidal pool structure at these depths, such as an ATP, it is possible to increase the amount of substrate available for coral colonization, even at low tide, by around 6 m^2^ per meter of breakwater length. On the basis of the coral cover at St. 4, the increase in coral coverage due to the installation of the ATPs is around 0.95 m^2^ per meter of breakwater length, and 1.7 times the coral area on the port side of the breakwater where ATPs are absent^31^. The cost-effectiveness of installing ATPs is comparable to that of coral habitat restoration by transplantation in developed countries^31^.

One of the reasons why an ATP results in high coral accretion is that ATPs are installed in shallow water while mitigating the effects of air exposure. Coral cover was higher at shallower depths on the vertical wall of the inner (port) side of the breakwaters (Fig. 5). Furthermore, coral cover increased with the average light intensity reaching the vertical walls of the ATPs (Fig. 6), suggesting that coral growth was promoted by ensuring sufficient light intensity for photosynthesis by the zooxanthellae that coexist with the coral.

Coral cover was higher in areas processed with grooves or gratings (ATPs) than in unprocessed areas, especially for *Acropora*. Surface processing of the ATP bottom was found to be important in enhancing the coral colonization effect of the ATPs (Fig. 4, Supplementary Tables S2‒S4). The number of coral colonies per unit area was higher in the processed area than in the adjacent unprocessed area, suggesting that one of the effects of surface processing is the promotion of settlement of coral larvae and/or survival of juvenile corals and, as a result, an increase in coral cover, as also observed with the addition of grooves to wave-dissipating blocks on the outer side of the breakwaters. Grating also reportedly inhibits fish grazing and increases the initial survival rate of juvenile corals^65^. In addition to the increased coral cover and number of colonies in surface-processed areas, the coral communities were larger than in the unprocessed areas, suggesting that ATP surface processing is effective not only for encouraging the settlement of coral larvae and the initial survival of young corals, but also for long-term coral survival and growth.

On the inner, port side of the breakwater where the ATPs were installed, sedimentation is likely to be greater than on the wave-exposed outer side. Although the amount of light reaching the surface of the unprocessed ATP bottom was the same as, or greater than, that reaching the south-facing (offshore side) inside vertical wall of the ATP, the coral cover was lower than on the south-facing side (Fig. 6), suggesting that sedimentation may have inhibited coral settlement and growth. Only *Porites* spp., which are relatively tolerant of sedimentation, showed higher coverage on the bottom than on the inshore side (Supplementary Fig. S11), suggesting that sedimentation may inhibit the recruitment and growth of other coral species on the ATP bottom. Processing of ATP surfaces, such as adding grooves or grating, is effective in reducing the negative effects of sedimentation on corals by raising some of the substrate (by about 8 cm) above the bottom. In particular, the number of colonies, coral cover, and mean colony size increased in areas with grating at both ATP depths (Fig. 4, Supplementary Fig. S5, Supplementary Tables S2–S4), suggesting a positive effect of raising some of the substrate above the bottom, where sediment would first accumulate.

In addition to increasing the coral cover, ATP surface processing also increased the calcification rate of the coral community and the size of fish populations. The number of fish was particularly high in the ATP with FRP grating (Supplementary Fig. S6), suggesting that the three-dimensional shape of corals such as *Acropora* distributed in the FRP grating and surface-processed areas increased the structural complexity. Sheltered places for fish are more abundant in sites with high structural complexity, leading to increases in fish population size^63,66^. The net ecosystem calcification rate (NEC) was likely higher in the ATPs with surface-processed bottoms because of the more abundant *Acropora* (Supplementary Fig. S4), which has a high calcification rate^67^. The daily NEC of ATPs with FRP grating at LWL +0.7 m during the observation period was estimated at 121 ± 10 to 143 ± 9 mmol CaCO_3_ m^−2^ day^−1^ (Supplementary Table S5). This is as high as the NEC of a natural reef flat (mean 130, range 20‒250 mmol CaCO_3_ m^−2^ day^−1^)^68^. In addition, the prototype for the ATPs, a top-shell-snail aquaculture structure^69^, showed more coral larval recruitment than the surrounding natural reefs outside the pool structures^70^. The retention of water in the ATP during low tide likely traps coral larvae and promotes settlement, but further research is needed to demonstrate this possibility.

### Use of artificial structures to restore coral reef ecosystems in response to climate change

The coral community on the breakwaters in Naha Port recovered to pre-bleaching levels after about 6 years, reaching a total cover of about 20%. Although the coral community on the breakwaters is not a replica of the natural coral community, *Acropora*, which forms a complex three-dimensional skeleton, provides much of the cover (Fig. 2c), and the coral community in the ATPs on the First Urasoe Breakwater has a carbonate productivity comparable to that of natural coral reef ecosystems (Supplementary Fig. S6). Since the 2000s, large-scale coral bleaching has occurred on a 6-year cycle^28^, and the coral community on the artificial structures in Naha Port may be able to cope with such repeated bleaching. Although the bleaching cycle is expected to become shorter as global warming progresses, it is possible to increase the growth rate of coral cover on artificial structures in the first few years after construction by processing the surfaces of the artificial structures and ATPs (Figs. 2 and 3). Although efforts to mitigate global warming are essential for the restoration of coral reef ecosystems^4,9^, as global warming progresses and coral community mortality due to large-scale bleaching becomes more severe^2^ it is important to ensure that even small coral reefs remain healthy. Healthy coral communities, even on a small scale, are important as sources of coral larvae for other areas and as sources of genes for adaptation to higher water temperatures^44^.

Table 1 summarizes the layout and structural considerations suggested by this study for artificial structures to enhance the habitat function of corals (especially *Acropora*). To increase the growth rate of coral communities, we consider it effective to install structures in areas with moderate waves and currents, to provide water channels through the structures, to increase the shallow area and effective slope of the cross-sectional shape, and to include surface processing on surfaces in locations with favorable environments for coral colonization. Table 2 summarizes the challenges of restoring coral habitats by using artificial structures, and recommendations from this study, focusing on three criteria—efficiency, cost, and scalability—presented by Hein et al.^5^.

**Table 1:** Environmental conditions beneficial for coral growth (especially *Acropora* spp.) on artificial structures.

Category	Environmental conditions	Justification	Possible reasons
Planar layout	⋅External forces (currents, waves) suitable for coral growth ⋅Structural layout to promote water flow	⋅Corals grow well in areas with moderate waves and/or currents (higher wave energy, near water channels)	⋅Water flow promotes coral growth by ensuring adequate food and oxygen and preventing sedimentation
Cross-sectional shape	⋅Expansion of shallow-water area (e.g. artificial tide pools, structural units on a gentle slope) ⋅Increased effective slope areas (e.g. installation of wave-dissipating blocks in front of offshore vertical walls)	⋅Coral cover is higher at shallow depths, high light levels, and substrate slopes of around 25°	⋅Higher irradiance and water flow may promote coral growth
Surface processing	⋅Addition of relief (grooves or FRP grating) to substrate surfaces	⋅Surface texture increases larval settlement and/or initial survival, and increases initial growth of the coral community ⋅In areas with sedimentation, this can help long-term coral survival and growth	⋅Substrate roughness physically promotes larval settlement and provides shelter for juvenile corals ⋅Raising the substrate above the bottom reduces negative effects of sedimentation

**Table 2:** Challenges of coral habitat restoration using artificial structures, and recommendations from this study. Letters (a–c) highlight the relevant outcomes from this study.

Criteria coming from Hein et al.^5^	Challenges suggested by this and previous studies	Implications from this study
Efficiency	⋅Limited evidence of success linked to structures being overgrown by corals^5^ (a)	⋅Coral communities on artificial structures are maintained for several decades, with coverage comparable to or higher than that of surrounding natural reefs (a) ⋅Rapid establishment or recovery of coral communities after disturbance, within a few years after construction under suitable environmental conditions (a) ⋅Coral communities on artificial structures are different from those in surrounding natural reefs, but some corals lost from surrounding natural reefs by disturbance (*Acropora* spp. in this study) can be restored on artificial structures (a)
	⋅Low species diversity likely due to low complexity or the young age of substrate^18,21^	⋅Coral growth and fish communities can be enhanced by habitat engineering of planar layout (wave exposure, orientation), cross-sectional shape (depth, slope), and surface processing
	⋅Slow-growing corals are smaller than on natural reefs, probably due to the relatively young substrate (this study).	
	⋅Negative impact of structures on the surrounding environment and aesthetics^5^	
Cost	⋅Expensive to design and deploy^5^ (b)	⋅Cost-effectiveness can be improved by using existing artificial structures for coral-reef restoration and habitat engineering (e.g. adding artificial tide pools to breakwaters) (b)
Scalability	⋅Limited to small scale projects^5^ (c)	⋅Structures like breakwaters can create new habitat on a scale of several to several dozen hectares (c)

By using a large number of field observation data on the coral distribution on breakwaters and surrounding natural reefs for the past 29 years, we successfully demonstrated the effectiveness of artificial structures as sites for restoring coral habitat. Although habitat creation by using artificial structures does not regenerate the same natural coral reef ecosystem (e.g., species diversity different from that of natural coral reefs; accretion of slow-growing corals), such environmental interventions can result in the restoration of coral communities lost from natural reefs due to disturbance, or an early recovery of coral communities from disturbances.

The use of marine structures like breakwaters could also address the cost and scalability issues associated with artificial-structure-based coral restoration methods. Artificial structures such as breakwaters, harbors, energy platforms such as those for offshore wind turbines and oil and natural gas production facilities, and wrecks, are all constructed for purposes other than ecosystem restoration, and their function as biological habitats is often recognized only as a secondary value until the structures are removed^71^. However, it is possible to utilize the shallow depth zones suitable for coral growth on existing infrastructure at low additional cost. For example, the cost-effectiveness of restoring coral communities in ATPs by utilizing the shallow areas of breakwaters is comparable to that of transplantation in developed countries^31^. The use of artificial structures also helps to increase the scale of coral habitat restoration. The scale of restoration by transplantation, a common method for restoring coral reef ecosystems, is 500 m^2^ (median value^15^), with a maximum size of 7 ha^72^. In the case of the First Urasoe Breakwater, built on the seabed at LWL − 20 m, the surface area of new infrastructure created by the pro-environment breakwater is about 100 m^2^ per meter of breakwater length^31^. For a breakwater extending over several hundred to several thousand meters, the area would be several to several tens of hectares. If we consider this infrastructure as new habitat for corals and other marine organisms, then improving its habitat functions would be extremely important for the restoration of coral reef ecosystems in the surrounding areas.

## Methods

### Comparison of coral distribution on breakwaters and surrounding natural reefs before and after the 1998 mass coral bleaching event

We obtained historical data on coral distributions from survey reports on corals at Naha Port conducted by the Okinawa General Bureau. In this area, coral distributions on artificial structures (outer [offshore] side of the breakwaters) and the surrounding natural coral reefs have been recorded from fiscal year 1989 to fiscal year 2018. The coral cover was recorded by one of the following methods: the quadrat method, in which a quadrat is placed at a fixed point and the coral cover in the quadrat is recorded; the belt transect method, in which coral cover is recorded at regular intervals along a transect line on both sides of the measuring line; the spot check method, in which coral cover is recorded by swimming around a survey point (approximately 10 m in radius); the manta method, in which coral cover within the survey area is recorded by towing a survey crew by ship; and the snorkel method, in which coral cover is recorded by snorkelers. The numbers of data from each survey method in each year are listed in Supplementary Table S19. Total hard coral cover is the total coral cover excluding soft corals (%). In organizing the data on total coral cover and coral cover by taxon, we used the median value when cover was recorded in a certain range of values. A clear distinction was made between cases where no coral was found in the surveyed area (i.e., 0% cover) and cases where no survey was conducted (i.e., no data). For total coral cover in the survey, “R” (rare, for less than 1% cover) was treated as 0.5% cover, and “+” (for 1–5% cover) was treated as 3% cover. For the cover of each coral taxon (*Acropora* spp., *Pocillopora* spp., *Montipora* spp., *Porites* spp., Faviidae, *Millepora* spp., and other hard corals) with entries of “R” or “+”, the number of Rs was first counted (with “+” treated as 6 Rs). Because there was some range in the cover recorded by “R” and “+”, the sum of the cover by each taxon including them did not always match the value recorded as the total cover. Then, if the difference between the total coverage and the total coverage as the sum of the cover by each taxon recorded by real numbers was greater than zero, the difference divided by the number of R entries was used as the conversion value of 1 R. If the difference was less than zero, then the value of R was set to 0. To understand the trend of changes in coral distribution on artificial structures and surrounding natural reefs before and after the large-scale bleaching event in 1998, we calculated the mean of all data for coral cover (total cover and cover by taxon) in each survey year for breakwaters and natural reefs (Fig. 2a). The survey sites and the number of data points for each survey year are shown in Supplementary Tables S20 and S21.

A hierarchical Bayesian model was used to analyze whether post-bleaching coral cover and the recovery rate of coral cover from bleaching differed between breakwaters and natural coral reefs, taking into account differences in research sites and methods. To quantify the effect of substrate and other environmental factors on hard corals of each taxon, we modelled the total hard coral cover and cover of each taxon as separate responses. We removed the constant sum constraint by using the additive log-ratio (alr) transformation^73^, in which the total hard coral cover and the cover of each taxon (proportional data) are divided by the cover of the coral-free base (other benthic) and then taken as the logarithm, allowing the proportional data to be treated independently. To perform the alr transformation, we used a dataset with total coral cover and the cover of all taxonomic groups recorded. A hierarchical Bayesian model was constructed by using the attributes commonly obtained from historical data on breakwaters and natural reefs (substrate type, survey year, water depth) and their interactions as explanatory variables, with survey site and survey method as random effects. Among all covariates, the variation inflation factors (VIFs) are < 1.07 and the absolute values of Pearson’s correlation coefficients are < 0.25, indicating that multicollinearity is not serious (Supplementary Fig. S14; Supplementary Table S22).

The hierarchical Bayesian model takes the form of

1 $$\begin{aligned} y_{{cover}} \left( i \right){\text{ }} & = {\text{ }}\beta _{1} + {\text{ }}\beta _{2} f_{{substrate}} \left( i \right)_{{}} + {\text{ }}\beta _{3} X_{{depth}} \left( i \right){\text{ }} + {\text{ }}\beta _{4} X_{{year}} \left( i \right)_{{}} + {\text{ }}\beta _{5} f_{{substrate}} \left( i \right)_{{}} \\ & \quad *{\text{ }}X_{{depth}} \left( i \right){\text{ }} + {\text{ }}\beta _{6} f_{{substrate}} \left( i \right)_{{}} *~~~~~X_{{year}} \left( i \right) + {\text{ }}r_{{site}} \left[ {site\left( i \right)} \right]{\text{ }} + {\text{ }}r_{{method}} \left[ {method\left( i \right)} \right] \\ \end{aligned}$$

Here, *y*_*cover*_*(i)* is the alr-transformed coral cover to other benthic cover of record *i*, *f*_*substrate*_ is a categorical variable indicating the type of substrate (i.e., artificial structure or natural reef), with *f*_*substrate*_ = 0 for artificial structures and *f*_*substrate*_ = 1 for natural reefs, *X*_*depth*_ is water depth relative to LWL (m), *X*_*year*_ is the survey year (y), *f*_*substrate*_ * X_*depth*_ is the interaction between substrate type and water depth, *f*_*substrate*_ * X_*year*_ is the interaction between substrate type and survey year, *r*_*site*_*[site(i)]* is the effect of the survey site of record *i* (one of 11 survey sites), *r*_*method*_*[method(i)]* is the effect of the survey method of record *i* (one of the five survey methods), *β*_*1*_ is an intercept, and *β*_*2*_‒*β*_*6*_ are the coefficients of each explanatory variable, respectively. *y*_*cover*_*(i)* follows a normal distribution with mean *µ* and variance *ε*^2^ and the link function is set to identity. *r*_*site*_*[site(i)]* follows a normal distribution with mean 0 and variance *s*_*site*_^2^. *r*_*method*_*[method(i)]* follows a normal distribution with mean 0 and variance *s*_*method*_^2^. The non-informative prior distribution for *ε*, *s*_*site*_, and *s*_*method*_ is a Half-Cauchy distribution with a scale parameter of 10^−2^, and the non-informative prior distribution for *β*_*1*_‒*β*_*6*_ is a uniform distribution with lower and upper limits of −10 and 10, respectively. After standardizing the explanatory variables other than the survey sites and survey methods (by subtracting the mean and dividing by 2SD), the posterior probability distribution of the standardized coefficient (effect size) for each explanatory variable was estimated by Markov chain Monte Carlo (MCMC) sampling. The model was applied to data for the entire post-bleaching period (2000‒2018) for each coral taxon to test whether the coral cover differed between breakwaters and natural coral reefs after bleaching (i.e., the 80% HDI of coefficient *β*_*2*_ of *f*_*substrate*_ does not overlap with 0). The model was also applied to available data up to the sixth year after bleaching (2000‒2004), when coral cover on breakwaters recovered to pre-bleaching levels, to test whether the recovery rate of coral cover after bleaching differed depending on the substrate type (i.e., the 80% credible interval of coefficient *β*_*6*_ of *f*_*substrate*_ * X_*year*_ does not overlap with 0). MCMC sampling was performed by using “pymc3”^74^. We used the No-U-Turn sampler method^75^, where the first 1000 samplings were discarded as a burn-in (tuning) period and the following 2000 samplings were treated as the valid sampling period. The posterior probability distribution of *β*_*1*_‒*β*_*6*_ was estimated from a total of 10,000 sampling results obtained by performing this procedure five times. The Gelman-Rubin statistics were used to assess whether the MCMC sampling results converged to steady states (Supplementary Table S23). Model fits were assessed with Bayesian *R*^2^ (Supplementary Table S24). The SD of the model prediction (*ε*) and random effects (*s*_*site*_, and *s*_*method*_) are shown in Supplementary Table S25.

To visualize the temporal dynamics of coral communities of artificial structures and natural coral reefs, we used the vegan package in R to calculate the mean of dissimilarity matrices by using robust Aitchison distance for coral cover of each taxon and other benthic cover, and the non-metric multidimensional scaling method (nMDS) was conducted. Coral cover of each taxon and other benthic cover were transformed according to the method of Martín-Fernández et al.^76^ to address the zero-value problem. If the coverage is 0, it is replaced by half of the maximum rounding error of coral cover, 0.5% (0.0025). To visualize which coral taxa were related to difference in community structure, correlation vectors were overlaid on nMDS plots by using the envfit function. To examine the effects of substrate (artificial structure or natural reef), research year, and depth on coral community structure, a two-way PERMANOVA was performed by using the adonis2 function. Because the PERMANOVA results showed a significant main effect of substrate and survey year, a post-hoc test (pairwise PERMANOVA) was conducted to determine which pairs of survey years differed significantly in community structure for each of the artificial structures and natural reefs. We used 999 permutations for nMDS ordination and 9999 for PERMANOVA and pairwise PERMANOVA. The p-values for the pairwise PERMANOVA were corrected by using the Bonferroni method.

The maximum diameter of coral assemblages for each taxon was recorded as the largest assemblage appearing in the survey frame. The maximum diameters for each taxon were compared between natural reefs and breakwaters. The normality and equivariance of the data for each group were checked and the differences between the groups were compared by using Student’s *t*-test (Supplementary Table S18).

### Coral distribution survey on wave-dissipating blocks with surface processing

To investigate the effect of surface processing on wave-dissipating blocks, we compared the coral cover and number of coral colonies between processed areas with 10-mm grooves and unprocessed areas of the wave-dissipating blocks installed at Naha Breakwater in 1999. Coral cover and number of coral colonies in one 50 × 50 cm quadrat were recorded at each of three depths (LWL −2 m, −5 m, and −8 m) on each of three processed and three unprocessed section (18 total quadrats).

A hierarchical Bayesian model was used to analyze the effect of surface processing on coral cover and the number of coral colonies. A hierarchical Bayesian model was constructed by using the attributes (surface processing, years since installation, water depth) and their interactions as explanatory variables. Among all covariates, the variation inflation factors (VIFs) are 1.00 and the absolute value of Pearson’s correlation coefficients is < 0.08, indicating that multicollinearity is not serious.

The hierarchical Bayesian models take the form of

2 $$\begin{array}{ccccc}{y_{cover}}\left( i \right){\rm{ }} & = {\rm{ }}{\beta _7} + {\rm{ }}{\beta _8}{f_{surface{\rm{ }}processing}}{\left( i \right)_{}}\\& \quad + {\rm{ }}{\beta _9}{X_{depth}}\left( i \right) + {\rm{ }}{\beta _{10}}{X_{years}}\left( i \right){\rm{ }} + {\rm{ }}{\beta _{11}}{f_{surface{\rm{ }}processing}}{\left( i \right)_{}}\\& \quad *{\rm{ }}{X_{depth}}\left( i \right){\rm{ }} + {\rm{ }}{\beta _{12}}{f_{surface{\rm{ }}processing}}{\left( i \right)_{}}*{\rm{ }}{X_{years}}\left( i \right){\mkern 1mu} {\rm{and}}\end{array}$$

3 $$\begin{array}{ccccc}{y_{colony}}\left( i \right){\rm{ }} & = {\rm{ }}{\beta _{13}} + {\rm{ }}{\beta _{14}}{f_{surface{\rm{ }}processing}}{\left( i \right)_{}} + {\rm{ }}{\beta _{15}}{X_{depth}}\left( i \right)\\& \quad + {\rm{ }}{\beta _{16}}{X_{years}}\left( i \right){\rm{ }} + {\rm{ }}{\beta _{17}}{f_{surface{\rm{ }}processing}}{\left( i \right)_{}}\\& \quad *{\rm{ }}{X_{depth}}\left( i \right){\rm{ }} + {\rm{ }}{\beta _{18}}{f_{surface{\rm{ }}processing}}{\left( i \right)_{}}*{\rm{ }}{X_{years}}\left( i \right)\end{array}$$

Here, *y*_*cover*_*(i)* is the alr-transformed coral cover to other benthic cover of record *i*; *y*_*colony*_*(i)* is the number of coral colonies of record *i*; *f*_*surface processing*_ is a categorical variable indicating with or without surface processing (i.e., unprocessed area or processed area), with *f*_*surface processing*_ = 0 for unprocessed area and *f*_*surface processing*_ = 1 for processed area; *X*_*years*_ is the years since installation; *X*_*depth*_ is water depth relative to LWL (m); *f*_*surface processing*_ * X_*depth*_ is the interaction between surface processing and water depth; *f*_*surface processing*_ * X_*years*_ is the interaction between surface processing and years since installation; *β*_*7*_ and *β*_*13*_ are intercepts; and *β*_*8*_‒*β*_*12*_ and *β*_*14*_‒*β*_*18*_ are the coefficients of each explanatory variable, respectively. *y*_*cover*_*(i)* follows a normal distribution with mean *µ*_*2*_ and variance *ε*_*2*_^2^ and the link function is set to identity. *y*_*colony*_*(i)* follows a gamma distribution with mean *µ*_*3*_ and variance *ε*_*3*_^2^ and the link function is set to log. The non-informative prior distribution for *ε*_*2*_ and *ε*_*3*_ is a Half-Cauchy distribution with a scale parameter of 10^−2^, the non-informative prior distribution for *β*_*7*_‒*β*_*12*_ is a uniform distribution with lower and upper limits of −10 and 10, and *β*_*13*_‒*β*_*18*_ is a uniform distribution with lower and upper limits of −20 and 20, respectively. After standardization of the explanatory variables, the posterior probability distribution of the standardized coefficient (effect size) for each explanatory variable was estimated by Markov chain Monte Carlo (MCMC) sampling. The model was applied to data for the entire period (1‒18) for each coral taxon to test the long-term effect of surface processing. The model was also applied to available data up to 6 years after construction (1‒6) to test the initial effect of surface processing. MCMC sampling was performed in the same way as for the analysis of the hierarchical Bayesian model of natural coral reefs and artificial structures. The Gelman-Rubin statistics were 1.00. Model fits were assessed with Bayesian *R*^2^ (Supplementary Table S26).

### Survey of coral and fish distributions and net ecosystem calcification in ATPs

Coral and fish distributions were surveyed in 24 ATPs at two depths (top elevations of LWL +1.0 m and +0.7 m): four at each depth with FRP grating, four with grooves (two each of 10-mm and 30-mm width), and four without processing. Each ATP is a box-shaped structure: the inside dimensions of the box are 465 cm in the direction of the breakwater extension, 380–400 cm in the orthogonal direction, and 50–65 cm in depth^31^. Each ATP is separated from the next by a wall or a gap of a few dozen centimeters wide. All ATPs were installed on a 120-m section of the pro-environment breakwater; we therefore considered the conditions other than the installation depth, surface processing, and the physical conditions caused by these to be homogeneous. The distribution of corals on the bottom and inner sides of the ATPs was mapped. Each wall of the ATP was photographed at equal distances, and multiple photographs of each wall were combined into a single photograph by using Photoscan (Agisoft) and Photoshop (Adobe). An expanded diagram of an ATP showing the wall and bottom areas (Fig. 6b) was created from the design drawings and actual measurements, and the photos of each wall were pasted to the diagram after being scaled to fit. Corals were identified at the genus level from the composite photographs on the expanded diagram and mapped on the expanded diagram by outlining each coral colony. Corals that could not be identified from photographs alone were reidentified by field surveys. The maps were loaded into image analysis software (ImageJ-Fiji^77^), and the area (cm^2^) of each mapped coral was determined by determining the length per pixel for each figure, using the length of one side of the base of the ATP for scale on the design drawings. The area (cm^2^) of the bottom and each wall of the ATP (left side [when facing offshore], right side, offshore side, and inshore side; Fig. 6b) and of the processed zone in the center of the bottom were determined in the same way. The coral cover (%) of each ATP box was determined by dividing the total coral area on all walls and the bottom by the total surface area. The number of fish observed in each box was recorded for each fish species.

Differences in alr-transformed coral cover and number of coral colonies between the processed and unprocessed areas on the bottom of the ATPs were compared by paired *t*-test when the data was normally distributed and by Wilcoxon signed rank test when the data normawas not normally distributed. Differences in the area of coral colonies between the processed and unprocessed areas on the bottom of the ATPs were compared by using Welch’s *t*-test when the data was normally distributed, and the Wilcoxon rank sum test when the data was not normally distributed.

For fish, we examined the differences in the number of fish for six combinations of installation depth and surface treatment of the ATP bottom: FRP grating, grooved, and unprocessed areas, installed at LWL +1.0 m or +0.7 m. Data were log transformed to ensure the equivariance of each group. Multiple comparisons were made, and differences were tested by Tukey’s HSD test.

Net calcification rate of ATP ecosystems (NEC, mmol m^−2^ h^−1^) was calculated as follows:

4 $$\:{\text{NEC}} = - \frac{1}{2} \times \:\Delta \:{\text{TA}}/\Delta \:t \times \:\bar{Z}_{t} \times \:\rho \:_{{water}}$$

where ΔTA is the change in total alkalinity (TA, mmol kg^–1^), Δ*t* is the observation time (h), $\bar{Z}_{t}$is the average depth (m) of water at the study site (or inside the ATP) at observation time *t*, and *ρ*_*water*_ is the density of seawater. This equation does not account for the effects of advection and diffusion due to seawater flow. In this study, the equation was applied for measurements at low tide, when ATPs are isolated from the surrounding seawater and any seawater exchange is negligible.

At the ATPs on the First Urasoe Breakwater, water samples were collected from July to November 2019 at 10- to 12-min intervals during 3–4 h when the tide pool rim was exposed; water level, photon flux, water temperature, and salinity in each ATP were observed during the sampling period. There were six daytime water samplings (on 2 July, 31 July, 1 August, 29 August, 30 August, and 28 September 2019) and two night-time water samplings (on 13–14 and 17 November 2019). Water samples were collected from St. 1-3 (ATP top height: LWL +1.0 m, FRP grating), St. 2-3 (LWL +1.0 m, 10-mm grooves), St. 3-3 (LWL +1.0 m, unprocessed), St. 4-3 (LWL +0.7 m, FRP grating), St. 5-3 (LWL +0.7 m, 10-mm grooves), and St. 6-3 (LWL +0.7 m, unprocessed). Surface seawater from inside the ATP at each site was collected in a plastic bag and poured in a 250-mL Schott Duran bottle. Saturated mercuric chloride solution (200 µL) was added to stop biological activity, which can affect dissolved inorganic carbon concentrations. The TA of seawater samples was measured by the Gran plot method, using total alkalinity titration systems (ATT05 and ATT15, Kimoto Electric Co., Ltd., Osaka, Japan); the accuracy was checked with reference materials (KANSO Co., Ltd., Osaka, Japan, and Scripps Institution of Oceanography, La Jolla, California, USA). The accuracy of the TA measurements was less than 3 µmol kg^–1^. The average NEC was determined for the 3–4 h when the tide pools were stagnant during the day and night on each sampling day. For each sampling day, analysis of covariance (ANCOVA) was performed for the average NEC (slope of the temporal change in NEC during the stagnant period from equation^1^) during the sampling period at each site, and multiple comparisons were performed by using the Tukey HSD method.

Daily NEC (mmol CaCO_3_ m^−2^ day^−1^) was calculated as the integral of the regression function of NEC against light intensity. By using the results from daytime sampling in July–September and night-time sampling in November, we determined the NEC and light intensity for three consecutive water samplings (> 30 min), and we then regressed NEC against light intensity to a hyperbolic tangent function or a linear function. By using these regression equations and the daily variations of light intensity on the sampling days from July to September, we estimated the daily NEC for the six sampling days from July to September. The standard deviation of NEC per day was determined by error propagation of the slope of the regression equation and the standard deviation of the intercept to the integral value^78^.

### Relationship between environmental conditions and coral growth on breakwaters

To characterize the environmental conditions suitable for coral growth, we analyzed historical data collected by the Okinawa General Bureau from 1989 to 2017 for the coral distribution on the outer (offshore) sides of three breakwaters in Naha Port (Naha Breakwater, First Shinko Breakwater, and First Urasoe Breakwater). For the coral distribution on the inside of the breakwaters for which historical data were lacking, we conducted coral distribution surveys on the inner (port) side of the First Urasoe Breakwater from 2017 to 2018.

We used historical data to analyze the relationship between water depth or substrate slope and coral cover on the outer side of the breakwaters. The coral distribution surveys were conducted either by the quadrat method, in which a square frame (quadrat) was set up at a fixed point and the coral distribution within the frame was recorded, or the belt transect method, in which the coral distribution was recorded on both sides along a transect line. We used only the data with a clear water-depth standard (i.e., relative to low water level [LWL]). The handling of values of “R” and “+” for the record of coral cover was the same as the method described in the first paragraph of the “Methods” section.

To examine the relationship between water depth and coral cover on the inside of the breakwater, we used the results of coral distribution surveys conducted by using a quadrat on the vertical walls of the inner side of the First Urasoe Breakwater between 2017 and 2018 (*n* = 406). We applied a GAM to the observed data for alr-transformed coral cover and water depth, and for alr-transformed coral cover and substrate slope, by using the “mgcv” package in R. We used generalized cross-validation to determine the degrees of freedom of the model. For the coral distribution on the port side of the breakwater, the number of knots of thin plate regression splines was restricted to seven (*k* = 7), the maximum possible value, because the number of sampling depth variations was only seven. We assumed a normal distribution for the error structure of the model. An inverse alr transformation was applied to convert the values obtained from the regression curves by GAM to coverage. For the cover by each taxon, all the cover by each taxon and the non-coral cover values were inversely alr transformed and then normalized so that the sum of these cover values equaled 100%. After inverse alr transformation of the total coral cover and the non-coral cover, the total coral cover was normalized so that the sum of the two was 100%. In consideration of the zero-value adjustment during the alr transformation, the normalized cover was set to 0% if the normalized cover was less than half (0.25%) of the rounding error. The relationship between light intensity and coral cover was investigated on the basis of coral distribution surveys and observations of light intensity on the inner surfaces of the ATPs (offshore side, inshore side, left side [when facing offshore], right side, and bottom). The offshore side faces south, the inshore side faces north, the left side faces east, and the right side faces west. Coral cover was measured on the walls of each of the 12 ATPs installed at LWL +0.7 m. Light intensity was measured by a photon sensor (DEFI2-L, JFE Advantech Co., Ltd., Hyogo, Japan) for 2 weeks each in summer and winter 2019 on the four walls and bottom of one of the ATPs at LWL +0.7 m (St. 4-3). Differences between the alr-transformed coral cover or light intensity on the four sides and bottom were compared by using Tukey’s HSD test when the data was equivariant, and the Steel-Dwass test when it was not. To investigate the relationship between light intensity and coral cover, we examined the coral cover on the same side in multiple ATPs and the average of the daily integrated light intensity for a total of 28 days: 2 weeks in summer (00:00 21 July – 24:00 3 August 2019, *n* = 14) and 2 weeks in winter (00:00 11 December – 24:00 24 December 2019, *n* = 14). The Anderson-Darling test was used to test the normality of the residuals of the regression line.

Water temperature, salinity, photon flux, and wave height were observed in the field at the First Urasoe Breakwater from 23 June 2018 to 8 July 2019: outside the port at 26° 15″ 29.73″ N, 127° 39″ 18.75″ E and inside the port at 26° 15″ 43.54″ N, 127° 40′ 6.86″ E. Light intensity, temperature, and salinity were observed at LWL −1 m and −3 m on the outer and inner sides of the breakwater, and wave height was observed only at LWL −3 m. Observations were conducted by using water temperature and salinity meters (Compact-CT and Infinity-CT, JFE Advantech Co., Ltd.), water pressure wave height meters (Infinity-WH, JFE Advantech Co., Ltd.), and photometers (DEFI2-L, JFE Advantech Co., Ltd.). Maintenance was performed on the instruments approximately once a month, and observations were carried out for 12 months. Surface transparency (Secchi depth, m) was obtained from the water quality measurements of Okinawa Prefecture from 1995 to 2015. Survey points of surface transparency are shown in Supplementary Fig. S15. The sedimentation status was determined along with the distribution of corals from 2005 to 2017 visually, and by tapping the seafloor surface, by using four qualitative “ranks”: I, no turbidity even after tapping the seafloor; II, turbidity when the seafloor is tapped; III, sparse sediment accumulation; and IV, thick sediment accumulation. Most of the sedimentation in Naha Port was rated as I or II. We compared coral cover under conditions of different sediment accumulation by using the Wilcoxon rank sum test.

## Electronic supplementary material

Below is the link to the electronic supplementary material.

Supplementary Material 1

## Data Availability

The authors declare that the data supporting the findings of this study are available within the paper and its supplementary information file.
