# Supplementary material for: Artificial structures can facilitate rapid coral recovery under climate change
Source: Sci Rep. 2025 Mar 17;15:9116. doi: 10.1038/s41598-025-93531-2 (PMC11914049; doi:10.1038/s41598-025-93531-2)
Supplement: Supplementary file 1 — Supplementary Material 1 [file 41598_2025_93531_MOESM1_ESM.pdf]

Artificial structures can facilitate rapid coral recovery under climate change

Toko Tanaya[1]*, Shunpei Iwamura[2], Wataru Okada[3], and Tomohiro Kuwae[1]

[1]*Coastal and Estuarine Environment Research Group, Port and Airport Research Institute, 3-1-1 Nagase, Yokosuka 239-0826, Japan;* [2]*Daiei Consultant Co., Ltd., 412-4 Minatogawa, Urasoe 901-2134, Japan;* [3]*Incorporated Foundation Okinawa Prefecture Environment Science Center, 720 Kyozuka, Urasoe 901-2111, Japan*

*Corresponding Author: Toko Tanaya (email: tanaya-t@p.mpat.go.jp)

Supplementary Table S1. Results of permutational multivariate analysis of variance testing for differences in coral community composition

Factor	df	SS	MS	F	R^2	P
Substrate	1	7173		934.70	0.167	0.0001
Research year	1	206		26.89	0.005	0.0001
Depth	1	2441		318.14	0.057	0.0001
Substrate*Research year	1	248		32.34	0.006	0.0001
Substrate*Depth	1	1070		139.39	0.025	0.0001
Residual	4155	31886			0.741	

Supplementary Table S2. Comparison between the alr-transformed coral cover on the processed and unprocessed areas of artificial tide pools (ATPs) on the pro-environment breakwater in Naha Port, Japan.

Levels of significance are as follows: *P < 0.05; **P < 0.01; ***P < 0.001; NS, not significant; NA, not applicable.

St.	Surface processing	Taxon	Mean	Median	SD	*n*	Statistical test	*W*	d.f.	*t*	*P*	Levels of significance
St. 1	FRP grating	All hard corals	–1.71	–1.76	0.18	4	Paired *t* -test	-	3	24.73	0.000	***
St. 1	None	All hard corals	–4.29	–4.30	0.11	4						
St. 2	Groove patterns	All hard corals	–2.85	–2.81	0.39	4	Paired *t* -test	-	3	2.85	0.039	*
St. 2	None	All hard corals	–3.49	–3.50	0.21	4						
St. 4	FRP grating	All hard corals	–1.20	–1.22	0.18	4	Paired *t* -test	-	3	8.98	0.000	***
St. 4	None	All hard corals	–2.58	–2.53	0.25	4						
St. 5	Groove patterns	All hard corals	–1.26	–1.22	0.57	4	Paired *t* -test	-	3	3.25	0.035	*
St. 5	None	All hard corals	–2.24	–2.24	0.20	4						
St. 1	FRP grating	*Acropora*	–2.43	–2.44	0.28	4	Paired *t* -test	-	3	9.06	0.001	**
St. 1	None	*Acropora*	–6.55	–6.66	0.87	4						
St. 2	Groove patterns	*Acropora*	–4.17	–4.28	0.35	4	Paired *t* -test	-	3	4.75	0.006	**
St. 2	None	*Acropora*	–5.80	–5.78	0.59	4						
St. 4	FRP grating	*Acropora*	–1.95	–1.92	0.29	4	Paired *t* -test	-	3	9.71	0.000	***
St. 4	None	*Acropora*	–5.10	–5.17	0.58	4						
St. 5	Groove patterns	*Acropora*	–1.85	–1.73	0.87	4	Paired *t* -test	-	3	4.95	0.006	**
St. 5	None	*Acropora*	–4.23	–4.17	0.42	4						
St. 1	FRP grating	*Millepora*	–7.00	–7.02	0.76	4	Paired *t* -test	-	3	0.40	0.708	NS
St. 1	None	*Millepora*	–7.36	–7.26	1.60	4						
St. 2	Groove patterns	*Millepora*	–6.14	–5.94	0.69	4	Wilcoxon signed rank test	0	-	-	0.125	NS
St. 2	None	*Millepora*	–7.65	–7.62	1.95	4						
St. 4	FRP grating	*Millepora*	–3.54	–3.63	0.56	4	Wilcoxon signed rank test	0	-	-	0.125	NS
St. 4	None	*Millepora*	–6.03	–5.62	1.00	4						
St. 5	Groove patterns	*Millepora*	–4.40	–4.51	0.51	4	Paired *t* -test	-	3	7.08	0.001	***
St. 5	None	*Millepora*	–7.54	–7.68	0.73	4						
St. 1	FRP grating	Faviidae	–4.44	–4.43	0.35	4	Wilcoxon signed rank test	0	-	-	0.125	NS
St. 1	None	Faviidae	–5.39	–5.20	0.42	4						
St. 2	Groove patterns	Faviidae	–4.25	–4.25	0.79	4	Paired *t* -test	-	3	0.29	0.783	NS
St. 2	None	Faviidae	–4.37	–4.37	0.05	4						
St. 4	FRP grating	Faviidae	–4.19	–4.13	0.57	4	Paired *t* -test	-	3	0.49	0.642	NS
St. 4	None	Faviidae	–4.37	–4.42	0.46	4						
St. 5	Groove patterns	Faviidae	–3.48	–3.44	0.33	4	Paired *t* -test	-	3	0.35	0.739	NS
St. 5	None	Faviidae	–3.59	–3.52	0.49	4						
St. 1	FRP grating	*Montipora*	–6.73	–6.86	0.76	4	Paired *t* -test	-	3	0.72	0.518	NS
St. 1	None	*Montipora*	–7.61	–6.78	2.32	4						
St. 2	Groove patterns	*Montipora*	–6.35	–6.20	0.54	4	Paired *t* -test	-	3	1.32	0.247	NS
St. 2	None	*Montipora*	–7.07	–7.08	0.95	4						
St. 4	FRP grating	*Montipora*	–5.24	–5.22	0.87	4	Paired *t* -test	-	3	3.04	0.023	*
St. 4	None	*Montipora*	–7.24	–7.44	0.98	4						
St. 5	Groove patterns	*Montipora*	–4.87	–4.76	0.82	4	Paired *t* -test	-	3	3.06	0.031	*
St. 5	None	*Montipora*	–7.46	–7.29	1.48	4						
St. 1	FRP grating	*Porites*	–3.67	–3.60	0.57	4	Paired *t* -test	-	3	5.30	0.007	**
St. 1	None	*Porites*	–5.29	–5.32	0.23	4						
St. 2	Groove patterns	*Porites*	–4.07	–4.10	0.26	4	Paired *t* -test	-	3	1.58	0.170	NS
St. 2	None	*Porites*	–4.42	–4.34	0.36	4						
St. 4	FRP grating	*Porites*	–2.58	–2.61	0.11	4	Paired *t* -test	-	3	2.07	0.120	NS
St. 4	None	*Porites*	–3.03	–2.94	0.42	4						
St. 5	Groove patterns	*Porites*	–2.93	–2.93	0.13	4	Paired *t* -test	-	3	–1.493	0.213	NS
St. 5	None	*Porites*	–2.82	–2.81	0.05	4						
St. 1	FRP grating	*Pocillopora*	–3.25	–3.29	0.54	4	Paired *t* -test	-	3	9.36	0.000	***
St. 1	None	*Pocillopora*	–6.60	–6.51	0.47	4						
St. 2	Groove patterns	*Pocillopora*	–5.41	–5.47	0.42	4	Paired *t* -test	-	3	2.27	0.093	NS
St. 2	None	*Pocillopora*	–6.96	–6.56	1.30	4						
St. 4	FRP grating	*Pocillopora*	–3.70	–3.60	0.37	4	Paired *t* -test	-	3	7.58	0.001	***
St. 4	None	*Pocillopora*	–6.50	–6.43	0.64	4						
St. 5	Groove patterns	*Pocillopora*	–5.25	–5.35	1.25	4	Paired *t* -test	-	3	2.41	0.055	NS
St. 5	None	*Pocillopora*	–7.15	–7.07	0.96	4						

Supplementary Table S3. Comparison between the number of coral colonies per square meter on the processed and unprocessed areas of artificial tide pools (ATPs) on the pro-environment breakwater in Naha Port, Japan.

Levels of significance are as follows: $^{*}P < 0.05$; $^{**}P < 0.01$; $^{***}P < 0.001$; NS, not significant; NA, not applicable.

St.	Surface processing	Taxon	Mean	Median	SD	*n*	Statistical test	*W*	d.f.	*t*	*P*	Levels of
St. 1	FRP grating	All hard corals	35.78	31.88	9.60	4	Wilcoxon signed rank test	0	-	-	0.125	NS
St. 1	None	All hard corals	7.40	7.51	1.37	4						
St. 2	Groove patterns	All hard corals	23.48	19.20	10.40	4	Wilcoxon signed rank test	0	-	-	0.125	NS
St. 2	None	All hard corals	12.49	11.88	2.58	4						
St. 4	FRP grating	All hard corals	37.38	38.50	4.17	4	Paired *t* -test	-	3	7.14	0.000	***
St. 4	None	All hard corals	18.03	18.77	3.47	4						
St. 5	Groove patterns	All hard corals	43.54	44.42	17.01	4	Paired *t* -test	-	3	2.53	0.045	*
St. 5	None	All hard corals	21.88	20.96	2.24	4						
St. 1	FRP grating	*Acropora*	15.07	12.95	6.65	4	Paired *t* -test	-	3	4.17	0.006	**
St. 1	None	*Acropora*	1.11	1.08	0.78	4						
St. 2	Groove patterns	*Acropora*	4.81	4.85	0.83	4	Paired *t* -test	-	3	8.3	0.001	**
St. 2	None	*Acropora*	1.08	1.13	0.34	4						
St. 4	FRP grating	*Acropora*	13.71	13.81	1.46	4	Paired *t* -test	-	3	13.52	0.000	***
St. 4	None	*Acropora*	1.61	1.24	1.04	4						
St. 5	Groove patterns	*Acropora*	20.72	21.79	13.38	4	Paired *t* -test	-	3	2.78	0.032	*
St. 5	None	*Acropora*	2.11	1.95	0.77	4						
St. 1	FRP grating	*Millepora*	0.18	0.18	0.00	4	Wilcoxon signed rank test	0	-	-	0.125	NS
St. 1	None	*Millepora*	0.04	0.04	0.04	4						
St. 2	Groove patterns	*Millepora*	0.35	0.26	0.42	4	Wilcoxon signed rank test	1	-	-	0.285	NS
St. 2	None	*Millepora*	0.04	0.04	0.05	4						
St. 4	FRP grating	*Millepora*	2.73	2.61	0.72	4	Paired *t* -test	-	3	5.97	0.005	**
St. 4	None	*Millepora*	0.45	0.54	0.26	4						
St. 5	Groove patterns	*Millepora*	1.92	1.52	1.56	4	Paired *t* -test	-	3	2.22	0.068	NS
St. 5	None	*Millepora*	0.19	0.21	0.08	4						
St. 1	FRP grating	Faviidae	3.43	3.27	1.06	4	Paired *t* -test	-	3	–0.57	0.593	NS
St. 1	None	Faviidae	3.78	3.58	0.62	4						
St. 2	Groove patterns	Faviidae	10.25	7.30	7.88	4	Paired *t* -test	-	3	0.76	0.477	NS
St. 2	None	Faviidae	7.21	6.69	1.46	4						
St. 4	FRP grating	Faviidae	2.37	2.40	0.76	4	Paired *t* -test	-	3	–1.68	0.175	NS
St. 4	None	Faviidae	4.39	4.20	2.28	4						
St. 5	Groove patterns	Faviidae	8.21	7.48	2.13	4	Paired *t* -test	-	3	–0.82	0.442	NS
St. 5	None	Faviidae	9.50	9.17	2.28	4						
St. 1	FRP grating	*Montipora*	0.27	0.27	0.23	4	Wilcoxon signed rank test	0	-	-	0.109	NS
St. 1	None	*Montipora*	0.04	0.04	0.04	4						
St. 2	Groove patterns	*Montipora*	0.30	0.26	0.36	4	Wilcoxon signed rank test	1	-	-	0.285	NS
St. 2	None	*Montipora*	0.12	0.16	0.08	4						
St. 4	FRP grating	*Montipora*	0.68	0.73	0.28	4	Paired *t* -test	-	3	4.18	0.006	**
St. 4	None	*Montipora*	0.08	0.08	0.07	4						
St. 5	Groove patterns	*Montipora*	0.46	0.50	0.37	4	Paired *t* -test	-	3	1.99	0.093	NS
St. 5	None	*Montipora*	0.08	0.08	0.07	4						
St. 1	FRP grating	*Porites*	5.76	5.70	2.30	4	Paired *t* -test	-	3	3.61	0.011	*
St. 1	None	*Porites*	1.59	1.57	0.22	4						
St. 2	Groove patterns	*Porites*	4.82	3.83	2.24	4	Wilcoxon signed rank test	1	-	-	0.250	NS
St. 2	None	*Porites*	3.35	3.32	1.18	4						
St. 4	FRP grating	*Porites*	13.70	13.50	2.49	4	Paired *t* -test	-	3	1.46	0.195	NS
St. 4	None	*Porites*	10.92	11.45	2.88	4						
St. 5	Groove patterns	*Porites*	10.47	10.24	1.18	4	Paired *t* -test	-	3	1.16	0.293	NS
St. 5	None	*Porites*	9.34	9.11	1.54	4						
St. 1	FRP grating	*Pocillopora*	10.07	8.93	4.40	4	Paired *t* -test	-	3	4.23	0.005	**
St. 1	None	*Pocillopora*	0.75	0.73	0.21	4						
St. 2	Groove patterns	*Pocillopora*	2.44	2.60	0.94	4	Paired *t* -test	-	3	3.79	0.023	*
St. 2	None	*Pocillopora*	0.57	0.63	0.30	4						
St. 4	FRP grating	*Pocillopora*	3.91	3.86	1.27	4	Paired *t* -test	-	3	5.36	0.002	**
St. 4	None	*Pocillopora*	0.48	0.50	0.18	4						
St. 5	Groove patterns	*Pocillopora*	1.67	1.40	1.14	4	Paired *t* -test	-	3	2.13	0.078	NS
St. 5	None	*Pocillopora*	0.44	0.47	0.22	4						

Supplementary Table S4. Comparison between the colony size of corals on the processed and unprocessed areas of artificial tide pools (ATPs) on the pro-environment breakwater in Naha Port, Japan.

Levels of significance are as follows: **P* < 0.05; ***P* < 0.01; ****P* < 0.001; NS, not significant; NA, not applicable.

St.	Surface processing	Taxon	Mean	Median	SD	*n*	Statistical test	*W*	d.f.	*t*	*P*	Levels of significance
St. 1	FRP grating	All hard corals	42.94	31.98	34.89	792	Wilcoxon rank sum test	########	-	-	0.000	***
St. 1	None	All hard corals	18.41	10.12	21.88	385						
St. 2	Groove patterns	All hard corals	24.34	17.45	21.94	541	Wilcoxon rank sum test	########	-	-	0.379	NS
St. 2	None	All hard corals	24.13	16.81	23.33	638						
St. 4	FRP grating	All hard corals	62.35	47.32	54.31	823	Wilcoxon rank sum test	########	-	-	0.000	***
St. 4	None	All hard corals	39.92	29.40	35.46	871						
St. 5	Groove patterns	All hard corals	53.40	40.32	45.79	1015	Wilcoxon rank sum test	########	-	-	0.000	***
St. 5	None	All hard corals	44.46	31.34	43.59	1036						
St. 1	FRP grating	*Acropora*	50.40	39.35	40.63	334	Wilcoxon rank sum test	15846.00	-	-	0.000	***
St. 1	None	*Acropora*	16.62	9.28	20.82	58						
St. 2	Groove patterns	*Acropora*	31.76	23.18	27.03	111	Wilcoxon rank sum test	3143.00	-	-	0.757	NS
St. 2	None	*Acropora*	30.92	22.78	26.88	55						
St. 4	FRP grating	*Acropora*	81.01	67.75	61.93	302	Wilcoxon rank sum test	17393.00	-	-	0.000	***
St. 4	None	*Acropora*	40.56	31.75	35.86	78						
St. 5	Groove patterns	*Acropora*	69.18	58.75	46.59	488	Wilcoxon rank sum test	26688.50	-	-	0.139	NS
St. 5	None	*Acropora*	65.90	55.16	57.59	100						
St. 1	FRP grating	*Millepora*	52.84	42.14	40.08	4	NA	NA	NA	NA	NA	NA
St. 1	None	*Millepora*	20.95	20.95	5.41	2						
St. 2	Groove patterns	*Millepora*	31.32	26.65	18.15	8	NA	NA	NA	NA	NA	NA
St. 2	None	*Millepora*	10.95	10.95	0.84	2						
St. 4	FRP grating	*Millepora*	89.47	72.29	72.13	60	Wilcoxon rank sum test	826.50	-	-	0.082	NS
St. 4	None	*Millepora*	64.46	51.06	65.45	22						
St. 5	Groove patterns	*Millepora*	51.84	28.91	52.97	45	Wilcoxon rank sum test	264.00	-	-	0.157	NS
St. 5	None	*Millepora*	30.61	18.80	41.54	9						
St. 1	FRP grating	Faviidae	30.19	23.26	29.53	76	Wilcoxon rank sum test	12199.00	-	-	0.000	***
St. 1	None	Faviidae	12.64	8.52	14.52	197						
St. 2	Groove patterns	Faviidae	16.23	13.34	12.00	236	Wilcoxon rank sum test	45397.50	-	-	0.346	NS
St. 2	None	Faviidae	17.13	12.07	17.18	368						
St. 4	FRP grating	Faviidae	54.85	45.85	40.32	52	Wilcoxon rank sum test	8327.50	-	-	0.000	***
St. 4	None	Faviidae	28.85	15.34	32.83	212						
St. 5	Groove patterns	Faviidae	29.85	19.27	30.08	189	Wilcoxon rank sum test	43503.50	-	-	0.646	NS
St. 5	None	Faviidae	28.61	19.28	26.76	450						
St. 1	FRP grating	*Montipora*	23.16	19.96	16.61	6	NA	NA	NA	NA	NA	NA
St. 1	None	*Montipora*	34.06	34.06	44.78	2						
St. 2	Groove patterns	*Montipora*	18.72	11.71	15.36	7	Wilcoxon rank sum test	12.00	-	-	0.234	NS
St. 2	None	*Montipora*	42.86	20.12	44.21	6						
St. 4	FRP grating	*Montipora*	78.48	44.05	104.47	15	Wilcoxon rank sum test	40.00	-	-	0.357	NS
St. 4	None	*Montipora*	41.98	33.18	29.25	4						
St. 5	Groove patterns	*Montipora*	137.54	73.53	119.45	11	Welch's *t*-test	-	11.70	2.04	0.064	NS
St. 5	None	*Montipora*	44.74	21.98	55.31	4						
St. 1	FRP grating	*Porites*	42.33	33.70	29.21	127	Wilcoxon rank sum test	6696.50	-	-	0.001	***
St. 1	None	*Porites*	31.78	20.71	29.03	83						
St. 2	Groove patterns	*Porites*	34.16	28.60	26.60	111	Wilcoxon rank sum test	9021.50	-	-	0.484	NS
St. 2	None	*Porites*	36.58	31.07	27.83	171						
St. 4	FRP grating	*Porites*	42.59	34.65	32.83	302	Wilcoxon rank sum test	79698.00	-	-	0.971	NS
St. 4	None	*Porites*	43.55	34.49	33.51	527						
St. 5	Groove patterns	*Porites*	38.87	31.86	27.19	241	Wilcoxon rank sum test	41262.50	-	-	0.000	***
St. 5	None	*Porites*	57.54	43.34	48.48	442						
St. 1	FRP grating	*Pocillopora*	36.04	27.72	26.17	223	Wilcoxon rank sum test	6514.50	-	-	0.000	***
St. 1	None	*Pocillopora*	19.42	12.99	22.69	39						
St. 2	Groove patterns	*Pocillopora*	18.45	14.42	13.33	56	Wilcoxon rank sum test	644.00	-	-	0.121	NS
St. 2	None	*Pocillopora*	24.70	23.74	16.32	29						
St. 4	FRP grating	*Pocillopora*	50.22	41.62	36.56	86	Wilcoxon rank sum test	1417.50	-	-	0.001	**
St. 4	None	*Pocillopora*	33.90	19.60	40.43	23						
St. 5	Groove patterns	*Pocillopora*	38.71	23.81	48.56	39	Wilcoxon rank sum test	456.00	-	-	0.479	NS
St. 5	None	*Pocillopora*	21.77	23.19	11.41	21						

Supplementary Table S5. Daily net ecosystem calcification rate (NEC) for each artificial tide pool (ATP) station during each observation period, based on regression curves.

Site	Starting date	Ending date	Regression type	NEC (mmol $CaCO_3$ m^{-2} day^{-1})	NEC (g $CaCO_3$ m^{-2} day^{-1})	Integrated photon flux (mol m^{-2} day^{-1})
St. 1-3	2019/7/2	2019/7/3	Hyperbolic tangent	99	8	6
St. 1-3	2019/7/31	2019/8/1	Hyperbolic tangent	113	7	10
St. 1-3	2019/8/1	2019/8/2	Hyperbolic tangent	113	7	11
St. 1-3	2019/8/29	2019/8/30	Hyperbolic tangent	107	7	9
St. 1-3	2019/8/30	2019/8/31	Hyperbolic tangent	109	7	9
St. 1-3	2019/9/28	2019/9/29	Hyperbolic tangent	104	8	7
St. 2-3	2019/7/31	2019/8/1	Hyperbolic tangent	103	6	10
St. 2-3	2019/8/1	2019/8/2	Hyperbolic tangent	107	6	11
St. 2-3	2019/8/29	2019/8/30	Hyperbolic tangent	103	6	9
St. 2-3	2019/8/30	2019/8/31	Hyperbolic tangent	101	7	8
St. 2-3	2019/9/28	2019/9/29	Hyperbolic tangent	101	7	7
St. 3-3	2019/8/29	2019/8/30	Linear	101	10	9
St. 3-3	2019/8/30	2019/8/31	Linear	104	10	9
St. 3-3	2019/9/28	2019/9/29	Linear	88	11	7
St. 4-3	2019/7/2	2019/7/3	Hyperbolic tangent	138	10	7
St. 4-3	2019/7/31	2019/8/1	Hyperbolic tangent	143	9	9
St. 4-3	2019/8/1	2019/8/2	Hyperbolic tangent	139	10	8
St. 4-3	2019/8/29	2019/8/30	Hyperbolic tangent	121	10	6
St. 4-3	2019/8/30	2019/8/31	Hyperbolic tangent	128	9	8
St. 4-3	2019/9/28	2019/9/29	Hyperbolic tangent	122	9	6
St. 5-3	2019/7/31	2019/8/1	Hyperbolic tangent	155	12	12
St. 5-3	2019/8/1	2019/8/2	Hyperbolic tangent	163	12	12
St. 5-3	2019/8/29	2019/8/30	Hyperbolic tangent	153	12	10
St. 5-3	2019/8/30	2019/8/31	Hyperbolic tangent	159	13	9
St. 5-3	2019/9/28	2019/9/29	Hyperbolic tangent	155	14	7
St. 6-3	2019/7/31	2019/8/1	Linear	89	5	9
St. 6-3	2019/8/1	2019/8/2	Linear	93	5	10
St. 6-3	2019/8/29	2019/8/30	Linear	87	5	9
St. 6-3	2019/8/30	2019/8/31	Linear	81	6	8
St. 6-3	2019/9/28	2019/9/29	Linear	65	6	6

Supplementary Table S6. Comparison between light intensity (μmol-photons m^{-2} s^{-1}) on the outer side of the First Urasoe Breakwater in Naha Port, Japan, at LWL –1 m and LWL –3 m in each month.

Observed Year-Month	Station	Mean	Median	SD	*n*	Statistical test	Statistics	*P*	d.f.	Significance	Notes
2018-07	Outer side of the breakwater at LWL – 1 m	220.42	257.43	96.06	31	Wilcoxon rank sum test	755.00	0.000		TRUE	**Maybe significant** (are not normal distribution and not equal variance)
2018-07	Outer side the breakwater at LWL – 3 m	136.95	146.52	57.26	31	Wilcoxon rank sum test	755.00	0.000		TRUE	**Maybe significant** (are not normal distribution and not equal variance)
2018-08	Outer side of the breakwater at LWL – 1 m	146.39	101.36	88.71	31	Wilcoxon rank sum test	481.00	1.000		FALSE	Not significant (are not normal distribution and not equal variance)
2018-08	Outer side the breakwater at LWL – 3 m	134.89	129.35	77.88	31	Wilcoxon rank sum test	481.00	1.000		FALSE	Not significant (are not normal distribution and not equal variance)
2018-09	Outer side of the breakwater at LWL – 1 m	106.37	78.59	87.48	28	Wilcoxon rank sum test	309.00	0.178		FALSE	Not significant (are not normal distribution and not equal variance)
2018-09	Outer side the breakwater at LWL – 3 m	116.82	109.09	64.01	28	Wilcoxon rank sum test	309.00	0.178		FALSE	Not significant (are not normal distribution and not equal variance)
2018-10	Outer side of the breakwater at LWL – 1 m	84.06	71.26	59.64	22	Student's *t* -test	4.03	0.000	42	TRUE	**Maybe significant** (are normal distribution and equal variance)
2018-10	Outer side the breakwater at LWL – 3 m	31.27	32.07	14.88	22	Student's *t* -test	4.03	0.000	42	TRUE	**Maybe significant** (are normal distribution and equal variance)
2018-11	Outer side of the breakwater at LWL – 1 m	99.22	92.78	50.41	25	Student's *t* -test	6.63	0.000	48	TRUE	**Maybe significant** (are normal distribution and equal variance)
2018-11	Outer side the breakwater at LWL – 3 m	31.75	31.26	7.13	25	Student's *t* -test	6.63	0.000	48	TRUE	**Maybe significant** (are normal distribution and equal variance)
2018-12	Outer side of the breakwater at LWL – 1 m	56.40	49.78	42.79	27	Wilcoxon rank sum test	409.00	0.024		TRUE	**Maybe significant** (are not normal distribution and not equal variance)
2018-12	Outer side the breakwater at LWL – 3 m	24.55	24.88	11.88	22	Wilcoxon rank sum test	409.00	0.024		TRUE	**Maybe significant** (are not normal distribution and not equal variance)
2019-01	Outer side of the breakwater at LWL – 1 m	66.66	51.03	49.14	23	Wilcoxon rank sum test	399.00	0.003		TRUE	**Maybe significant** (are not normal distribution and not equal variance)
2019-01	Outer side the breakwater at LWL – 3 m	25.48	27.52	10.20	23	Wilcoxon rank sum test	399.00	0.003		TRUE	**Maybe significant** (are not normal distribution and not equal variance)
2019-02	Outer side of the breakwater at LWL – 1 m	78.18	82.35	49.98	26	Student's *t* -test	4.52	0.000	50	TRUE	**Maybe significant** (are normal distribution and equal variance)
2019-02	Outer side the breakwater at LWL – 3 m	31.72	32.31	15.84	26	Student's *t* -test	4.52	0.000	50	TRUE	**Maybe significant** (are normal distribution and equal variance)
2019-03	Outer side of the breakwater at LWL – 1 m	77.79	60.94	49.54	18	Wilcoxon rank sum test	284.00	0.000		TRUE	**Maybe significant** (are not normal distribution and not equal variance)
2019-03	Outer side the breakwater at LWL – 3 m	29.89	31.19	9.08	18	Wilcoxon rank sum test	284.00	0.000		TRUE	**Maybe significant** (are not normal distribution and not equal variance)
2019-04	Outer side of the breakwater at LWL – 1 m	173.16	163.74	92.52	23	Student's *t* -test	4.27	0.000	44	TRUE	**Maybe significant** (are normal distribution and equal variance)
2019-04	Outer side the breakwater at LWL – 3 m	84.54	90.46	36.93	23	Student's *t* -test	4.27	0.000	44	TRUE	**Maybe significant** (are normal distribution and equal variance)
2019-05	Outer side of the breakwater at LWL – 1 m	207.58	221.10	105.26	29	Student's *t* -test	4.69	0.000	56	TRUE	**Maybe significant** (are normal distribution and equal variance)
2019-05	Outer side the breakwater at LWL – 3 m	105.18	105.33	52.28	29	Student's *t* -test	4.69	0.000	56	TRUE	**Maybe significant** (are normal distribution and equal variance)
2019-06	Outer side of the breakwater at LWL – 1 m	181.36	142.42	103.27	24	Student's *t* -test	3.61	0.001	46	TRUE	**Maybe significant** (are normal distribution and equal variance)
2019-06	Outer side the breakwater at LWL – 3 m	94.91	77.49	55.42	24	Student's *t* -test	3.61	0.001	46	TRUE	**Maybe significant** (are normal distribution and equal variance)

Supplementary Table S7. Comparison between light intensity (μmol-photons m^{-2} s^{-1}) on the outer and inner sides of the First Urasoe Breakwater in Naha Port, Japan, at LWL –1 m in each month.

Observed Year-Month	Station	Mean	Median	SD	*n*	Statistical test	Statistics	*P*	d.f.	Significance	Notes
2018-07	Outer side of the breakwater at LWL – 1 m	220.42	257.43	96.06	31	Wilcoxon rank sum test	775.00	0.000		TRUE	**Maybe significant** (are not normal distribution and not equal variance)
2018-07	Inner side of the breakwater at LWL – 1 m	124.08	139.78	56.24	31	Wilcoxon rank sum test	775.00	0.000		TRUE	**Maybe significant** (are not normal distribution and not equal variance)
2018-08	Outer side of the breakwater at LWL – 1 m	146.39	101.36	88.71	31	Wilcoxon rank sum test	499.00	0.801		FALSE	Not significant (are not normal distribution and not equal variance)
2018-08	Inner side of the breakwater at LWL – 1 m	128.68	135.45	59.87	31	Wilcoxon rank sum test	499.00	0.801		FALSE	Not significant (are not normal distribution and not equal variance)
2018-09	Outer side of the breakwater at LWL – 1 m	106.37	78.59	87.48	28	Wilcoxon rank sum test	242.00	0.013		TRUE	**Maybe significant** (are not normal distribution and not equal variance)
2018-09	Inner side of the breakwater at LWL – 1 m	140.59	146.01	74.65	28	Wilcoxon rank sum test	242.00	0.013		TRUE	**Maybe significant** (are not normal distribution and not equal variance)
2018-10	Outer side of the breakwater at LWL – 1 m	84.06	71.26	59.64	22	Wilcoxon rank sum test	235.00	0.880		FALSE	Not significant (are not normal distribution and not equal variance)
2018-10	Inner side of the breakwater at LWL – 1 m	90.73	91.30	66.01	22	Wilcoxon rank sum test	235.00	0.880		FALSE	Not significant (are not normal distribution and not equal variance)
2018-11	Outer side of the breakwater at LWL – 1 m	99.22	92.78	50.41	25	Welch's *t* -test	–0.37	0.714	47.03	FALSE	Not significant (are normal distribution but not equal variance)
2018-11	Inner side of the breakwater at LWL – 1 m	104.13	107.62	43.61	25	Welch's *t* -test	–0.37	0.714	47.03	FALSE	Not significant (are normal distribution but not equal variance)
2018-12	Outer side of the breakwater at LWL – 1 m	56.40	49.78	42.79	27	Wilcoxon rank sum test	300.00	0.742		FALSE	Not significant (are not normal distribution and not equal variance)
2018-12	Inner side of the breakwater at LWL – 1 m	53.16	51.84	36.31	21	Wilcoxon rank sum test	300.00	0.742		FALSE	Not significant (are not normal distribution and not equal variance)
2019-01	Outer side of the breakwater at LWL – 1 m	66.66	51.03	49.14	23	Wilcoxon rank sum test	288.00	0.617		FALSE	Not significant (are not normal distribution and not equal variance)
2019-01	Inner side of the breakwater at LWL – 1 m	59.56	44.07	44.89	23	Wilcoxon rank sum test	288.00	0.617		FALSE	Not significant (are not normal distribution and not equal variance)
2019-02	Outer side of the breakwater at LWL – 1 m	78.18	82.35	49.98	26	Welch's *t* -test	0.70	0.487	49.18	FALSE	Not significant (are normal distribution but not equal variance)
2019-02	Inner side of the breakwater at LWL – 1 m	69.05	61.94	43.88	26	Welch's *t* -test	0.70	0.487	49.18	FALSE	Not significant (are normal distribution but not equal variance)
2019-03	Outer side of the breakwater at LWL – 1 m	77.79	60.94	49.54	18	Welch's *t* -test	–0.52	0.608	34.00	FALSE	Not significant (are normal distribution but not equal variance)
2019-03	Inner side of the breakwater at LWL – 1 m	86.30	82.72	48.98	18	Welch's *t* -test	–0.52	0.608	34.00	FALSE	Not significant (are normal distribution but not equal variance)
2019-04	Outer side of the breakwater at LWL – 1 m	173.16	163.74	92.52	23	Student's *t* -test	3.16	0.003	44	TRUE	**Maybe significant** (are normal distribution and equal variance)
2019-04	Inner side of the breakwater at LWL – 1 m	100.49	101.30	60.18	23	Student's *t* -test	3.16	0.003	44	TRUE	**Maybe significant** (are normal distribution and equal variance)
2019-05	Outer side of the breakwater at LWL – 1 m	207.58	221.10	105.26	29	Student's *t* -test	5.36	0.000	56	TRUE	**Maybe significant** (are normal distribution and equal variance)
2019-05	Inner side of the breakwater at LWL – 1 m	93.37	93.32	45.78	29	Student's *t* -test	5.36	0.000	56	TRUE	**Maybe significant** (are normal distribution and equal variance)
2019-06	Outer side of the breakwater at LWL – 1 m	181.36	142.42	103.27	24	Student's *t* -test	5.20	0.000	46	TRUE	**Maybe significant** (are normal distribution and equal variance)
2019-06	Inner side of the breakwater at LWL – 1 m	63.58	53.57	40.72	24	Student's *t* -test	5.20	0.000	46	TRUE	**Maybe significant** (are normal distribution and equal variance)

Supplementary Table S8. Comparison between light intensity (μmol-photons m^{-2} s^{-1}) on the outer side of the First Urasoe Breakwater in Naha Port, Japan, at LWL –3 m and the inner side at LWL –1 m in each month.

Observed Year-Month	Station	Mean	Median	SD	*n*	Statistical test	Statistics	*P*	d.f.	Significance	Notes
2018-07	Outer side of the breakwater at LWL –3 m	136.95	146.52	57.26	31	Wilcoxon rank sum test	563.00	0.250		FALSE	Not significant (are not normal distribution and not equal variance)
2018-07	Inner side of the breakwater at LWL –1 m	124.08	139.78	56.24	31	Wilcoxon rank sum test	563.00	0.250		FALSE	Not significant (are not normal distribution and not equal variance)
2018-08	Outer side of the breakwater at LWL –3 m	134.89	129.35	77.88	31	Welch's *t* -test	0.35	0.726	56.28	FALSE	Not significant (are normal distribution but not equal variance)
2018-08	Inner side of the breakwater at LWL –1 m	128.68	135.45	59.87	31	Welch's *t* -test	0.35	0.726	56.28	FALSE	Not significant (are normal distribution but not equal variance)
2018-09	Outer side of the breakwater at LWL –3 m	116.82	109.09	64.01	28	Welch's *t* -test	–1.28	0.206	52.77	FALSE	Not significant (are normal distribution but not equal variance)
2018-09	Inner side of the breakwater at LWL –1 m	140.59	146.01	74.65	28	Welch's *t* -test	–1.28	0.206	52.77	FALSE	Not significant (are normal distribution but not equal variance)
2018-10	Outer side of the breakwater at LWL –3 m	31.27	32.07	14.88	22	Wilcoxon rank sum test	133.00	0.010		TRUE	**Maybe significant** (are not normal distribution and not equal variance)
2018-10	Inner side of the breakwater at LWL –1 m	90.73	91.30	66.01	22	Wilcoxon rank sum test	133.00	0.010		TRUE	**Maybe significant** (are not normal distribution and not equal variance)
2018-11	Outer side of the breakwater at LWL –3 m	31.75	31.26	7.13	25	Student's *t* -test	–8.19	0.000	48	TRUE	**Maybe significant** (are normal distribution and equal variance)
2018-11	Inner side of the breakwater at LWL –1 m	104.13	107.62	43.61	25	Student's *t* -test	–8.19	0.000	48	TRUE	**Maybe significant** (are normal distribution and equal variance)
2018-12	Outer side of the breakwater at LWL –3 m	24.55	24.88	11.88	22	Student's *t* -test	–3.51	0.001	41	TRUE	**Maybe significant** (are normal distribution and equal variance)
2018-12	Inner side of the breakwater at LWL –1 m	53.16	51.84	36.31	21	Student's *t* -test	–3.51	0.001	41	TRUE	**Maybe significant** (are normal distribution and equal variance)
2019-01	Outer side of the breakwater at LWL –3 m	25.48	27.52	10.20	23	Wilcoxon rank sum test	158.00	0.019		TRUE	**Maybe significant** (are not normal distribution and not equal variance)
2019-01	Inner side of the breakwater at LWL –1 m	59.56	44.07	44.89	23	Wilcoxon rank sum test	158.00	0.019		TRUE	**Maybe significant** (are not normal distribution and not equal variance)
2019-02	Outer side of the breakwater at LWL –3 m	31.72	32.31	15.84	26	Student's *t* -test	–4.08	0.000	50	TRUE	**Maybe significant** (are normal distribution and equal variance)
2019-02	Inner side of the breakwater at LWL –1 m	69.05	61.94	43.88	26	Student's *t* -test	–4.08	0.000	50	TRUE	**Maybe significant** (are normal distribution and equal variance)
2019-03	Outer side of the breakwater at LWL –3 m	29.89	31.19	9.08	18	Wilcoxon rank sum test	45.00	0.000		TRUE	**Maybe significant** (are not normal distribution and not equal variance)
2019-03	Inner side of the breakwater at LWL –1 m	86.30	82.72	48.98	18	Wilcoxon rank sum test	45.00	0.000		TRUE	**Maybe significant** (are not normal distribution and not equal variance)
2019-04	Outer side of the breakwater at LWL –3 m	84.54	90.46	36.93	23	Student's *t* -test	–1.08	0.285	44	FALSE	Not significant (are normal distribution and equal variance)
2019-04	Inner side of the breakwater at LWL –1 m	100.49	101.30	60.18	23	Student's *t* -test	–1.08	0.285	44	FALSE	Not significant (are normal distribution and equal variance)
2019-05	Outer side of the breakwater at LWL –3 m	105.18	105.33	52.28	29	Welch's *t* -test	0.92	0.364	55.04	FALSE	Not significant (are normal distribution but not equal variance)
2019-05	Inner side of the breakwater at LWL –1 m	93.37	93.32	45.78	29	Welch's *t* -test	0.92	0.364	55.04	FALSE	Not significant (are normal distribution but not equal variance)
2019-06	Outer side of the breakwater at LWL –3 m	94.91	77.49	55.42	24	Welch's *t* -test	2.23	0.031	42.23	TRUE	**Maybe significant** (are normal distribution but not equal variance)
2019-06	Inner side of the breakwater at LWL –1 m	63.58	53.57	40.72	24	Welch's *t* -test	2.23	0.031	42.23	TRUE	**Maybe significant** (are normal distribution but not equal variance)

Supplementary Table S9. Comparison between light intensity (µmol-photons m^{-2} s^{-1}) on the outer and inner sides of the First Urasoe Breakwater in Naha Port, Japan, at LWL –3 m in each month.

Observed Year-Month	Station	Mean	Median	SD	*n*	Statistical test	Statistics	*P*	d.f.	Significance	Notes
2018-07	Outer side of the breakwater at LWL –3 m	136.95	146.52	57.26	31	Wilcoxon rank sum test	737.00	0.000		TRUE	**Maybe significant** (are not normal distribution and not equal variance)
2018-07	Inner side of the breakwater at LWL –3 m	91.77	101.47	42.67	31	Wilcoxon rank sum test	737.00	0.000		TRUE	**Maybe significant** (are not normal distribution and not equal variance)
2018-08	Outer side of the breakwater at LWL –3 m	134.89	129.35	77.88	31	Student's *t* -test	2.33	0.023	60	TRUE	**Maybe significant** (are normal distribution and equal variance)
2018-08	Inner side of the breakwater at LWL –3 m	97.45	102.78	44.11	31	Student's *t* -test	2.33	0.023	60	TRUE	**Maybe significant** (are normal distribution and equal variance)
2018-09	Outer side of the breakwater at LWL –3 m	116.82	109.09	64.01	28	Welch's *t* -test	0.50	0.620	53.73	FALSE	Not significant (are normal distribution but not equal variance)
2018-09	Inner side of the breakwater at LWL –3 m	108.57	109.97	59.60	28	Welch's *t* -test	0.50	0.620	53.73	FALSE	Not significant (are normal distribution but not equal variance)
2018-10	Outer side of the breakwater at LWL –3 m	31.27	32.07	14.88	22	Wilcoxon rank sum test	149.00	0.029		TRUE	**Maybe significant** (are not normal distribution and not equal variance)
2018-10	Inner side of the breakwater at LWL –3 m	73.25	62.62	55.76	22	Wilcoxon rank sum test	149.00	0.029		TRUE	**Maybe significant** (are not normal distribution and not equal variance)
2018-11	Outer side of the breakwater at LWL –3 m	31.75	31.26	7.13	25	Student's *t* -test	–7.57	0.000	48	TRUE	**Maybe significant** (are normal distribution and equal variance)
2018-11	Inner side of the breakwater at LWL –3 m	88.54	90.77	36.82	25	Student's *t* -test	–7.57	0.000	48	TRUE	**Maybe significant** (are normal distribution and equal variance)
2018-12	Outer side of the breakwater at LWL –3 m	24.55	24.88	11.88	22	Student's *t* -test	–3.01	0.005	41	TRUE	**Maybe significant** (are normal distribution and equal variance)
2018-12	Inner side of the breakwater at LWL –3 m	47.24	45.24	33.27	21	Student's *t* -test	–3.01	0.005	41	TRUE	**Maybe significant** (are normal distribution and equal variance)
2019-01	Outer side of the breakwater at LWL –3 m	25.48	27.52	10.20	23	Wilcoxon rank sum test	174.00	0.047		TRUE	**Maybe significant** (are not normal distribution and not equal variance)
2019-01	Inner side of the breakwater at LWL –3 m	53.26	35.19	39.80	23	Wilcoxon rank sum test	174.00	0.047		TRUE	**Maybe significant** (are not normal distribution and not equal variance)
2019-02	Outer side of the breakwater at LWL –3 m	31.72	32.31	15.84	26	Student's *t* -test	–3.37	0.001	50	TRUE	**Maybe significant** (are normal distribution and equal variance)
2019-02	Inner side of the breakwater at LWL –3 m	59.91	53.91	39.55	26	Student's *t* -test	–3.37	0.001	50	TRUE	**Maybe significant** (are normal distribution and equal variance)
2019-03	Outer side of the breakwater at LWL –3 m	29.89	31.19	9.08	18	Wilcoxon rank sum test	53.00	0.000		TRUE	**Maybe significant** (are not normal distribution and not equal variance)
2019-03	Inner side of the breakwater at LWL –3 m	77.46	71.73	43.75	18	Wilcoxon rank sum test	53.00	0.000		TRUE	**Maybe significant** (are not normal distribution and not equal variance)
2019-04	Outer side of the breakwater at LWL –3 m	84.54	90.46	36.93	23	Welch's *t* -test	0.68	0.500	42.56	FALSE	Not significant (are normal distribution but not equal variance)
2019-04	Inner side of the breakwater at LWL –3 m	76.34	71.14	44.49	23	Welch's *t* -test	0.68	0.500	42.56	FALSE	Not significant (are normal distribution but not equal variance)
2019-05	Outer side of the breakwater at LWL –3 m	105.18	105.33	52.28	29	Welch's *t* -test	2.71	0.009	50.06	TRUE	**Maybe significant** (are normal distribution but not equal variance)
2019-05	Inner side of the breakwater at LWL –3 m	73.06	67.77	36.51	29	Welch's *t* -test	2.71	0.009	50.06	TRUE	**Maybe significant** (are normal distribution but not equal variance)
2019-06	Outer side of the breakwater at LWL –3 m	94.91	77.49	55.42	24	Student's *t* -test	3.79	0.000	46	TRUE	**Maybe significant** (are normal distribution and equal variance)
2019-06	Inner side of the breakwater at LWL –3 m	45.47	39.68	31.74	24	Student's *t* -test	3.79	0.000	46	TRUE	**Maybe significant** (are normal distribution and equal variance)

Supplementary Table S10. Comparison between light intensity (µmol-photons m^{-2} s^{-1}) on the inner side of the First Urasoe Breakwater in Naha Port, Japan, at LWL –1 m and LWL –3 m in each month.

Observed Year-Month	Station	Mean	Median	SD	*n*	Statistical test	Statistics	*P*	d.f.	Significance	Notes
2018-07	Inner side of the breakwater at LWL –1 m	124.08	139.78	56.24	31	Welch's *t* -test	2.55	0.014	55.94	TRUE	**Maybe significant** (are normal distribution but not equal variance)
2018-07	Inner side of the breakwater at LWL –3 m	91.77	101.47	42.67	31	Welch's *t* -test	2.55	0.014	55.94	TRUE	**Maybe significant** (are normal distribution but not equal variance)
2018-08	Inner side of the breakwater at LWL –1 m	128.68	135.45	59.87	31	Welch's *t* -test	2.34	0.023	55.16	TRUE	**Maybe significant** (are normal distribution but not equal variance)
2018-08	Inner side of the breakwater at LWL –3 m	97.45	102.78	44.11	31	Welch's *t* -test	2.34	0.023	55.16	TRUE	**Maybe significant** (are normal distribution but not equal variance)
2018-09	Inner side of the breakwater at LWL –1 m	140.59	146.01	74.65	28	Welch's *t* -test	1.77	0.082	51.48	FALSE	Not significant (are normal distribution but not equal variance)
2018-09	Inner side of the breakwater at LWL –3 m	108.57	109.97	59.60	28	Welch's *t* -test	1.77	0.082	51.48	FALSE	Not significant (are normal distribution but not equal variance)
2018-10	Inner side of the breakwater at LWL –1 m	90.73	91.30	66.01	22	Wilcoxon rank sum test	279.00	0.395		FALSE	Not significant (are not normal distribution and not equal variance)
2018-10	Inner side of the breakwater at LWL –3 m	73.25	62.62	55.76	22	Wilcoxon rank sum test	279.00	0.395		FALSE	Not significant (are not normal distribution and not equal variance)
2018-11	Inner side of the breakwater at LWL –1 m	104.13	107.62	43.61	25	Welch's *t* -test	1.37	0.179	46.69	FALSE	Not significant (are normal distribution but not equal variance)
2018-11	Inner side of the breakwater at LWL –3 m	88.54	90.77	36.82	25	Welch's *t* -test	1.37	0.179	46.69	FALSE	Not significant (are normal distribution but not equal variance)
2018-12	Inner side of the breakwater at LWL –1 m	53.16	51.84	36.31	21	Welch's *t* -test	0.55	0.584	39.70	FALSE	Not significant (are normal distribution but not equal variance)
2018-12	Inner side of the breakwater at LWL –3 m	47.24	45.24	33.27	21	Welch's *t* -test	0.55	0.584	39.70	FALSE	Not significant (are normal distribution but not equal variance)
2019-01	Inner side of the breakwater at LWL –1 m	59.56	44.07	44.89	23	Wilcoxon rank sum test	300.00	0.446		FALSE	Not significant (are not normal distribution and not equal variance)
2019-01	Inner side of the breakwater at LWL –3 m	53.26	35.19	39.80	23	Wilcoxon rank sum test	300.00	0.446		FALSE	Not significant (are not normal distribution and not equal variance)
2019-02	Inner side of the breakwater at LWL –1 m	69.05	61.94	43.88	26	Welch's *t* -test	0.79	0.434	49.47	FALSE	Not significant (are normal distribution but not equal variance)
2019-02	Inner side of the breakwater at LWL –3 m	59.91	53.91	39.55	26	Welch's *t* -test	0.79	0.434	49.47	FALSE	Not significant (are normal distribution but not equal variance)
2019-03	Inner side of the breakwater at LWL –1 m	86.30	82.72	48.98	18	Wilcoxon rank sum test	190.00	0.389		FALSE	Not significant (are not normal distribution and not equal variance)
2019-03	Inner side of the breakwater at LWL –3 m	77.46	71.73	43.75	18	Wilcoxon rank sum test	190.00	0.389		FALSE	Not significant (are not normal distribution and not equal variance)
2019-04	Inner side of the breakwater at LWL –1 m	100.49	101.30	60.18	23	Welch's *t* -test	1.55	0.129	40.52	FALSE	Not significant (are normal distribution but not equal variance)
2019-04	Inner side of the breakwater at LWL –3 m	76.34	71.14	44.49	23	Welch's *t* -test	1.55	0.129	40.52	FALSE	Not significant (are normal distribution but not equal variance)
2019-05	Inner side of the breakwater at LWL –1 m	93.37	93.32	45.78	29	Welch's *t* -test	1.87	0.067	53.36	FALSE	Not significant (are normal distribution but not equal variance)
2019-05	Inner side of the breakwater at LWL –3 m	73.06	67.77	36.51	29	Welch's *t* -test	1.87	0.067	53.36	FALSE	Not significant (are normal distribution but not equal variance)
2019-06	Inner side of the breakwater at LWL –1 m	63.58	53.57	40.72	24	Welch's *t* -test	1.72	0.093	43.41	FALSE	Not significant (are normal distribution but not equal variance)
2019-06	Inner side of the breakwater at LWL –3 m	45.47	39.68	31.74	24	Welch's *t* -test	1.72	0.093	43.41	FALSE	Not significant (are normal distribution but not equal variance)

Supplementary Table S11. Comparison between significant wave height (m) on the outer and inner sides of the First Urasoe Breakwater in Naha Port, Japan, at LWL –3 m in each month.

Observed Year-Month	Station	Mean	Median	SD	*n*	Statistical test	Statistics	*P*	d.f.	Significance	Notes
2018-07	Outer side of the breakwater at LWL –3 m	0.42	0.39	0.25	31	Wilcoxon rank sum test	814	0.000		TRUE	**Maybe significant** (are not normal distribution and not equal variance)
2018-07	Inner side of the breakwater at LWL –3 m	0.17	0.14	0.11	31	Wilcoxon rank sum test	814	0.000		TRUE	**Maybe significant** (are not normal distribution and not equal variance)
2018-09	Outer side of the breakwater at LWL –3 m	0.77	0.49	0.92	28	Wilcoxon rank sum test	621	0.000		TRUE	**Maybe significant** (are not normal distribution and not equal variance)
2018-09	Inner side of the breakwater at LWL –3 m	0.29	0.18	0.31	28	Wilcoxon rank sum test	621	0.000		TRUE	**Maybe significant** (are not normal distribution and not equal variance)
2018-10	Outer side of the breakwater at LWL –3 m	0.93	0.77	0.59	22	Wilcoxon rank sum test	441	0.000		TRUE	**Maybe significant** (are not normal distribution and not equal variance)
2018-10	Inner side of the breakwater at LWL –3 m	0.30	0.20	0.22	22	Wilcoxon rank sum test	441	0.000		TRUE	**Maybe significant** (are not normal distribution and not equal variance)
2018-11	Outer side of the breakwater at LWL –3 m	0.34	0.22	0.25	25	Wilcoxon rank sum test	437	0.015		TRUE	**Maybe significant** (are not normal distribution and not equal variance)
2018-11	Inner side of the breakwater at LWL –3 m	0.16	0.16	0.08	25	Wilcoxon rank sum test	437	0.015		TRUE	**Maybe significant** (are not normal distribution and not equal variance)
2018-12	Outer side of the breakwater at LWL –3 m	0.82	0.52	0.76	22	Wilcoxon rank sum test	336	0.010		TRUE	**Maybe significant** (are not normal distribution and not equal variance)
2018-12	Inner side of the breakwater at LWL –3 m	0.24	0.15	0.20	21	Wilcoxon rank sum test	336	0.010		TRUE	**Maybe significant** (are not normal distribution and not equal variance)
2019-01	Outer side of the breakwater at LWL –3 m	0.83	0.74	0.50	23	Wilcoxon rank sum test	504	0.000		TRUE	**Maybe significant** (are not normal distribution and not equal variance)
2019-01	Inner side of the breakwater at LWL –3 m	0.24	0.21	0.12	23	Wilcoxon rank sum test	504	0.000		TRUE	**Maybe significant** (are not normal distribution and not equal variance)
2019-02	Outer side of the breakwater at LWL –3 m	0.80	0.67	0.51	26	Wilcoxon rank sum test	633	0.000		TRUE	**Maybe significant** (are not normal distribution and not equal variance)
2019-02	Inner side of the breakwater at LWL –3 m	0.24	0.21	0.12	26	Wilcoxon rank sum test	633	0.000		TRUE	**Maybe significant** (are not normal distribution and not equal variance)
2019-03	Outer side of the breakwater at LWL –3 m	0.76	0.59	0.37	15	Wilcoxon rank sum test	224	0.000		TRUE	**Maybe significant** (are not normal distribution and not equal variance)
2019-03	Inner side of the breakwater at LWL –3 m	0.21	0.17	0.09	15	Wilcoxon rank sum test	224	0.000		TRUE	**Maybe significant** (are not normal distribution and not equal variance)
2019-04	Outer side of the breakwater at LWL –3 m	0.41	0.22	0.35	23	Wilcoxon rank sum test	447	0.000		TRUE	**Maybe significant** (are not normal distribution and not equal variance)
2019-04	Inner side of the breakwater at LWL –3 m	0.13	0.09	0.08	23	Wilcoxon rank sum test	447	0.000		TRUE	**Maybe significant** (are not normal distribution and not equal variance)
2019-05	Outer side of the breakwater at LWL –3 m	0.35	0.22	0.31	29	Wilcoxon rank sum test	762	0.000		TRUE	**Maybe significant** (are not normal distribution and not equal variance)
2019-05	Inner side of the breakwater at LWL –3 m	0.11	0.08	0.08	29	Wilcoxon rank sum test	762	0.000		TRUE	**Maybe significant** (are not normal distribution and not equal variance)
2019-06	Outer side of the breakwater at LWL –3 m	0.31	0.25	0.22	24	Wilcoxon rank sum test	545	0.000		TRUE	**Maybe significant** (are not normal distribution and not equal variance)
2019-06	Inner side of the breakwater at LWL –3 m	0.12	0.11	0.05	24	Wilcoxon rank sum test	545	0.000		TRUE	**Maybe significant** (are not normal distribution and not equal variance)

Supplementary Table S12. Comparison between water temperature (˚C) on the outer side of the First Urasoe Breakwater in Naha Port, Japan, at LWL –1 m and LWL –3 m in each month.
Water temperature data at LWL –1 m outside the port in October 2018 are missing because the water temperature sensor was destroyed by a typhoon.

Observed Year-Month	Station	Mean	Median	SD	*n*	Statistical test	Statistics	*P*	d.f.	Significance	Notes
2018-07	Outer side of the breakwater at LWL –1 m	27.50	27.53	0.75	31	Welch's *t* -test	0.19	0.852	60.00	FALSE	Not significant (are normal distribution but not equal variance)
2018-07	Outer side the breakwater at LWL –3 m	27.46	27.47	0.75	31	Welch's *t* -test	0.19	0.852	60.00	FALSE	Not significant (are normal distribution but not equal variance)
2018-08	Outer side of the breakwater at LWL –1 m	28.44	28.52	0.40	31	Welch's *t* -test	1.05	0.299	48.60	FALSE	Not significant (are normal distribution but not equal variance)
2018-08	Outer side the breakwater at LWL –3 m	28.32	28.32	0.42	24	Welch's *t* -test	1.05	0.299	48.60	FALSE	Not significant (are normal distribution but not equal variance)
2018-09	Outer side of the breakwater at LWL –1 m	28.48	28.51	0.41	28	Welch's *t* -test	0.28	0.778	53.92	FALSE	Not significant (are normal distribution but not equal variance)
2018-09	Outer side the breakwater at LWL –3 m	28.45	28.50	0.42	28	Welch's *t* -test	0.28	0.778	53.92	FALSE	Not significant (are normal distribution but not equal variance)
2018-10	Outer side of the breakwater at LWL –1 m				0					FALSE	Data size must be larger than 3. Actural x: 0, y: 22
2018-10	Outer side the breakwater at LWL –3 m	25.59	25.45	0.39	22					FALSE	Data size must be larger than 3. Actural x: 0, y: 22
2018-11	Outer side of the breakwater at LWL –1 m	26.03	26.21	0.63	25	Wilcoxon rank sum test	313.00	1.000		FALSE	Not significant (are not normal distribution and not equal variance)
2018-11	Outer side the breakwater at LWL –3 m	26.03	26.21	0.62	25	Wilcoxon rank sum test	313.00	1.000		FALSE	Not significant (are not normal distribution and not equal variance)
2018-12	Outer side of the breakwater at LWL –1 m	25.01	25.07	0.53	27	Welch's *t* -test	0.49	0.629	45.31	FALSE	Not significant (are normal distribution but not equal variance)
2018-12	Outer side the breakwater at LWL –3 m	24.94	25.04	0.52	22	Welch's *t* -test	0.49	0.629	45.31	FALSE	Not significant (are normal distribution but not equal variance)
2019-01	Outer side of the breakwater at LWL –1 m	23.03	23.05	0.63	23	Welch's *t* -test	–0.15	0.883	43.98	FALSE	Not significant (are normal distribution but not equal variance)
2019-01	Outer side the breakwater at LWL –3 m	23.06	23.08	0.62	23	Welch's *t* -test	–0.15	0.883	43.98	FALSE	Not significant (are normal distribution but not equal variance)
2019-02	Outer side of the breakwater at LWL –1 m	22.84	22.85	0.29	26	Welch's *t* -test	–0.04	0.965	49.98	FALSE	Not significant (are normal distribution but not equal variance)
2019-02	Outer side the breakwater at LWL –3 m	22.84	22.86	0.28	26	Welch's *t* -test	–0.04	0.965	49.98	FALSE	Not significant (are normal distribution but not equal variance)
2019-03	Outer side of the breakwater at LWL –1 m	22.87	22.93	0.26	18	Wilcoxon rank sum test	157.00	0.888		FALSE	Not significant (are not normal distribution and not equal variance)
2019-03	Outer side the breakwater at LWL –3 m	22.87	22.94	0.26	18	Wilcoxon rank sum test	157.00	0.888		FALSE	Not significant (are not normal distribution and not equal variance)
2019-04	Outer side of the breakwater at LWL –1 m	23.47	23.44	0.60	23	Welch's *t* -test	0.19	0.854	43.96	FALSE	Not significant (are normal distribution but not equal variance)
2019-04	Outer side the breakwater at LWL –3 m	23.44	23.38	0.58	23	Welch's *t* -test	0.19	0.854	43.96	FALSE	Not significant (are normal distribution but not equal variance)
2019-05	Outer side of the breakwater at LWL –1 m	25.20	25.26	0.66	29	Welch's *t* -test	0.12	0.903	56.00	FALSE	Not significant (are normal distribution but not equal variance)
2019-05	Outer side the breakwater at LWL –3 m	25.18	25.28	0.65	29	Welch's *t* -test	0.12	0.903	56.00	FALSE	Not significant (are normal distribution but not equal variance)
2019-06	Outer side of the breakwater at LWL –1 m	25.60	25.56	0.26	24	Wilcoxon rank sum test	326.00	0.443		FALSE	Not significant (are not normal distribution and not equal variance)
2019-06	Outer side the breakwater at LWL –3 m	25.56	25.52	0.26	24	Wilcoxon rank sum test	326.00	0.443		FALSE	Not significant (are not normal distribution and not equal variance)

Supplementary Table S13. Comparison between water temperature (˚C) on the outer and inner sides of the First Urasoe Breakwater in Naha Port, Japan, at LWL –1 m in each month.
Water temperature data at LWL –1 m outside the port in October 2018 are missing because the water temperature sensor was destroyed by a typhoon.

Observed Year-Month	Station	Mean	Median	SD	*n*	Statistical test	Statistics	*P*	d.f.	Significance	Notes
2018-07	Outer side of the breakwater at LWL –1 m	27.50	27.53	0.75	31	Wilcoxon rank sum test	511.00	0.675		FALSE	Not significant (are not normal distribution and not equal variance)
2018-07	Inner side of the breakwater at LWL –1 m	27.45	27.44	0.66	31	Wilcoxon rank sum test	511.00	0.675		FALSE	Not significant (are not normal distribution and not equal variance)
2018-08	Outer side of the breakwater at LWL –1 m	28.44	28.52	0.40	31	Welch's *t* -test	0.65	0.517	59.26	FALSE	Not significant (are normal distribution but not equal variance)
2018-08	Inner side of the breakwater at LWL –1 m	28.37	28.47	0.36	31	Welch's *t* -test	0.65	0.517	59.26	FALSE	Not significant (are normal distribution but not equal variance)
2018-09	Outer side of the breakwater at LWL –1 m	28.48	28.51	0.41	28	Welch's *t* -test	0.39	0.701	53.98	FALSE	Not significant (are normal distribution but not equal variance)
2018-09	Inner side of the breakwater at LWL –1 m	28.44	28.52	0.40	28	Welch's *t* -test	0.39	0.701	53.98	FALSE	Not significant (are normal distribution but not equal variance)
2018-10	Outer side of the breakwater at LWL –1 m				0					FALSE	Data size must be larger than 3. Actural x: 0, y: 22
2018-10	Inner side of the breakwater at LWL –1 m	25.58	25.42	0.43	22					FALSE	Data size must be larger than 3. Actural x: 0, y: 22
2018-11	Outer side of the breakwater at LWL –1 m	26.03	26.21	0.63	25	Wilcoxon rank sum test	341.00	0.590		FALSE	Not significant (are not normal distribution and not equal variance)
2018-11	Inner side of the breakwater at LWL –1 m	25.96	26.19	0.60	25	Wilcoxon rank sum test	341.00	0.590		FALSE	Not significant (are not normal distribution and not equal variance)
2018-12	Outer side of the breakwater at LWL –1 m	25.01	25.07	0.53	27	Welch's *t* -test	1.72	0.092	40.77	FALSE	Not significant (are normal distribution but not equal variance)
2018-12	Inner side of the breakwater at LWL –1 m	24.73	24.84	0.59	21	Welch's *t* -test	1.72	0.092	40.77	FALSE	Not significant (are normal distribution but not equal variance)
2019-01	Outer side of the breakwater at LWL –1 m	23.03	23.05	0.63	23	Welch's *t* -test	0.55	0.588	44.00	FALSE	Not significant (are normal distribution but not equal variance)
2019-01	Inner side of the breakwater at LWL –1 m	22.93	22.93	0.63	23	Welch's *t* -test	0.55	0.588	44.00	FALSE	Not significant (are normal distribution but not equal variance)
2019-02	Outer side of the breakwater at LWL –1 m	22.84	22.85	0.29	26	Welch's *t* -test	0.45	0.656	49.91	FALSE	Not significant (are normal distribution but not equal variance)
2019-02	Inner side of the breakwater at LWL –1 m	22.80	22.81	0.30	26	Welch's *t* -test	0.45	0.656	49.91	FALSE	Not significant (are normal distribution but not equal variance)
2019-03	Outer side of the breakwater at LWL –1 m	22.87	22.93	0.26	18	Wilcoxon rank sum test	183.00	0.521		FALSE	Not significant (are not normal distribution and not equal variance)
2019-03	Inner side of the breakwater at LWL –1 m	22.83	22.90	0.28	18	Wilcoxon rank sum test	183.00	0.521		FALSE	Not significant (are not normal distribution and not equal variance)
2019-04	Outer side of the breakwater at LWL –1 m	23.47	23.44	0.60	23	Welch's *t* -test	–0.05	0.963	44.00	FALSE	Not significant (are normal distribution but not equal variance)
2019-04	Inner side of the breakwater at LWL –1 m	23.48	23.40	0.60	23	Welch's *t* -test	–0.05	0.963	44.00	FALSE	Not significant (are normal distribution but not equal variance)
2019-05	Outer side of the breakwater at LWL –1 m	25.20	25.26	0.66	29	Welch's *t* -test	0.13	0.900	55.82	FALSE	Not significant (are normal distribution but not equal variance)
2019-05	Inner side of the breakwater at LWL –1 m	25.18	25.28	0.62	29	Welch's *t* -test	0.13	0.900	55.82	FALSE	Not significant (are normal distribution but not equal variance)
2019-06	Outer side of the breakwater at LWL –1 m	25.60	25.56	0.26	24	Wilcoxon rank sum test	245.00	0.384		FALSE	Not significant (are not normal distribution and not equal variance)
2019-06	Inner side of the breakwater at LWL –1 m	25.67	25.60	0.26	24	Wilcoxon rank sum test	245.00	0.384		FALSE	Not significant (are not normal distribution and not equal variance)

Supplementary Table S14. Comparison between water temperature (˚C) on the outer and inner sides of the First Urasoe Breakwater in Naha Port, Japan, at LWL –3 m in each month.

Observed Year-Month	Station	Mean	Median	SD	*n*	Statistical test	Statistics	*P*	d.f.	Significance	Notes
2018-07	Outer side the breakwater at LWL –3 m	27.46	27.47	0.75	31	Wilcoxon rank sum test	508.00	0.706		FALSE	Not significant (are not normal distribution and not equal variance)
2018-07	Inner side of the breakwater at LWL –3 m	27.43	27.42	0.65	31	Wilcoxon rank sum test	508.00	0.706		FALSE	Not significant (are not normal distribution and not equal variance)
2018-08	Outer side the breakwater at LWL –3 m	28.32	28.32	0.42	24	Wilcoxon rank sum test	349.00	0.705		FALSE	Not significant (are not normal distribution and not equal variance)
2018-08	Inner side of the breakwater at LWL –3 m	28.35	28.45	0.34	31	Wilcoxon rank sum test	349.00	0.705		FALSE	Not significant (are not normal distribution and not equal variance)
2018-09	Outer side the breakwater at LWL –3 m	28.45	28.50	0.42	28	Welch's *t* -test	0.18	0.857	53.74	FALSE	Not significant (are normal distribution but not equal variance)
2018-09	Inner side of the breakwater at LWL –3 m	28.43	28.51	0.39	28	Welch's *t* -test	0.18	0.857	53.74	FALSE	Not significant (are normal distribution but not equal variance)
2018-10	Outer side the breakwater at LWL –3 m	25.59	25.45	0.39	22	Wilcoxon rank sum test	253.00	0.807		FALSE	Not significant (are not normal distribution and not equal variance)
2018-10	Inner side of the breakwater at LWL –3 m	25.58	25.41	0.43	22	Wilcoxon rank sum test	253.00	0.807		FALSE	Not significant (are not normal distribution and not equal variance)
2018-11	Outer side the breakwater at LWL –3 m	26.03	26.21	0.62	25	Wilcoxon rank sum test	340.00	0.603		FALSE	Not significant (are not normal distribution and not equal variance)
2018-11	Inner side of the breakwater at LWL –3 m	25.96	26.19	0.60	25	Wilcoxon rank sum test	340.00	0.603		FALSE	Not significant (are not normal distribution and not equal variance)
2018-12	Outer side the breakwater at LWL –3 m	24.94	25.04	0.52	22	Welch's *t* -test	1.22	0.229	39.87	FALSE	Not significant (are normal distribution but not equal variance)
2018-12	Inner side of the breakwater at LWL –3 m	24.73	24.85	0.59	21	Welch's *t* -test	1.22	0.229	39.87	FALSE	Not significant (are normal distribution but not equal variance)
2019-01	Outer side the breakwater at LWL –3 m	23.06	23.08	0.62	23	Welch's *t* -test	0.68	0.501	43.97	FALSE	Not significant (are normal distribution but not equal variance)
2019-01	Inner side of the breakwater at LWL –3 m	22.93	22.98	0.63	23	Welch's *t* -test	0.68	0.501	43.97	FALSE	Not significant (are normal distribution but not equal variance)
2019-02	Outer side the breakwater at LWL –3 m	22.84	22.86	0.28	26	Welch's *t* -test	0.46	0.647	49.74	FALSE	Not significant (are normal distribution but not equal variance)
2019-02	Inner side of the breakwater at LWL –3 m	22.80	22.81	0.30	26	Welch's *t* -test	0.46	0.647	49.74	FALSE	Not significant (are normal distribution but not equal variance)
2019-03	Outer side the breakwater at LWL –3 m	22.87	22.94	0.26	18	Wilcoxon rank sum test	179.00	0.606		FALSE	Not significant (are not normal distribution and not equal variance)
2019-03	Inner side of the breakwater at LWL –3 m	22.84	22.90	0.28	18	Wilcoxon rank sum test	179.00	0.606		FALSE	Not significant (are not normal distribution and not equal variance)
2019-04	Outer side the breakwater at LWL –3 m	23.44	23.38	0.58	23	Welch's *t* -test	–0.21	0.835	43.96	FALSE	Not significant (are normal distribution but not equal variance)
2019-04	Inner side of the breakwater at LWL –3 m	23.47	23.40	0.60	23	Welch's *t* -test	–0.21	0.835	43.96	FALSE	Not significant (are normal distribution but not equal variance)
2019-05	Outer side the breakwater at LWL –3 m	25.18	25.28	0.65	29	Welch's *t* -test	0.04	0.965	55.78	FALSE	Not significant (are normal distribution but not equal variance)
2019-05	Inner side of the breakwater at LWL –3 m	25.17	25.27	0.61	29	Welch's *t* -test	0.04	0.965	55.78	FALSE	Not significant (are normal distribution but not equal variance)
2019-06	Outer side the breakwater at LWL –3 m	25.56	25.52	0.26	24	Welch's *t* -test	–1.05	0.298	45.94	FALSE	Not significant (are normal distribution but not equal variance)
2019-06	Inner side of the breakwater at LWL –3 m	25.64	25.59	0.25	24	Welch's *t* -test	–1.05	0.298	45.94	FALSE	Not significant (are normal distribution but not equal variance)

Supplementary Table S15. Comparison between water temperature (˚C) on the inner side of the First Urasoe Breakwater in Naha Port, Japan, at LWL –1 m and LWL –3 m in each month.

Observed Year-Month	Station	Mean	Median	SD	*n*	Statistical test	Statistics	*P*	d.f.	Significance	Notes
2018-07	Inner side of the breakwater at LWL –1 m	27.45	27.44	0.66	31	Wilcoxon rank sum test	501.00	0.780		FALSE	Not significant (are not normal distribution and not equal variance)
2018-07	Inner side of the breakwater at LWL –3 m	27.43	27.42	0.65	31	Wilcoxon rank sum test	501.00	0.780		FALSE	Not significant (are not normal distribution and not equal variance)
2018-08	Inner side of the breakwater at LWL –1 m	28.37	28.47	0.36	31	Wilcoxon rank sum test	506.00	0.727		FALSE	Not significant (are not normal distribution and not equal variance)
2018-08	Inner side of the breakwater at LWL –3 m	28.35	28.45	0.34	31	Wilcoxon rank sum test	506.00	0.727		FALSE	Not significant (are not normal distribution and not equal variance)
2018-09	Inner side of the breakwater at LWL –1 m	28.44	28.52	0.40	28	Welch's *t* -test	0.09	0.927	53.99	FALSE	Not significant (are normal distribution but not equal variance)
2018-09	Inner side of the breakwater at LWL –3 m	28.43	28.51	0.39	28	Welch's *t* -test	0.09	0.927	53.99	FALSE	Not significant (are normal distribution but not equal variance)
2018-10	Inner side of the breakwater at LWL –1 m	25.58	25.42	0.43	22	Wilcoxon rank sum test	245.00	0.954		FALSE	Not significant (are not normal distribution and not equal variance)
2018-10	Inner side of the breakwater at LWL –3 m	25.58	25.41	0.43	22	Wilcoxon rank sum test	245.00	0.954		FALSE	Not significant (are not normal distribution and not equal variance)
2018-11	Inner side of the breakwater at LWL –1 m	25.96	26.19	0.60	25	Wilcoxon rank sum test	300.00	0.818		FALSE	Not significant (are not normal distribution and not equal variance)
2018-11	Inner side of the breakwater at LWL –3 m	25.96	26.19	0.60	25	Wilcoxon rank sum test	300.00	0.818		FALSE	Not significant (are not normal distribution and not equal variance)
2018-12	Inner side of the breakwater at LWL –1 m	24.73	24.84	0.59	21	Welch's *t* -test	–0.01	0.994	40.00	FALSE	Not significant (are normal distribution but not equal variance)
2018-12	Inner side of the breakwater at LWL –3 m	24.73	24.85	0.59	21	Welch's *t* -test	–0.01	0.994	40.00	FALSE	Not significant (are normal distribution but not equal variance)
2019-01	Inner side of the breakwater at LWL –1 m	22.93	22.93	0.63	23	Welch's *t* -test	–0.02	0.986	44.00	FALSE	Not significant (are normal distribution but not equal variance)
2019-01	Inner side of the breakwater at LWL –3 m	22.93	22.98	0.63	23	Welch's *t* -test	–0.02	0.986	44.00	FALSE	Not significant (are normal distribution but not equal variance)
2019-02	Inner side of the breakwater at LWL –1 m	22.80	22.81	0.30	26	Welch's *t* -test	–0.03	0.974	50.00	FALSE	Not significant (are normal distribution but not equal variance)
2019-02	Inner side of the breakwater at LWL –3 m	22.80	22.81	0.30	26	Welch's *t* -test	–0.03	0.974	50.00	FALSE	Not significant (are normal distribution but not equal variance)
2019-03	Inner side of the breakwater at LWL –1 m	22.83	22.90	0.28	18	Welch's *t* -test	–0.04	0.968	34.00	FALSE	Not significant (are normal distribution but not equal variance)
2019-03	Inner side of the breakwater at LWL –3 m	22.84	22.90	0.28	18	Welch's *t* -test	–0.04	0.968	34.00	FALSE	Not significant (are normal distribution but not equal variance)
2019-04	Inner side of the breakwater at LWL –1 m	23.48	23.40	0.60	23	Welch's *t* -test	0.02	0.982	44.00	FALSE	Not significant (are normal distribution but not equal variance)
2019-04	Inner side of the breakwater at LWL –3 m	23.47	23.40	0.60	23	Welch's *t* -test	0.02	0.982	44.00	FALSE	Not significant (are normal distribution but not equal variance)
2019-05	Inner side of the breakwater at LWL –1 m	25.18	25.28	0.62	29	Welch's *t* -test	0.04	0.965	55.99	FALSE	Not significant (are normal distribution but not equal variance)
2019-05	Inner side of the breakwater at LWL –3 m	25.17	25.27	0.61	29	Welch's *t* -test	0.04	0.965	55.99	FALSE	Not significant (are normal distribution but not equal variance)
2019-06	Inner side of the breakwater at LWL –1 m	25.67	25.60	0.26	24	Welch's *t* -test	0.36	0.718	45.95	FALSE	Not significant (are normal distribution but not equal variance)
2019-06	Inner side of the breakwater at LWL –3 m	25.64	25.59	0.25	24	Welch's *t* -test	0.36	0.718	45.95	FALSE	Not significant (are normal distribution but not equal variance)

Supplementary Table S16. Comparison between water transparency (m) over the First Shinko Breakwater and Urasoe reef in Naha Port, Japan.

Site	Mean	Median	SD	*n*	Statistical test	Statistics	*P*	d.f.	Significance	Notes
Offshore side of the First Shinko breakwater	10.43	10.00	3.89	190	Student's *t* -test	–1.51	0.131	280	FALSE	Not significant (are normal distribution and equal variance)
Offshore side of the Urasoe reef	11.16	11.80	3.20	91	Student's *t* -test	–1.51	0.131	280	FALSE	Not significant (are normal distribution and equal variance)
Offshore side of the First Shinko breakwater	10.43	10.00	3.89	190	Welch's *t* -test	–0.82	0.410	374.81	FALSE	Not significant (are normal distribution but not equal variance)
Inshore side of the First Shinko breakwater	10.75	10.00	3.55	190	Welch's *t* -test	–0.82	0.410	374.81	FALSE	Not significant (are normal distribution but not equal variance)

Supplementary Table S17. Comparisons between alr-transformed coral cover at different sedimentation levels (ranks) on breakwaters and natural reefs in Naha Port, Japan.

Site	Taxon	Sedimentation rank[a]	Mean	Median	SD	*n*	Statistical test	Statistics	*P*	Significance	Notes
Breakwaters	All hard corals	I	–2.06	–1.39	2.09	593	Wilcoxon rank sum test	5790.00	0.539	FALSE	Not significant (are not normal distribution and not equal variance)
Breakwaters	All hard corals	II	–2.52	–2.20	2.24	18	Wilcoxon rank sum test	5790.00	0.539	FALSE	Not significant (are not normal distribution and not equal variance)
Breakwaters	*Acropora*	I	–3.37	–3.48	2.39	593	Wilcoxon rank sum test	7565.00	0.003	TRUE	**Maybe significant** (are not normal distribution and not equal variance)
Breakwaters	*Acropora*	II	–5.46	–5.62	0.89	18	Wilcoxon rank sum test	7565.00	0.003	TRUE	**Maybe significant** (are not normal distribution and not equal variance)
Breakwaters	*Pocillopora*	I	–3.98	–5.29	2.13	593	Wilcoxon rank sum test	7409.00	0.005	TRUE	**Maybe significant** (are not normal distribution and not equal variance)
Breakwaters	*Pocillopora*	II	–5.76	–5.87	0.38	18	Wilcoxon rank sum test	7409.00	0.005	TRUE	**Maybe significant** (are not normal distribution and not equal variance)
Breakwaters	*Montipora*	I	–5.28	–5.69	1.17	593	Wilcoxon rank sum test	6242.50	0.220	FALSE	Not significant (are not normal distribution and not equal variance)
Breakwaters	*Montipora*	II	–5.71	–5.87	0.32	18	Wilcoxon rank sum test	6242.50	0.220	FALSE	Not significant (are not normal distribution and not equal variance)
Breakwaters	*Porites*	I	–5.44	–5.70	0.90	593	Wilcoxon rank sum test	5065.50	0.713	FALSE	Not significant (are not normal distribution and not equal variance)
Breakwaters	*Porites*	II	–3.73	–5.87	2.86	18	Wilcoxon rank sum test	5065.50	0.713	FALSE	Not significant (are not normal distribution and not equal variance)
Breakwaters	Faviidae	I	–5.14	–5.63	1.17	593	Wilcoxon rank sum test	6135.50	0.279	FALSE	Not significant (are not normal distribution and not equal variance)
Breakwaters	Faviidae	II	–5.68	–5.73	0.36	18	Wilcoxon rank sum test	6135.50	0.279	FALSE	Not significant (are not normal distribution and not equal variance)
Breakwaters	*Millepora*	I	–5.25	–5.70	1.28	593	Wilcoxon rank sum test	4538.00	0.279	FALSE	Not significant (are not normal distribution and not equal variance)
Breakwaters	*Millepora*	II	–4.90	–5.55	1.46	18	Wilcoxon rank sum test	4538.00	0.279	FALSE	Not significant (are not normal distribution and not equal variance)
Breakwaters	Other hard corals	I	–5.54	–5.70	0.64	593	Wilcoxon rank sum test	6045.00	0.337	FALSE	Not significant (are not normal distribution and not equal variance)
Breakwaters	Other hard corals	II	–5.71	–5.87	0.32	18	Wilcoxon rank sum test	6045.00	0.337	FALSE	Not significant (are not normal distribution and not equal variance)
Naural reefs	All hard corals	I	–2.70	–2.20	1.48	997	Wilcoxon rank sum test	77096.50	0.291	FALSE	Not significant (are not normal distribution and not equal variance)
Naural reefs	All hard corals	II	–2.46	–2.20	1.54	163	Wilcoxon rank sum test	77096.50	0.291	FALSE	Not significant (are not normal distribution and not equal variance)
Naural reefs	*Acropora*	I	–5.18	–5.70	1.22	997	Wilcoxon rank sum test	101769.00	0.000	TRUE	**Maybe significant** (are not normal distribution and not equal variance)
Naural reefs	*Acropora*	II	–5.78	–5.88	0.61	163	Wilcoxon rank sum test	101769.00	0.000	TRUE	**Maybe significant** (are not normal distribution and not equal variance)
Naural reefs	*Pocillopora*	I	–5.17	–5.72	1.18	997	Wilcoxon rank sum test	98846.50	0.000	TRUE	**Maybe significant** (are not normal distribution and not equal variance)
Naural reefs	*Pocillopora*	II	–5.73	–5.88	0.58	163	Wilcoxon rank sum test	98846.50	0.000	TRUE	**Maybe significant** (are not normal distribution and not equal variance)
Naural reefs	*Montipora*	I	–5.64	–5.88	1.01	997	Wilcoxon rank sum test	78468.50	0.482	FALSE	Not significant (are not normal distribution and not equal variance)
Naural reefs	*Montipora*	II	–5.07	–5.88	2.05	163	Wilcoxon rank sum test	78468.50	0.482	FALSE	Not significant (are not normal distribution and not equal variance)
Naural reefs	*Porites*	I	–5.12	–5.42	1.31	997	Wilcoxon rank sum test	52948.00	0.000	TRUE	**Maybe significant** (are not normal distribution and not equal variance)
Naural reefs	*Porites*	II	–4.18	–4.20	1.53	163	Wilcoxon rank sum test	52948.00	0.000	TRUE	**Maybe significant** (are not normal distribution and not equal variance)
Naural reefs	Faviidae	I	–3.82	–3.78	1.52	997	Wilcoxon rank sum test	95592.00	0.000	TRUE	**Maybe significant** (are not normal distribution and not equal variance)
Naural reefs	Faviidae	II	–4.24	–4.11	1.13	163	Wilcoxon rank sum test	95592.00	0.000	TRUE	**Maybe significant** (are not normal distribution and not equal variance)
Naural reefs	*Millepora*	I	–5.54	–5.88	1.15	997	Wilcoxon rank sum test	85580.00	0.275	FALSE	Not significant (are not normal distribution and not equal variance)
Naural reefs	*Millepora*	II	–5.86	–5.88	0.57	163	Wilcoxon rank sum test	85580.00	0.275	FALSE	Not significant (are not normal distribution and not equal variance)
Naural reefs	Other hard corals	I	–4.54	–4.55	1.38	997	Wilcoxon rank sum test	77485.50	0.342	FALSE	Not significant (are not normal distribution and not equal variance)
Naural reefs	Other hard corals	II	–4.44	–4.41	1.10	163	Wilcoxon rank sum test	77485.50	0.342	FALSE	Not significant (are not normal distribution and not equal variance)

[a]I, no turbidity even after tapping the seafloor; II, turbidity when the seafloor is tapped

Supplementary Table S18. Comparison between the maximum colony diameter (cm) for each coral taxon on breakwaters and natural reefs in Naha Port, Japan.

Taxon	Site	Mean	Median	SD	*n*	Statistical test	Statistics	*P*	d.f.	Significance	Notes
Acropora	Breakwaters	15.30	13.00	12.30	1275	Student's *t* -test	–1.37	0.171	1459	FALSE	Not significant (are normal distribution and equal variance)
Acropora	Natural reefs	16.70	8.50	17.09	186	Student's *t* -test	–1.37	0.171	1459	FALSE	Not significant (are normal distribution and equal variance)
Pocillopora	Breakwaters	14.17	12.50	9.79	1062	Student's *t* -test	–3.77	0.000	1237	TRUE	**Maybe significant** (are normal distribution and equal variance)
Pocillopora	Natural reefs	17.21	17.00	10.64	177	Student's *t* -test	–3.77	0.000	1237	TRUE	**Maybe significant** (are normal distribution and equal variance)
Montipora	Breakwaters	13.05	11.00	8.59	171	Student's *t* -test	–1.39	0.165	244	FALSE	Not significant (are normal distribution and equal variance)
Montipora	Natural reefs	14.90	12.00	11.51	75	Student's *t* -test	–1.39	0.165	244	FALSE	Not significant (are normal distribution and equal variance)
Porites	Breakwaters	10.02	7.00	8.61	392	Student's *t* -test	–9.70	0.000	623	TRUE	**Maybe significant** (are normal distribution and equal variance)
Porites	Natural reefs	24.02	15.00	26.33	233	Student's *t* -test	–9.70	0.000	623	TRUE	**Maybe significant** (are normal distribution and equal variance)
Faviidae	Breakwaters	6.95	5.00	5.19	346	Student's *t* -test	–11.71	0.000	650	TRUE	**Maybe significant** (are normal distribution and equal variance)
Faviidae	Natural reefs	22.27	14.00	23.71	306	Student's *t* -test	–11.71	0.000	650	TRUE	**Maybe significant** (are normal distribution and equal variance)
Millepora	Breakwaters	19.45	15.00	14.87	219	Student's *t* -test	–0.58	0.566	294	FALSE	Not significant (are normal distribution and equal variance)
Millepora	Natural reefs	20.63	17.00	16.93	77	Student's *t* -test	–0.58	0.566	294	FALSE	Not significant (are normal distribution and equal variance)
Other hard corals	Breakwaters	6.85	6.00	4.18	102	Student's *t* -test	–6.34	0.000	325	TRUE	**Maybe significant** (are normal distribution and equal variance)
Other hard corals	Natural reefs	10.96	10.00	5.90	225	Student's *t* -test	–6.34	0.000	325	TRUE	**Maybe significant** (are normal distribution and equal variance)

Supplementary Table S19. Number of data of each survey method of each year.

Fiscal research year	Substrate	Belt transect	Manta	Quadrat	Snorkel	Spot check
1989	Break waters	0	0	82	0	0
1990	Break waters	0	0	207	0	0
1991	Break waters	0	0	260	0	0
1992	Break waters	0	0	30	0	0
1993	Break waters	0	0	30	0	0
1994	Break waters	0	0	67	66	0
	Natural reefs	0	0	12	0	0
1995	Break waters	0	0	70	0	0
1996	Break waters	0	0	30	0	0
	Natural reefs	33	0	18	0	0
2000	Break waters	0	0	66	0	0
	Natural reefs	0	0	9	0	0
2001	Break waters	0	0	84	0	0
	Natural reefs	61	0	12	0	0
2002	Break waters	0	0	84	0	0
	Natural reefs	61	0	12	0	0
2003	Break waters	0	0	84	75	0
	Natural reefs	61	0	12	0	0
2004	Break waters	0	0	84	0	0
	Natural reefs	61	0	12	0	0
2005	Break waters	0	0	84	0	0
	Natural reefs	61	0	12	0	0
2006	Natural reefs	78	128	15	0	17
2007	Break waters	0	0	84	0	0
	Natural reefs	78	0	15	0	0

2008	Natural reefs	78	0	15	0	0
2009	Break waters	0	0	84	1	0
	Natural reefs	78	0	15	0	0
2010	Natural reefs	78	59	15	67	17
2011	Break waters	0	0	84	0	0
	Natural reefs	78	0	31	0	0
2012	Natural reefs	78	0	31	0	0
2013	Break waters	0	0	84	0	0
	Natural reefs	78	0	31	0	0
2014	Break waters	0	0	0	79	0
	Natural reefs	78	0	15	0	0
2015	Break waters	0	0	84	0	0
	Natural reefs	78	99	31	31	17
2016	Natural reefs	78	0	27	0	0
2017	Break waters	0	0	84	0	0
	Natural reefs	78	0	27	0	0
2018	Natural reefs	78	0	27	0	0

Supplementary Table S20. Number of data points (*n*) for coral cover by taxon on the outer side of breakwaters in Naha Port, Japan, for each fiscal research year.

Fiscal research year	Location	All hard corals (*n*)	*Acropora* (*n*)	*Pocillopora* (*n*)	*Montipora* (*n*)	*Porites* (*n*)	Faviidae (*n*)	*Millepora* (*n*)	Other hard corals (*n*)
1989	First Shinko Breakwater	82	82	82	82	82	82	82	82
1990	First Shinko Breakwater	207	207	207	207	207	207	207	207
1991	First Shinko Breakwater	209	209	209	209	209	209	209	209
1991	First Urasoe Breakwater	21	21	21	21	21	21	21	21
1991	Naha Breakwater	30	30	30	30	30	30	30	30
1992	First Shinko Breakwater	30	30	30	30	30	30	30	30
1993	First Shinko Breakwater	30	30	30	30	30	30	30	30
1994	First Shinko Breakwater	99	99	99	99	99	99	99	99
1994	First Urasoe Breakwater	20	20	20	20	20	20	20	20
1994	Naha Breakwater	14	14	14	14	14	14	14	14
1995	First Shinko Breakwater	30	30	30	30	30	30	30	30
1995	First Urasoe Breakwater	20	20	20	20	20	20	20	20
1995	Naha Breakwater	20	20	20	20	20	20	20	20
1996	First Shinko Breakwater	30	30	30	30	30	30	30	30
2000	First Shinko Breakwater	30	30	30	30	30	30	30	30
2000	Naha Breakwater	36	36	36	36	36	36	36	36
2001	First Shinko Breakwater	30	30	30	30	30	30	30	30
2001	First Urasoe Breakwater	18	18	18	18	18	18	18	18
2001	Naha Breakwater	36	36	36	36	36	36	36	36
2002	First Shinko Breakwater	30	30	30	30	30	30	30	30
2002	First Urasoe Breakwater	18	18	18	18	18	18	18	18
2002	Naha Breakwater	36	36	36	36	36	36	36	36
2003	First Shinko Breakwater	63	63	63	63	63	63	63	63
2003	First Urasoe Breakwater	40	40	40	40	40	40	40	40
2003	Naha Breakwater	56	56	56	56	56	56	56	56
2004	First Shinko Breakwater	30	30	30	30	30	30	30	30
2004	First Urasoe Breakwater	18	18	18	18	18	18	18	18
2004	Naha Breakwater	36	36	36	36	36	36	36	36
2005	First Shinko Breakwater	30	30	30	30	30	30	30	30
2005	First Urasoe Breakwater	18	18	18	18	18	18	18	18
2005	Naha Breakwater	36	36	36	36	36	36	36	36
2007	First Shinko Breakwater	30	30	30	30	30	30	30	30
2007	First Urasoe Breakwater	18	18	18	18	18	18	18	18
2007	Naha Breakwater	36	36	36	36	36	36	36	36
2009	First Shinko Breakwater	30	30	30	30	30	30	30	30
2009	First Urasoe Breakwater	18	18	18	18	18	18	18	18
2009	Naha Breakwater	37	37	37	37	37	37	37	37
2011	First Shinko Breakwater	30	30	30	30	30	30	30	30
2011	First Urasoe Breakwater	18	18	18	18	18	18	18	18
2011	Naha Breakwater	36	36	36	36	36	36	36	36
2013	First Shinko Breakwater	30	30	30	30	30	30	30	30
2013	First Urasoe Breakwater	18	18	18	18	18	18	18	18
2013	Naha Breakwater	36	36	36	36	36	36	36	36
2014	First Shinko Breakwater	31	31	31	31	31	31	31	31
2014	First Urasoe Breakwater	27	27	27	27	27	27	27	27
2014	Naha Breakwater	21	21	21	21	21	21	21	21
2015	First Shinko Breakwater	30	30	30	30	30	30	30	30
2015	First Urasoe Breakwater	18	18	18	18	18	18	18	18
2015	Naha Breakwater	36	36	36	36	36	36	36	36
2017	First Shinko Breakwater	30	30	30	30	30	30	30	30
2017	First Urasoe Breakwater	18	18	18	18	18	18	18	18
2017	Naha Breakwater	36	36	36	36	36	36	36	36

Supplementary Table S21. Number of data points (*n*) for coral cover by taxon on natural reefs in Naha Port, Japan, for each fiscal research year.

Fiscal research year	Location	All hard corals (*n*)	*Acropora* (*n*)	*Pocillopora* (*n*)	*Montipora* (*n*)	*Porites* (*n*)	Faviidae (*n*)	*Millepora* (*n*)	Other hard corals (*n*)
1994	Jijaka-se reef and Kan-no-se reef	3	3	3	3	3	3	3	3
1994	Asa-no-se reef and Gima-no-se reef	2	2	2	2	2	2	2	2
1994	Naha airport reef	2	2	2	2	2	2	2	2
1994	Naha dock reef	1	1	1	1	1	1	1	1
1994	Shinko dock reef	1	1	1	1	1	1	1	1
1994	Natural reef on the inner side of the First Shinko Breakwater	3	3	3	3	3	3	3	3
1996	Kano-no-se reef	6	6	6	6	6	6	6	6
1996	Asa-no-se reef	3	3	3	3	3	3	3	3
1996	Urasoe reef L-1	19	19	19	19	19	19	19	19
1996	Urasoe reef L-2	14	14	14	14	14	14	14	14
1996	Jijaka-se reef	9	9	9	9	9	9	9	9
2000	Kano-no-se reef	3	3	3	3	3	3	3	3
2000	Asa-no-se reef	3	3	3	3	3	3	3	3
2000	Jijaka-se reef	3	3	3	3	3	3	3	3
2001	Kano-no-se reef	20	20	20	20	20	20	20	20
2001	Asa-no-se reef	8	8	8	8	8	8	8	8
2001	Urasoe reef L-1	27	27	27	27	27	27	27	27
2001	Urasoe reef L-2	18	18	18	18	18	18	18	18
2002	Kano-no-se reef	20	20	20	20	20	20	20	20
2002	Asa-no-se reef	8	8	8	8	8	8	8	8
2002	Urasoe reef L-1	27	27	27	27	27	27	27	27
2002	Urasoe reef L-2	18	18	18	18	18	18	18	18
2003	Kano-no-se reef	20	20	20	20	20	20	20	20
2003	Asa-no-se reef	8	8	8	8	8	8	8	8
2003	Urasoe reef L-1	27	27	27	27	27	27	27	27
2003	Urasoe reef L-2	18	18	18	18	18	18	18	18
2004	Kano-no-se reef	20	20	20	20	20	20	20	20
2004	Asa-no-se reef	8	8	8	8	8	8	8	8
2004	Urasoe reef L-1	27	27	27	27	27	27	27	27
2004	Urasoe reef L-2	18	18	18	18	18	18	18	18
2005	Kano-no-se reef	20	20	20	20	20	20	20	20
2005	Asa-no-se reef	8	8	8	8	8	8	8	8
2005	Urasoe reef L-1	27	27	27	27	27	27	27	27
2005	Urasoe reef L-2	18	18	18	18	18	18	18	18
2006	Kano-no-se reef	45	45	45	45	45	45	45	45
2006	Asa-no-se reef	22	22	22	22	22	22	22	22
2006	Urasoe reef	65	65	65	65	65	65	65	65
2006	Urasoe reef (Patch)	5	5	5	5	5	5	5	5
2006	Urasoe reef L-1	27	27	27	27	27	27	27	27

2006	Urasoe reef L-2	18	18	18	18	18	18	18	18
2006	Jijaka-se reef	56	56	56	56	56	56	56	56
2007	Kano-no-se reef	20	20	20	20	20	20	20	20
2007	Asa-no-se reef	8	8	8	8	8	8	8	8
2007	Urasoe reef L-1	27	27	27	27	27	27	27	27
2007	Urasoe reef L-2	18	18	18	18	18	18	18	18
2007	Jijaka-se reef	20	20	20	20	20	20	20	20
2008	Kano-no-se reef	20	20	20	20	20	20	20	20
2008	Asa-no-se reef	8	8	8	8	8	8	8	8
2008	Urasoe reef L-1	27	27	27	27	27	27	27	27
2008	Urasoe reef L-2	18	18	18	18	18	18	18	18
2008	Jijaka-se reef	20	20	20	20	20	20	20	20
2009	Kano-no-se reef	20	20	20	20	20	20	20	20
2009	Asa-no-se reef	8	8	8	8	8	8	8	8
2009	Urasoe reef L-1	27	27	27	27	27	27	27	27
2009	Urasoe reef L-2	18	18	18	18	18	18	18	18
2009	Jijaka-se reef	20	20	20	20	20	20	20	20
2010	Kano-no-se reef	46	46	46	46	46	46	46	46
2010	Asa-no-se reef	16	16	16	16	16	16	16	16
2010	Urasoe reef	73	73	73	73	73	73	73	73
2010	Urasoe reef L-1	27	27	27	27	27	27	27	27
2010	Urasoe reef L-2	18	18	18	18	18	18	18	18
2010	Jijaka-se reef	56	56	56	56	56	56	56	56
2011	Kano-no-se reef	20	20	20	20	20	20	20	20
2011	Asa-no-se reef	8	8	8	8	8	8	8	8
2011	Urasoe reef	16	16	16	16	16	16	16	16
2011	Urasoe reef L-1	27	27	27	27	27	27	27	27
2011	Urasoe reef L-2	18	18	18	18	18	18	18	18
2011	Jijaka-se reef	20	20	20	20	20	20	20	20
2012	Kano-no-se reef	20	20	20	20	20	20	20	20
2012	Asa-no-se reef	8	8	8	8	8	8	8	8
2012	Urasoe reef	16	16	16	16	16	16	16	16
2012	Urasoe reef L-1	27	27	27	27	27	27	27	27
2012	Urasoe reef L-2	18	18	18	18	18	18	18	18
2012	Jijaka-se reef	20	20	20	20	20	20	20	20
2013	Kano-no-se reef	20	20	20	20	20	20	20	20
2013	Asa-no-se reef	8	8	8	8	8	8	8	8
2013	Urasoe reef	16	16	16	16	16	16	16	16
2013	Urasoe reef L-1	27	27	27	27	27	27	27	27
2013	Urasoe reef L-2	18	18	18	18	18	18	18	18
2013	Jijaka-se reef	20	20	20	20	20	20	20	20
2014	Kano-no-se reef	20	20	20	20	20	20	20	20

2014	Asa-no-se reef	8	8	8	8	8	8	8	8
2014	Urasoe reef L-1	27	27	27	27	27	27	27	27
2014	Urasoe reef L-2	18	18	18	18	18	18	18	18
2014	Jijaka-se reef	20	20	20	20	20	20	20	20
2015	Kano-no-se reef	45	45	45	45	45	45	45	45
2015	Asa-no-se reef	19	19	19	19	19	19	19	19
2015	Urasoe reef	83	83	83	83	83	83	83	83
2015	Urasoe reef (Patch)	8	8	8	8	8	8	8	8
2015	Urasoe reef L-1	27	27	27	27	27	27	27	27
2015	Urasoe reef L-2	18	18	18	18	18	18	18	18
2015	Jijaka-se reef	56	56	56	56	56	56	56	56
2016	Kano-no-se reef	20	20	20	20	20	20	20	20
2016	Asa-no-se reef	8	8	8	8	8	8	8	8
2016	Urasoe reef	12	12	12	12	12	12	12	12
2016	Urasoe reef L-1	27	27	27	27	27	27	27	27
2016	Urasoe reef L-2	18	18	18	18	18	18	18	18
2016	Jijaka-se reef	20	20	20	20	20	20	20	20
2017	Kano-no-se reef	20	20	20	20	20	20	20	20
2017	Asa-no-se reef	8	8	8	8	8	8	8	8
2017	Urasoe reef	12	12	12	12	12	12	12	12
2017	Urasoe reef L-1	27	27	27	27	27	27	27	27
2017	Urasoe reef L-2	18	18	18	18	18	18	18	18
2017	Jijaka-se reef	20	20	20	20	20	20	20	20
2018	Kano-no-se reef	20	20	20	20	20	20	20	20
2018	Asa-no-se reef	8	8	8	8	8	8	8	8
2018	Urasoe reef	12	12	12	12	12	12	12	12
2018	Urasoe reef L-1	27	27	27	27	27	27	27	27
2018	Urasoe reef L-2	18	18	18	18	18	18	18	18
2018	Jijaka-se reef	20	20	20	20	20	20	20	20

Supplementary Table S22. Variation inflation factors (VIFs) for covariates.

Covariates	2000–2018	2000–2004
Substrate	1.06	1.01
Research year	1.06	1.01
Depth	1.00	1.00

Supplementary Table S23. Gelman-Rubin statistics for parameters of coral-cover models.

Parameter	Total hard coral (2000-2018)	*Acropora* (2000-2018)	*Pocillopora* (2000-2018)	*Montipora* (2000-2018)	*Porites* (2000-2018)	Faviidae (2000-2018)	*Millepora* (2000-2018)	Other hard corals (2000-2018)	Total hard coral (2000-2004)	*Acropora* (2000-2004)	*Pocillopora* (2000-2004)	*Montipora* (2000-2004)	*Porites* (2000-2004)	Faviidae (2000-2004)	*Millepora* (2000-2004)	Other hard corals (2000-2004)
location_Naha Breakwater	1.00	1.00	1.00	1.00	1.00	1.00	1.00	1.00	1.00	1.00	1.01	1.00	1.00	1.00	1.00	1.00
location_First Shinko Breakwater	1.00	1.00	1.00	1.00	1.00	1.00	1.00	1.00	1.00	1.00	1.01	1.00	1.00	1.00	1.00	1.00
location_Natural reef on the inner side of the First Shinko Breakwater	1.00	1.00	1.00	1.00	1.00	1.00	1.00	1.00	1.00	1.00	1.01	1.00	1.00	1.00	1.00	1.00
location_First Urasoe Breakwater	1.00	1.00	1.00	1.00	1.00	1.00	1.00	1.00	1.00	1.00	1.02	1.00	1.00	1.00	1.00	1.00
location_Asa-no-se reef	1.00	1.00	1.00	1.00	1.00	1.00	1.00	1.00	1.00	1.00	1.01	1.00	1.00	1.00	1.00	1.00
location_Kan-no-se reef	1.00	1.00	1.00	1.00	1.00	1.00	1.00	1.00	1.00	1.00	1.02	1.00	1.00	1.00	1.00	1.00
location_Jijaka-se reef	1.00	1.00	1.00	1.00	1.00	1.00	1.00	1.00	1.00	1.00	1.00	1.00	1.00	1.00	1.00	1.01
location_Urasoe reef	1.00	1.00	1.00	1.00	1.00	1.00	1.00	1.00	1.00	1.00	1.00	1.00	1.00	1.00	1.00	1.01
location_Urasoe reef (Patch)	1.00	1.00	1.00	1.00	1.00	1.00	1.00	1.00	1.00	1.00	1.01	1.00	1.00	1.00	1.00	1.00
location_Urasoe reef L-1	1.00	1.00	1.00	1.00	1.00	1.00	1.00	1.00	1.00	1.00	1.02	1.00	1.00	1.00	1.00	1.00
location_Urasoe reef L-2	1.00	1.00	1.00	1.00	1.00	1.00	1.00	1.00	1.00	1.00	1.01	1.00	1.00	1.00	1.00	1.00
method_Quadrat	1.00	1.00	1.00	1.00	1.00	1.00	1.00	1.00	1.00	1.00	1.01	1.01	1.00	1.00	1.00	1.01
method_Snorkel	1.00	1.00	1.00	1.00	1.00	1.00	1.00	1.00	1.00	1.00	1.01	1.01	1.00	1.00	1.00	1.01
method_Spot check	1.00	1.00	1.00	1.00	1.00	1.00	1.00	1.00	1.00	1.00	1.05	1.02	1.00	1.00	1.00	1.01
method_Belt transect	1.00	1.00	1.00	1.00	1.00	1.00	1.00	1.00	1.00	1.00	1.02	1.00	1.00	1.00	1.00	1.01
method_Manta	1.00	1.00	1.00	1.00	1.00	1.00	1.00	1.00	1.00	1.00	1.05	1.01	1.00	1.00	1.00	1.01
intercept	1.00	1.00	1.00	1.00	1.00	1.00	1.00	1.00	1.00	1.00	1.03	1.00	1.00	1.00	1.00	1.01
beta_substrate	1.00	1.00	1.00	1.00	1.00	1.00	1.00	1.00	1.00	1.00	1.04	1.00	1.00	1.00	1.00	1.00
beta_depth	1.00	1.00	1.00	1.00	1.00	1.00	1.00	1.00	1.00	1.00	1.01	1.00	1.00	1.00	1.00	1.00
beta_research_year	1.00	1.00	1.00	1.00	1.00	1.00	1.00	1.00	1.00	1.00	1.00	1.00	1.00	1.00	1.00	1.01
beta_substrate*depth	1.00	1.00	1.00	1.00	1.00	1.00	1.00	1.00	1.00	1.00	1.01	1.00	1.00	1.00	1.00	1.00
beta_substrate*research_year	1.00	1.00	1.00	1.00	1.00	1.00	1.00	1.00	1.00	1.00	1.00	1.00	1.00	1.00	1.00	1.00
sd_location	1.00	1.00	1.00	1.00	1.00	1.00	1.00	1.00	1.00	1.00	1.01	1.00	1.00	1.00	1.00	1.00
sd_method	1.00	1.00	1.00	1.00	1.00	1.00	1.00	1.00	1.00	1.00	1.08	1.03	1.00	1.00	1.00	1.01
epsilon	1.00	1.00	1.00	1.00	1.00	1.00	1.00	1.00	1.00	1.00	1.00	1.00	1.00	1.00	1.00	1.00

Supplementary Table S24. Bayesian R^2 values for parameters of coral-cover models.

	Bayesian R^2	Standard deviation
Total hard coral (2000-2018)	0.312	0.004
Acropora (2000-2018)	0.308	0.004
Pocillopora (2000-2018)	0.317	0.004
Montipora (2000-2018)	0.330	0.005
Porites (2000-2018)	0.329	0.005
Faviidae (2000-2018)	0.328	0.005
Millepora (2000-2018)	0.387	0.005
Other hard corals (2000-2018)	0.324	0.004
Total hard coral (2000-2004)	0.347	0.011
Acropora (2000-2004)	0.391	0.011
Pocillopora (2000-2004)	0.350	0.010
Montipora (2000-2004)	0.333	0.011
Porites (2000-2004)	0.327	0.010
Faviidae (2000-2004)	0.328	0.010
Millepora (2000-2004)	0.336	0.011
Other hard corals (2000-2004)	0.320	0.010

Supplementary Table S25. Standard deviation of the model prediction (ε) and random effects (s_{site} and s_{method})

Parameter		Mean	SD	Lower limit of 95% HDI	Lower limit of 80% HDI	Upper limit of 80% HDI	Upper limit of 95% HDI	Gelman-Rubin statistics
	s_{method}	0.619	0.290	0.266	0.326	0.801	1.094	1.00
Total hard coral	s_{site}	0.751	0.209	0.422	0.482	0.925	1.167	1.00
(2000–2018)	ε	1.488	0.019	1.453	1.463	1.511	1.525	1.00
	s_{method}	0.349	0.166	0.126	0.165	0.460	0.651	1.00
Acropora	s_{site}	0.943	0.262	0.542	0.610	1.172	1.477	1.00
(2000–2018)	ε	1.392	0.017	1.358	1.370	1.414	1.426	1.00
	s_{method}	0.203	0.098	0.068	0.091	0.276	0.394	1.00
Pocillopora	s_{site}	0.908	0.249	0.522	0.580	1.115	1.412	1.00
(2000–2018)	ε	1.315	0.017	1.283	1.294	1.336	1.348	1.00
	s_{method}	0.260	0.115	0.102	0.128	0.339	0.486	1.00
Montipora	s_{site}	0.262	0.074	0.146	0.165	0.329	0.410	1.00
(2000–2018)	ε	1.004	0.013	0.979	0.989	1.021	1.028	1.00
	s_{method}	0.319	0.142	0.129	0.160	0.418	0.584	1.00
Porites	s_{site}	0.252	0.076	0.132	0.154	0.318	0.404	1.00
(2000–2018)	ε	1.112	0.014	1.084	1.094	1.129	1.137	1.00
	s_{method}	0.624	0.265	0.273	0.331	0.805	1.130	1.00
Faviidae	s_{site}	0.621	0.169	0.344	0.394	0.765	0.956	1.00
(2000–2018)	ε	1.133	0.014	1.105	1.115	1.151	1.160	1.00
	s_{method}	0.218	0.095	0.085	0.106	0.289	0.403	1.00
Millepora	s_{site}	0.516	0.145	0.286	0.331	0.641	0.791	1.00
(2000–2018)	ε	0.983	0.012	0.960	0.968	0.999	1.007	1.00
	s_{method}	0.628	0.269	0.270	0.326	0.797	1.132	1.00
Other hard corals	s_{site}	0.474	0.135	0.269	0.302	0.585	0.729	1.00
(2000–2018)	ε	0.844	0.010	0.824	0.832	0.858	0.864	1.00
	s_{method}	0.484	0.342	0.099	0.166	0.677	1.112	1.00
Total hard coral	s_{site}	1.057	0.355	0.536	0.612	1.355	1.776	1.00
(2000–2004)	ε	1.513	0.039	1.435	1.463	1.562	1.589	1.00
	s_{method}	0.494	0.356	0.074	0.161	0.694	1.117	1.00
Acropora	s_{site}	0.986	0.327	0.496	0.572	1.244	1.635	1.00
(2000–2004)	ε	1.368	0.035	1.302	1.321	1.410	1.439	1.00
	s_{method}	0.269	0.349	0.004	0.004	0.407	0.749	1.08
Pocillopora	s_{site}	1.249	0.405	0.629	0.778	1.608	2.017	1.01
(2000–2004)	ε	1.261	0.032	1.197	1.221	1.302	1.323	1.00
	s_{method}	0.113	0.154	0.002	0.002	0.182	0.354	1.03
Montipora	s_{site}	0.220	0.091	0.071	0.103	0.300	0.397	1.00
(2000–2004)	ε	0.785	0.020	0.745	0.761	0.812	0.823	1.00
	s_{method}	0.292	0.227	0.015	0.033	0.406	0.680	1.00
Porites	s_{site}	0.437	0.157	0.203	0.244	0.564	0.738	1.00
(2000–2004)	ε	0.945	0.024	0.898	0.913	0.975	0.995	1.00
	s_{method}	0.468	0.329	0.123	0.168	0.629	1.042	1.00
Faviidae	s_{site}	0.800	0.291	0.369	0.446	1.034	1.366	1.00
(2000–2004)	ε	0.850	0.022	0.808	0.821	0.876	0.893	1.00
	s_{method}	0.580	0.396	0.147	0.196	0.766	1.291	1.00
Millepora	s_{site}	0.445	0.162	0.200	0.251	0.587	0.768	1.00
(2000–2004)	ε	0.872	0.023	0.829	0.844	0.901	0.918	1.00
	s_{method}	0.038	0.058	0.001	0.001	0.058	0.132	1.01
Other hard corals	s_{site}	0.662	0.258	0.262	0.352	0.876	1.135	1.00
(2000–2004)	ε	0.646	0.016	0.614	0.624	0.667	0.678	1.00

Supplementary Table S26. Bayesian R^2 values for models for coral cover and the number of coral colonies.

		Bayesian R^2	Standard deviation
Models for coral cover	Total hard coral (1-18)	0.346	0.020
	Acropora (1-18)	0.349	0.021
	Pocillopora (1-18)	0.325	0.018
	Montipora (1-18)	0.350	0.021
	Porites (1-18)	0.343	0.021
	Faviidae (1-18)	0.330	0.018
	Millepora (1-18)	0.324	0.019
	Other hard corals (1-18)	0.338	0.021
	Total hard coral (1-6)	0.374	0.025
	Acropora (1-6)	0.380	0.027
	Pocillopora (1-6)	0.366	0.024
	Montipora (1-6)	0.367	0.029
	Porites (1-6)	0.358	0.027
	Faviidae (1-6)	0.358	0.026
	Millepora (1-6)	0.359	0.029
	Other hard corals (1-6)	0.357	0.028
Models for the number of coral colonies	Total hard coral (1-18)	0.303	0.028
	Acropora (1-18)	0.301	0.033
	Pocillopora (1-18)	0.287	0.027
	Montipora (1-18)	0.233	0.027
	Porites (1-18)	0.196	0.025
	Faviidae (1-18)	0.229	0.028
	Millepora (1-18)	0.271	0.025
	Other hard corals (1-18)	0.237	0.022
	Total hard coral (1-6)	0.314	0.037
	Acropora (1-6)	0.316	0.043
	Pocillopora (1-6)	0.343	0.042
	Montipora (1-6)	0.231	0.035
	Porites (1-6)	0.190	0.036
	Faviidae (1-6)	0.194	0.040
	Millepora (1-6)	0.263	0.033
	Other hard corals (1-6)	0.235	0.031

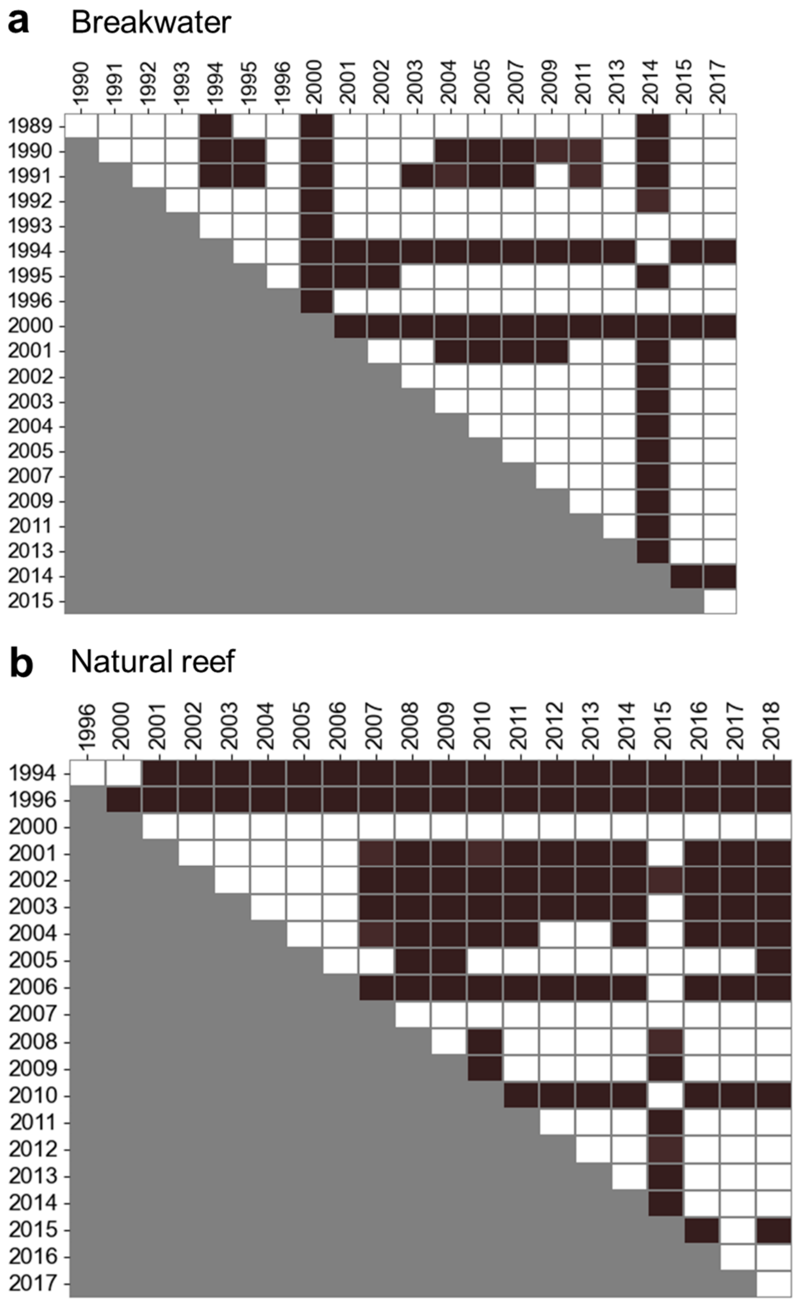

Supplementary Fig. S1 Results of post-hoc testing (pairwise PERMANOVAs) for similarity of community structure between survey years on (a) breakwaters and (b) natural reefs. Black cells show significant differences ($P < 0.05$) and white cells show no significant differences ($P \geq 0.05$). Overlapping combinations are greyed out.

Number of coral colonies

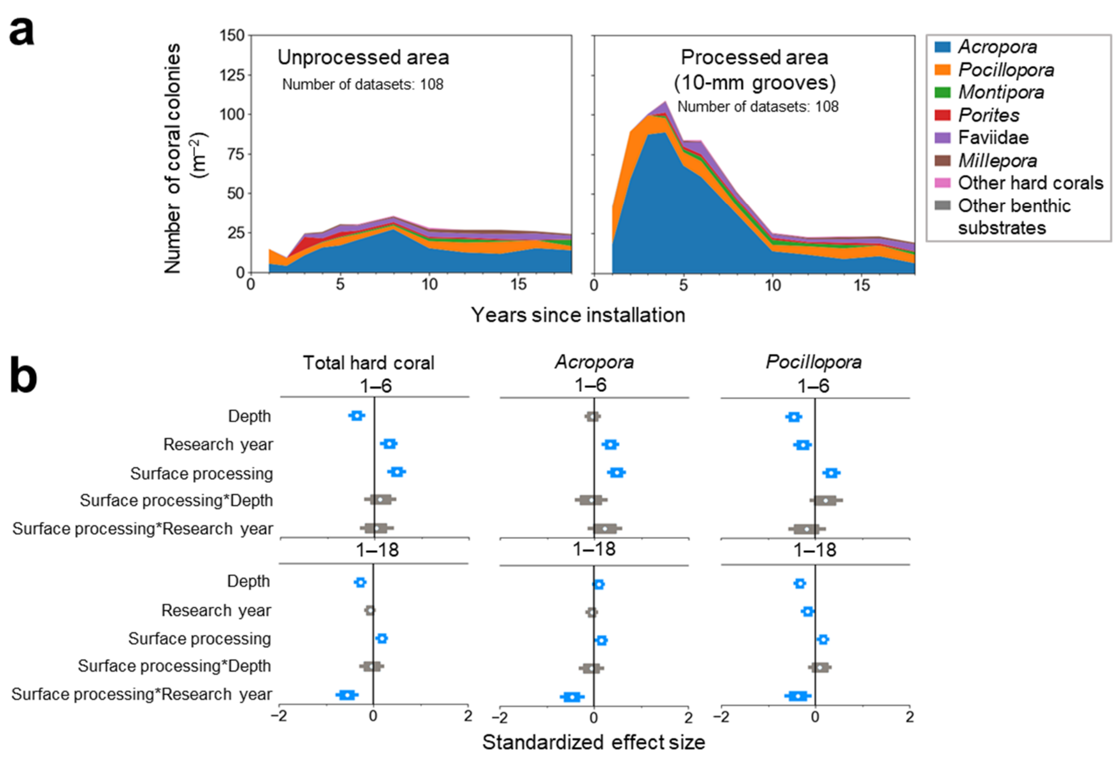

Supplementary Fig. S2 Effect of surface processing of wave-dissipating blocks on number of coral colonies from 2000 to 2017. (a) Temporal dynamics in mean number of coral colonies on unprocessed and processed areas. (b) Standardized effect size of explanatory variables of number of coral colonies, from a Bayesian hierarchical model for the early (1–6 years after construction) and the entire (1–18 years after construction) periods. The explanation regarding the legends and the sign of effect size is the same as in Fig. 2c. Please refer to Eq. (3) for a detailed explanation.

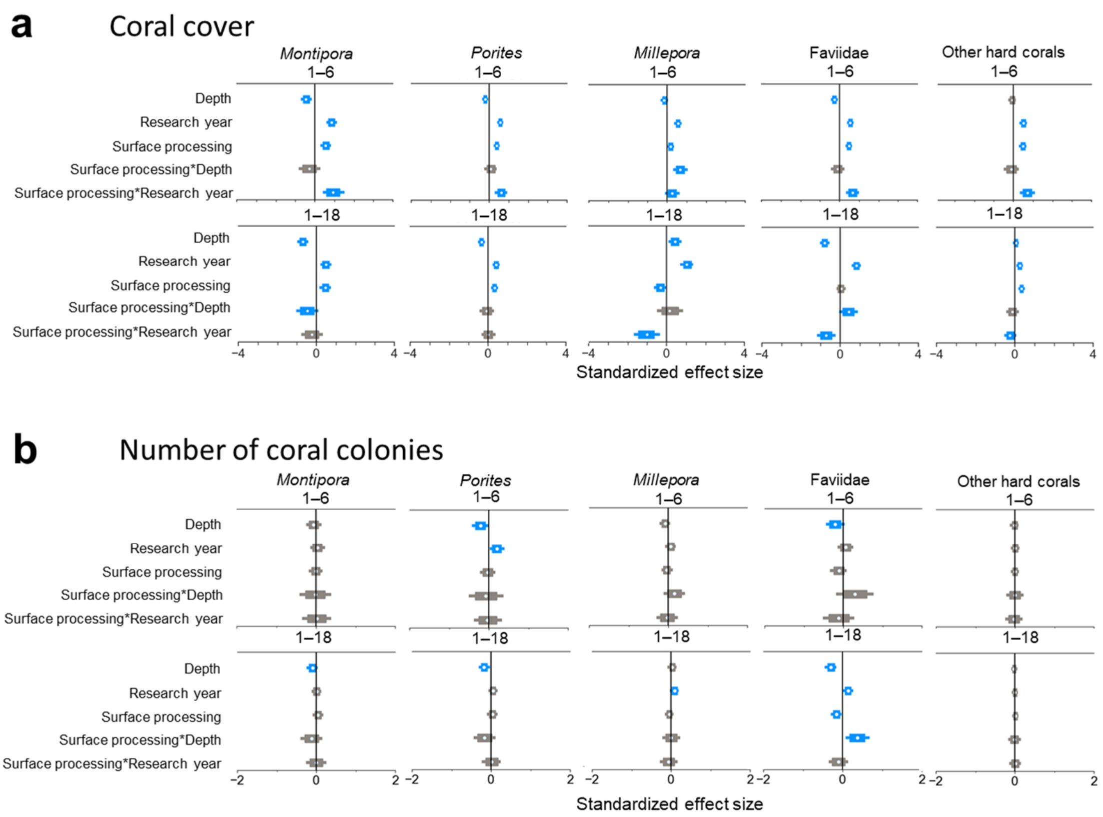

Supplementary Fig. S3 Effect of surface processing of wave-dissipating blocks on coral cover and number of coral colonies from 2000 to 2017. Standardized effect size of explanatory variables of (a) logarithm of the ratio of coral cover to other benthic cover and (b) number of coral colonies, from a Bayesian hierarchical model for the early (1–6 years after construction) and the entire (1–18 years after construction) period for *Montipora*, *Porites*, *Millepora*, Faviidae and other hard corals. White circles represent the median, and thin and thick horizontal lines represent the 95% and 80% highest density intervals (HDIs), respectively. Blue lines indicate that the 80% HDI does not overlap with 0, suggesting a credible trend, and grey lines indicate that the 80% HDI overlaps with 0, suggesting a less credible trend. For the effect of surface processing, a positive effect size indicates a relatively

positive effect on the coral cover in processed areas; a negative effect size indicates a relatively positive effect on the coral cover in unprocessed areas. Please refer to Eq. (2) and Eq. (3) for a detailed explanation of the Bayesian hierarchical models.

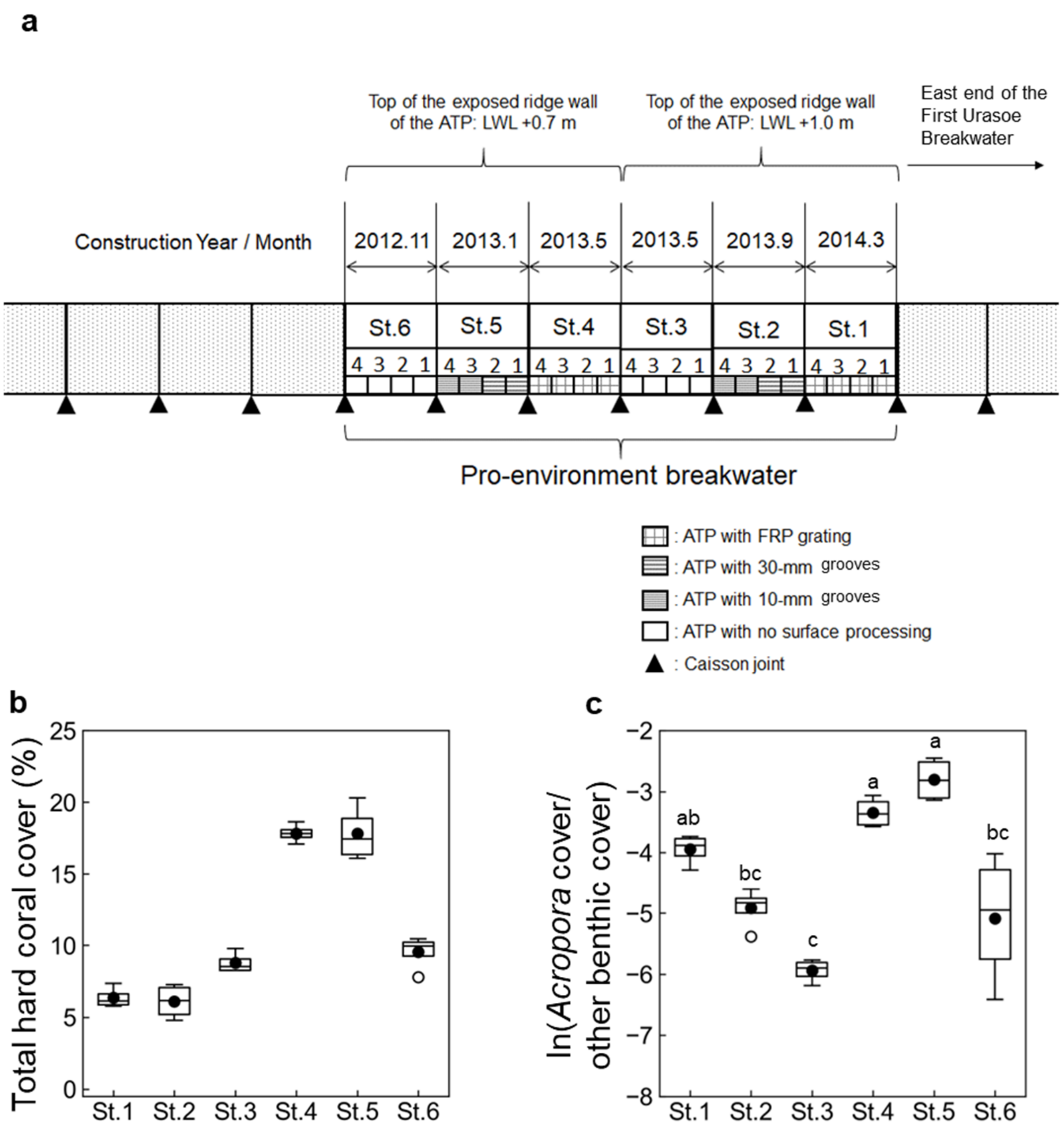

Supplementary Fig. S4 Attributes for artificial tide pools (ATPs). (a) Construction progression of the pro-environment breakwater appended to near the eastern end of the First Urasoe Breakwater and the caissons and ATPs in Naha Port, Okinawa, Japan. (b) Hard-coral cover on ATPs at each station (bottoms and inner side walls) in 2018. (c) Logarithm of the ratio of *Acropora* cover to other benthic

cover of ATPs at each station (bottoms and inner side walls) in 2018. Boxes show the 25% and 75% quantiles, horizontal lines inside the boxes are median values, whiskers show maximum and minimum values except for outliers (white circles), and black circles are mean values. Sample size (*n*) for each station is 4 (4 ATPs per station). Differences between letters (a–c) in panel (c) indicate significant differences among stations (ANOVA, $P < 0.05$).

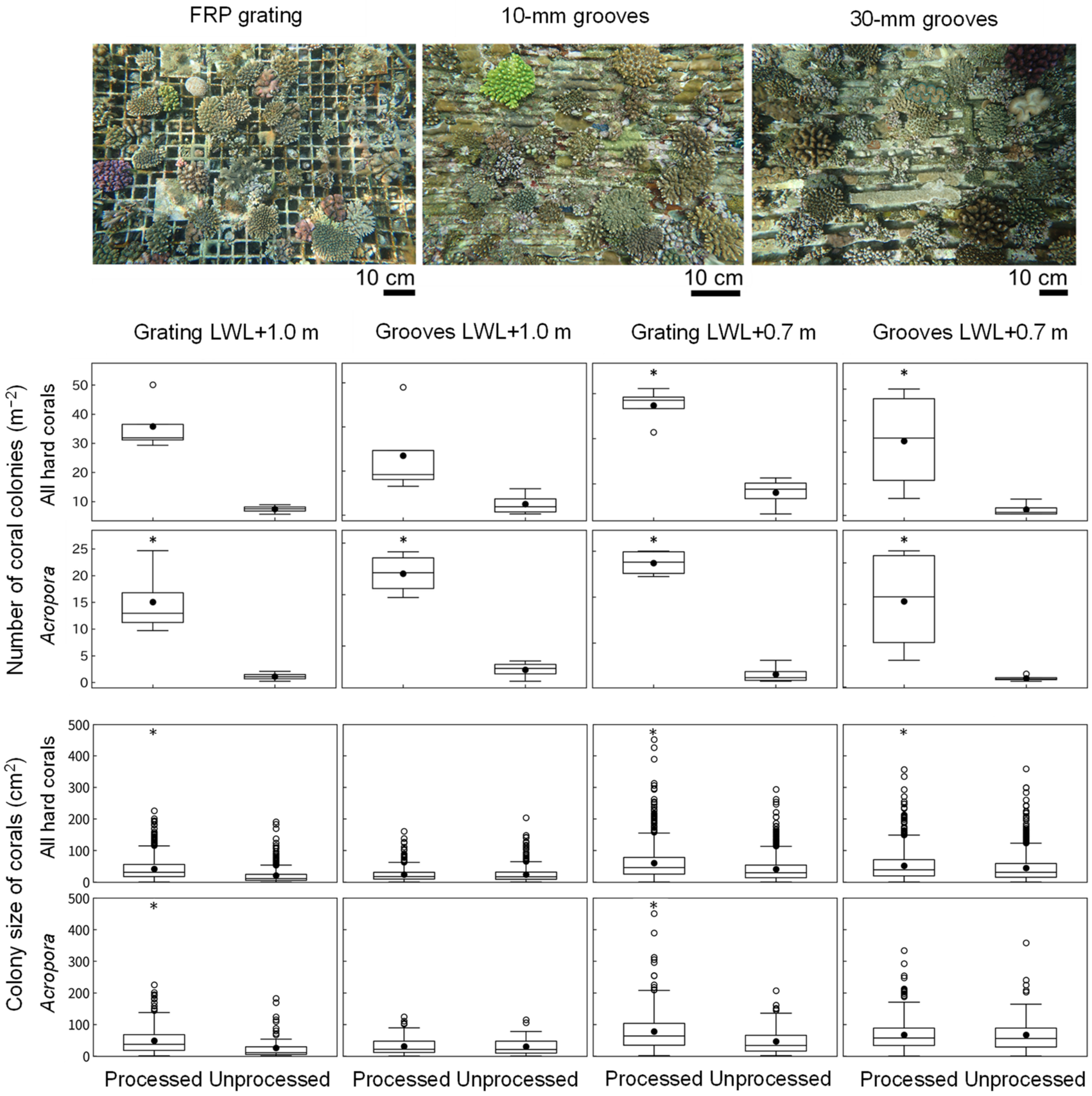

Supplementary Fig. S5 Coral community enhancement by surface processing of artificial tide pools (ATPs). The number of coral colonies and size of coral colonies on the processed and unprocessed areas of the bottom of ATPs with FRP grating or grooves (10-mm or 30-mm) are shown for two installation depths: low water level (LWL) +1.0 m and +0.7 m. Boxes show the 25% and 75% quantiles; horizontal lines inside the boxes are median values; whiskers show maximum and minimum values; black circles are mean values; white circles are outliers. Where there is a

significant difference between the processed and unprocessed areas (paired *t*-test or Wilcoxon signed rank test for number of coral colonies; Welch's *t*-test or Wilcoxon rank sum test for coral colony size; $P < 0.05$), the higher value is marked above with an asterisk. Photographs of surface-processed areas are taken from Tanaya et al.[31].

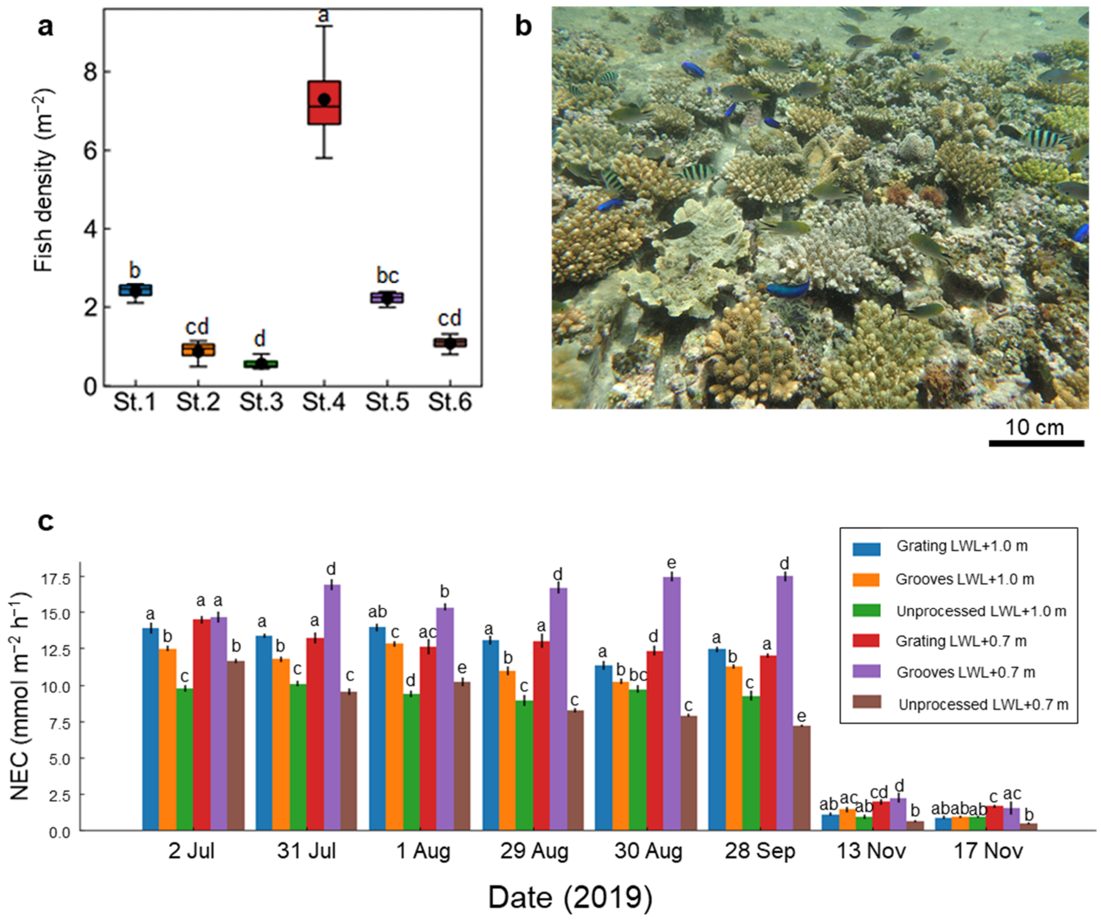

Supplementary Fig. S6 Fish community and coral growth enhancement by surface processing of artificial tide pools (ATPs). (a) Fish density. St. 1: installation depth, low water level (LWL) +1.0 m, FRP grating; St. 2: LWL +1.0 m, 10-mm or 30-mm grooves; St. 3: LWL +1.0 m, no surface processing; St. 4: LWL +0.7 m, FRP grating; St. 5: LWL +0.7 m, 10-mm or 30-mm grooves; and St. 6: LWL +0.7 m, no surface processing. Boxes show the 25% and 75% quantiles; horizontal lines inside the boxes are median values; whiskers show maximum and minimum values; black circles are mean values. For each station, $n = 4$ (4 ATPs per station). Fish populations were normalized by the bottom area of each ATP. Letters a–d indicate significant differences among stations (ANOVA, P <

0.05). (b) Photograph showing the fish assemblage in an ATP with 30-mm grooves. (c) Net ecosystem calcification rate (NEC, mmol m^{-2} h^{-1}) at each station. NEC was measured in one ATP per station (the third ATP from the east among the four ATPs at each station). Six measurements were taken during the daytime low tide (stagnant period) in July–September and two during the nighttime low tide (stagnant period) in November. NEC was calculated by multiplying the slope of the regression line for the temporal change in total alkalinity (TA) by seawater density and mean water depth (equation [4]); mean values for NEC are shown. The error bars indicate the standard errors of the NEC due to the standard deviation of the slope of the regression line. Letters a–e indicate significant differences among stations on each measurement day (ANCOVA, $P < 0.05$).

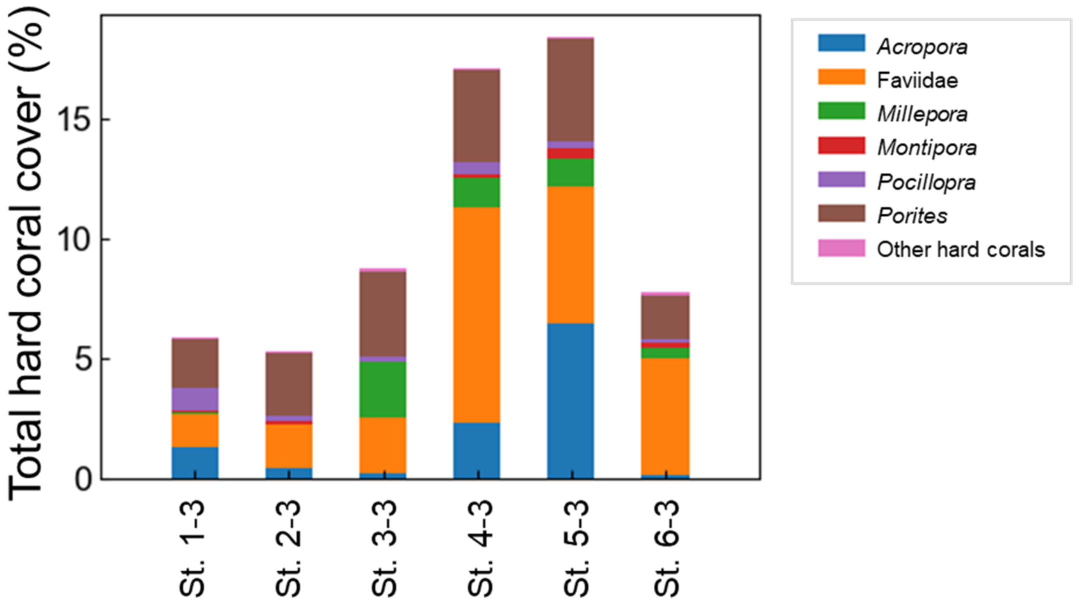

Supplementary Fig. S7 Cover of the various hard coral taxa in artificial tide pools (ATPs) where net ecosystem calcification rate was measured. The first number in the station (St.) number is the caisson number (1–6) and the second number is the ATP number (1–4; see Supplementary Fig. 3).

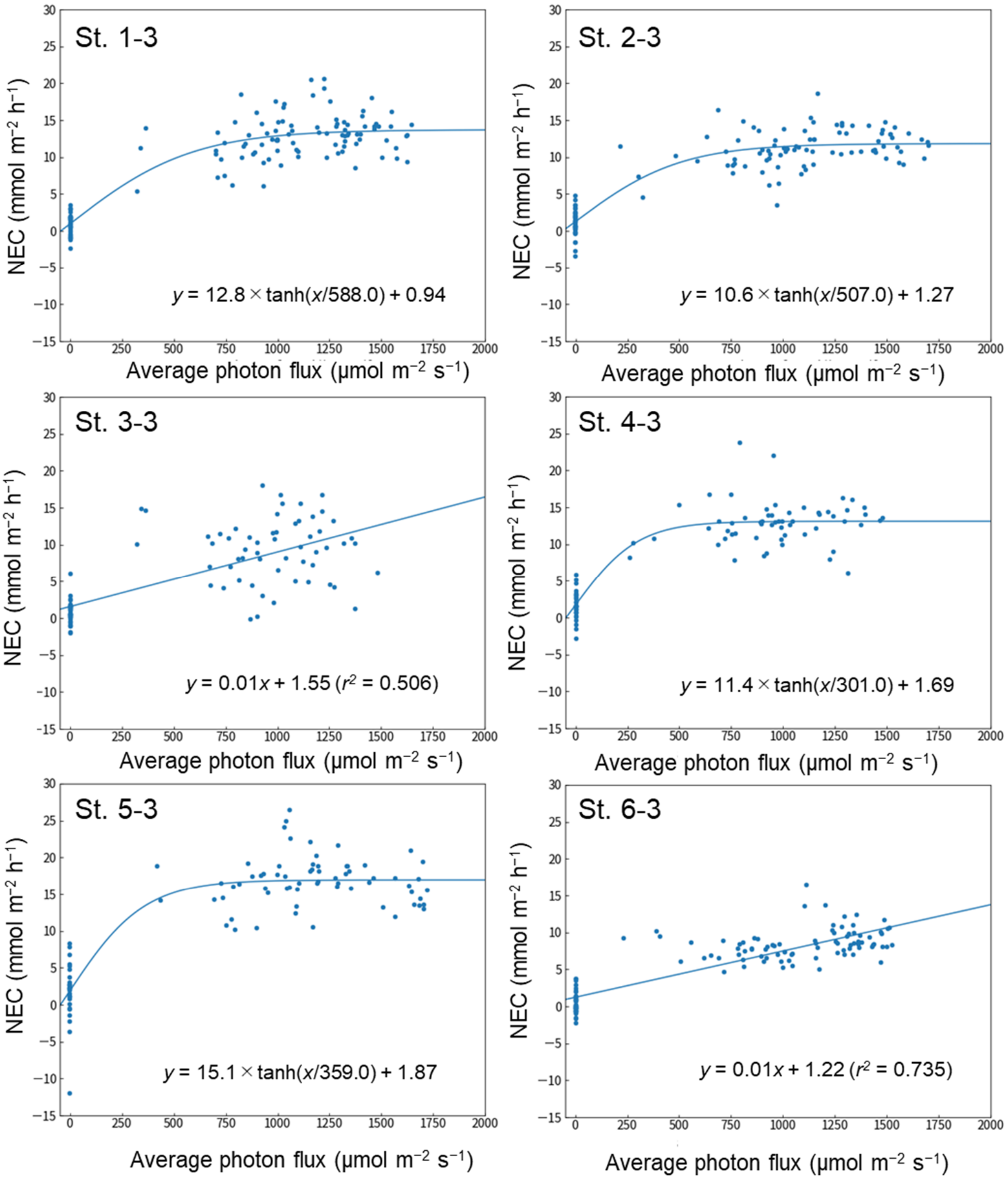

Supplementary Fig. S8 Net ecosystem calcification rate (NEC) in artificial tide pools (ATPs) versus light intensity, including the regression curves and equations. A tanh regression analysis was performed when P < 0.05 for the null hypothesis that the coefficients a, b, and c are zero in the equation $y = a \times \tanh(x/b) + c$; otherwise, linear regression was performed. In the linear regression analysis, the null hypothesis was that slope = 0.

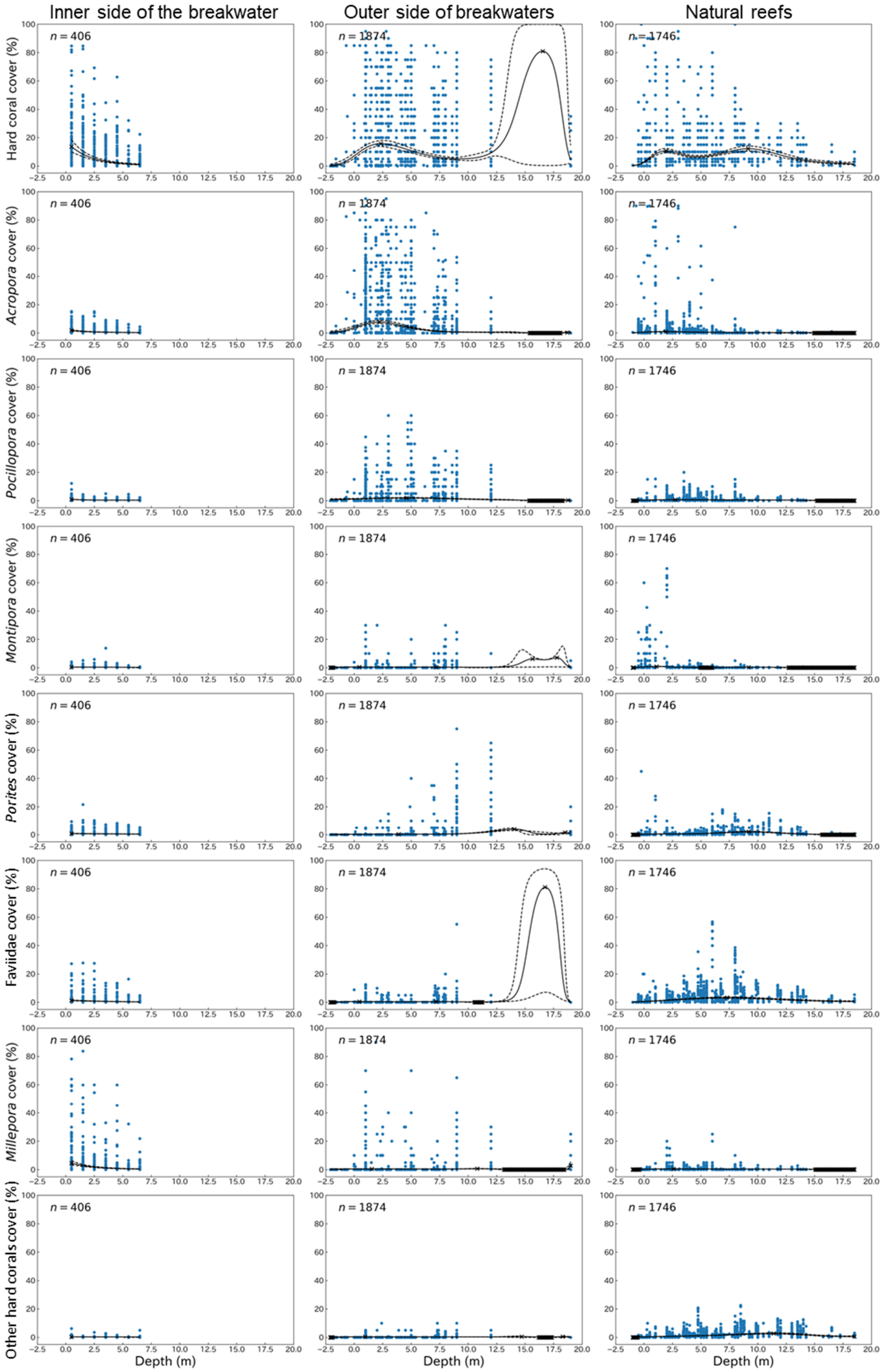

Inner side of the breakwater
Outer side of breakwaters
Natural reefs
Hard coral cover (%)
Acropora cover (%)
Pocillopora cover (%)
Montipora cover (%)
Porites cover (%)
Faviidae cover (%)
Millepora cover (%)
Other hard corals cover (%)
n = 406
n = 1874
n = 1746
Depth (m)

Supplementary Fig. S9 Coral cover on both sides (inner and outer) of the breakwaters and on natural reefs versus water depth in Naha Port, Okinawa, Japan. Solid black lines show the values estimated by the generalized additive model (GAM) and the dashed lines show 95% confidence intervals. Each blue circle represents a single measurement from a quadrat or a transect. Extreme values are shown as crosses.

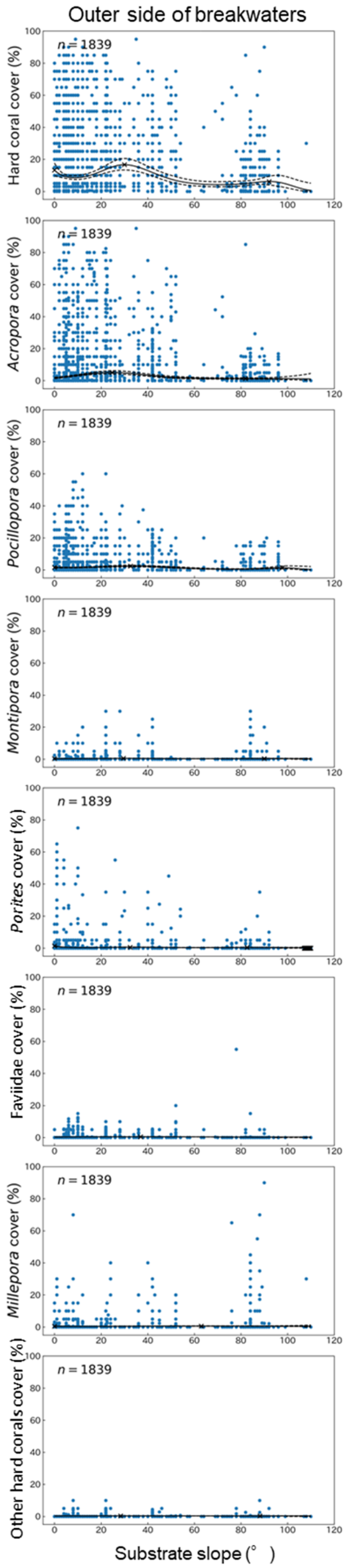

Outer side of breakwaters
n = 1839
Hard coral cover (%)
Acropora cover (%)
Pocillopora cover (%)
Montipora cover (%)
Porites cover (%)
Faviidae cover (%)
Millepora cover (%)
Other hard corals cover (%)
Substrate slope (°)

Supplementary Fig. S10 Coral cover on the outer (offshore) side of the breakwaters in Naha Port, Okinawa, Japan versus substrate slope. The solid black lines show the values estimated by the generalized additive model and the dashed lines show 95% confidence intervals. Each blue circle represents a single measurement from a quadrat. Extreme values are shown as crosses.

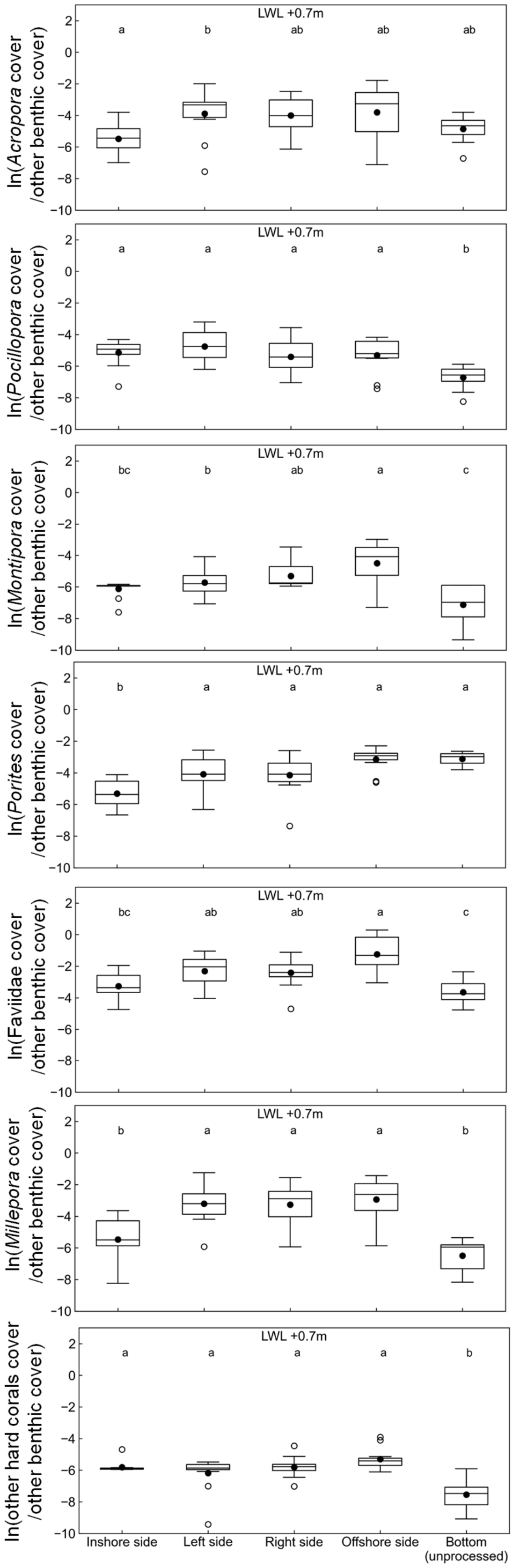

LWL +0.7m
ln(*Acropora* cover /other benthic cover)
a b ab ab ab
ln(*Pocillopora* cover /other benthic cover)
a a a a b
ln(*Montipora* cover /other benthic cover)
bc b ab a c
ln(*Porites* cover /other benthic cover)
b a a a a
ln(Faviidae cover /other benthic cover)
bc ab ab a c
ln(*Millepora* cover /other benthic cover)
b a a a b
ln(other hard corals cover /other benthic cover)
a a a a b
2
0
−2
−4
−6
−8
−10
Inshore side
Left side
Right side
Offshore side
Bottom (unprocessed)

Supplementary Fig. S11 Alr-transformed coral cover by taxon on the inner surfaces (sides and bottom) of artificial tide pools. Boxes show the 25% and 75% quantiles, horizontal lines inside the boxes are median values, whiskers show maximum and minimum values except for outliers (white circles), and black circles are mean values. The sample size (*n*) for each surface is 12. Different letters (a–d) indicate significant differences among stations (ANOVA, $P < 0.05$). LWL, low water level.

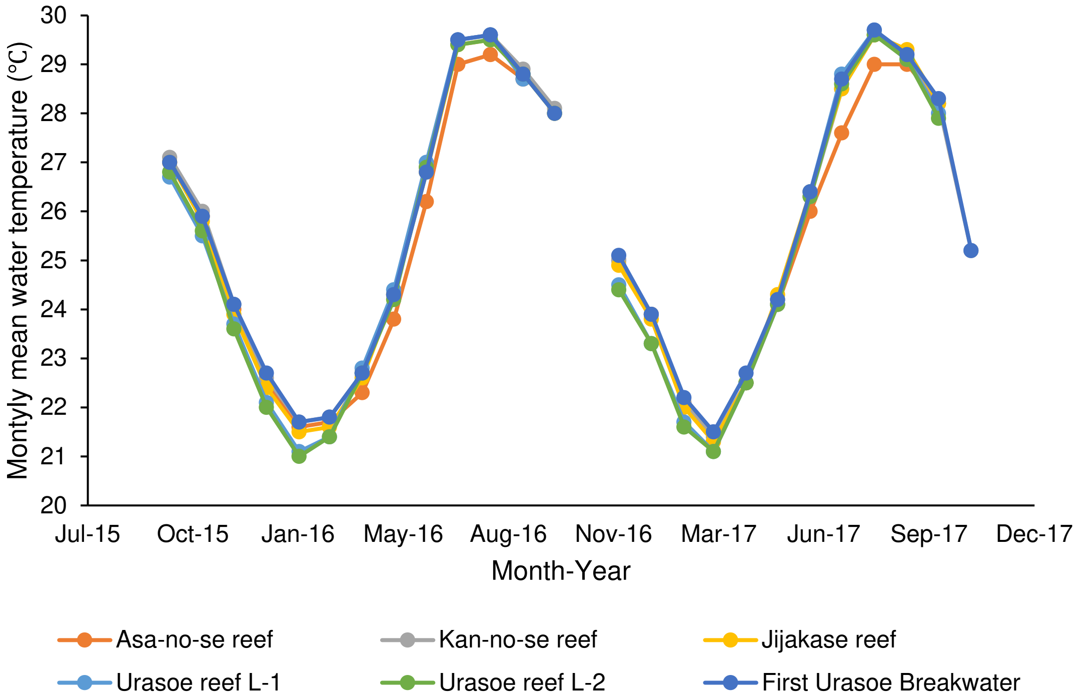

Supplementary Fig. S12 Water temperatures at the First Urasoe Breakwater and surrounding natural coral reefs in Naha Port, Okinawa, Japan, from October 2015 to November 2017. A water temperature meter (HOBO Water Temp Pro U22-001, Onset Computer Corporation, Bourne, Massachusetts, USA) was installed at low water level (LWL) –3.5 m at Asa-no-se reef and at LWL –2 m at the other locations. Water temperature was recorded at 30-min intervals.

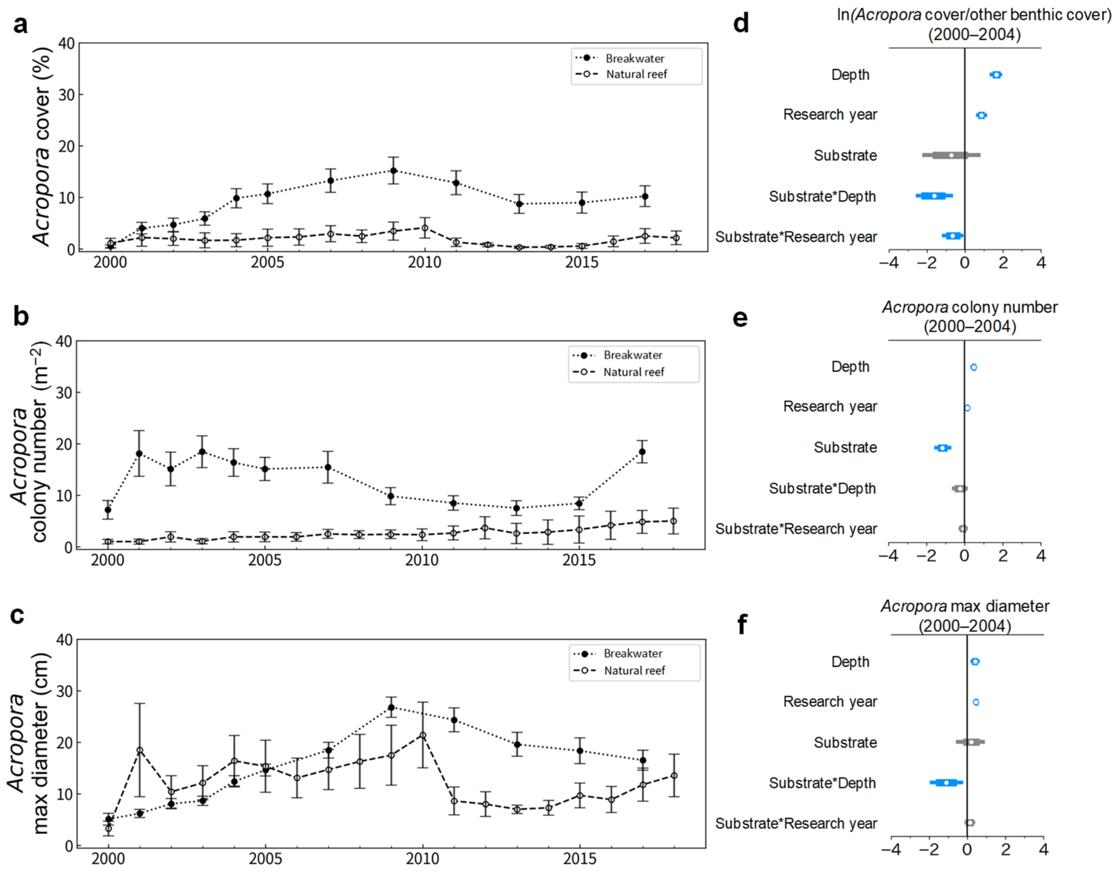

Supplementary Fig. S13 Time course of *Acropora* cover (a), number of colonies (b), and maximum colony diameter (c) on breakwaters and surrounding natural reefs in Naha Port, Okinawa, Japan after bleaching, and standardized effect size of explanatory variables of alr-transformed coral cover (d), number of colonies (e), and maximum diameter (f) from 2000 to 2004, from a Bayesian hierarchical model using only quadrat survey data in which the number of colonies was available. (a–c) The mean and SE of the coral cover, number of colonies, and maximum diameter on breakwaters (black circles) and natural reefs (white circles) for each survey year are shown. The number of data points for 2000 was 39 for breakwaters and 6 for natural coral reefs; for each survey year after 2001, the number of

data points was 57 for breakwaters and 12 for natural coral reefs. (d–f) White circles represent the median, and thin, medium, and thick horizontal lines represent the 95% and 80% highest density interval (HDI), respectively. Blue lines indicate that the 80% HDI does not overlap with 0, and grey lines indicate that the 80% HDI overlaps with 0. For the effect of substrate (i.e., breakwaters or natural reefs), a positive effect size indicates a relatively positive effect on the coral cover, number of coral colonies, and maximum diameter of colonies on natural reefs, and a negative effect size indicates a relatively positive effect on the coral cover, number of coral colonies, and maximum diameter of colonies on breakwaters. Among all covariates, the variation inflation factors (VIFs) are <1.02 and Pearson's correlation coefficients are <0.2, indicating that multicollinearity is not serious. The Gelman-Rubin statistics of each model of cover, number of colonies, and maximum colony diameter are 1.00. Bayesian R^2 (± SD) of each model of cover, number of colonies, and maximum colony diameter are 0.346 ± 0.014, 0.299 ± 0.027, 0.323 ± 0.020, respectively.

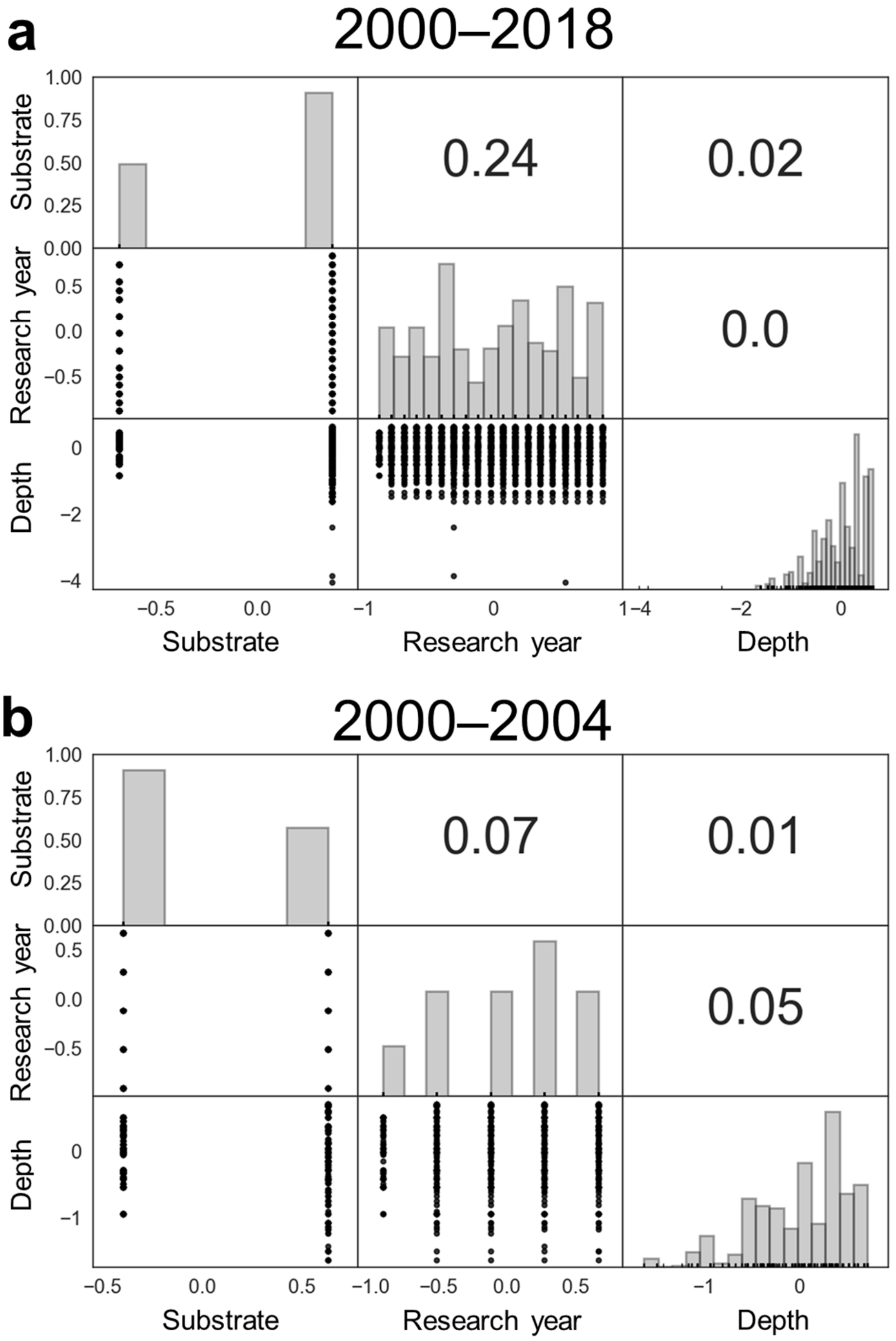

Supplementary Fig. S14 Correlation plot matrix for covariates of the coral cover model for (a) 2000–2018 and (b) 2000–2004. Pearson's correlation coefficients, histograms, and scatter plots of standardized variables are shown.

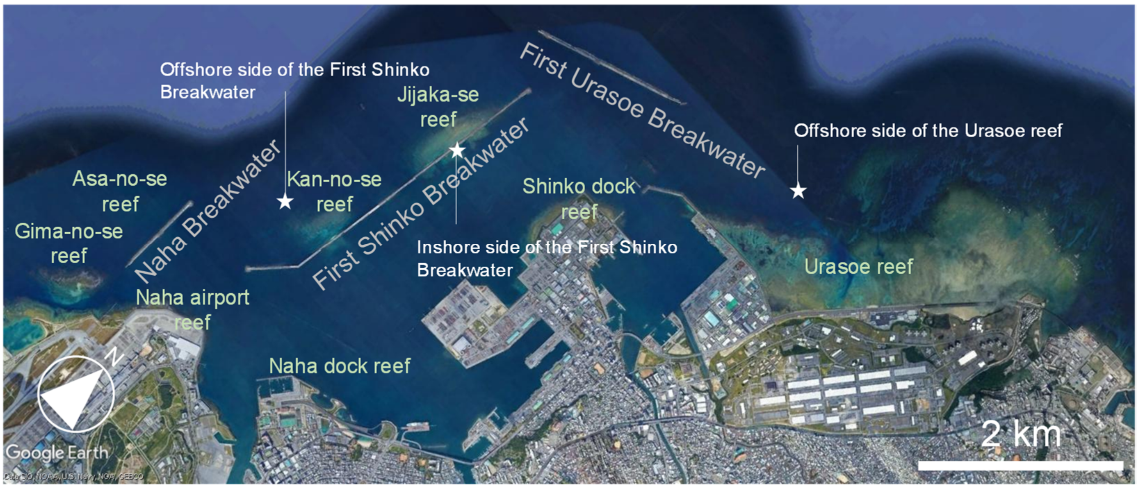

Supplementary Fig. S15 Survey points (stars) for surface seawater transparency around the First Shinko Breakwater and Urasoe reef in Naha Port, Okinawa, Japan. The map was created using the Google Earth Pro 7.3.6.10201 (https://www.google.co.jp/intl/ja/earth/about/) and annotated using the Microsoft Office PowerPoint 2016 (https://www.microsoft.com).
